# Electronic origins of the stereochemistry in β-lactam formed through the Staudinger reaction catalyzed by a nucleophile†

**DOI:** 10.1039/d3ra05286a

**Published:** 2023-11-16

**Authors:** Farideh Pahlavan, Sedigheh Saddat Moosavi, Amin Reza Zolghadr, Nasser Iranpoor

**Affiliations:** a Department of Chemistry, School of Science, Shiraz University Shiraz 71454 Iran arzolghadr@shirazu.ac.ir iranpour@shirazu.ac.ir

## Abstract

This paper evaluates the electronic effects of molecular substituents on the stereoselectivity of the umpolung Staudinger catalytic reaction. This is especially important because experimental studies on constructing the β-lactam ring, a core structure of most antibiotics, through catalyzed Staudinger reactions have been massively progressing over the last century. Yet, there is a necessity for an in-depth understanding of the reaction mechanisms to help chemists, working on the well-established discoveries, improve these to optimize the stereoselectivity and yield of synthetic methods. Access to practical and effective advancements in forming optically pure β-lactam is paramount in the field of medical chemistry. This paper specifically investigates how changing the N-protecting group in the imine fragment can switch the stereoselectivity of the PPY-catalyzed Staudinger reaction. To do so, we employed the density functional theory (DFT) for geometry optimization and electronic analysis at the B3LYP/6-31G(d) level of theory to examine and compare the role of *N*-tosyl (*N*-Ts) and *N*-triflyl (*N*-Tf) imine on the mechanism pathways, *i.e.*, imine-first or ketene-first, and stereochemistry of the reaction, *i.e.*, *cis* or *trans* β-lactam. Our results show that the reaction mechanism pathway cannot be simply switched from ketene-first to imine-first by changing the substituent on the imine nitrogen atom, which is contrary to the reported experimental results, and both imines go through the ketene-first mechanism with different stereochemistries, which is *cis* selective for imine-Ts and *trans* selective for imine-Tf. Based on electronic analyses, the reversal in diastereoselectivity in the *N*-triflyl imine system could be attributed to the charge transfers and electron-density distribution over the transition states. Therefore, the *cis*/*trans* selectivity of the PPY-catalyzed Staudinger reaction could be effectively controlled by the electronic characteristics of the molecular substituents in the reactants. A N-protecting group in imine with a more electron-withdrawing nature seems to accelerate the stereo-determining step, ring closure, and increase the stabilization charge transfers in the transition state, leading to a preference for *trans* β-lactam formation. It seems that using a N-substituent with a higher electron-withdrawing nature can initially activate the imine by the nucleophilic catalyst in competition with ketene, *i.e.*, imine-first *versus* ketene-first. These results can provide an insight into select proper substituents for the fragments to synthesis β-lactam with a desired stereochemistry. Also, a comprehensive comparison was performed between calculations with and without dispersion.

## Abstract 2

This paper evaluates the electronic effects of molecular substituents on the stereoselectivity of the umpolung Staudinger catalytic reaction.

## Graphical abstract

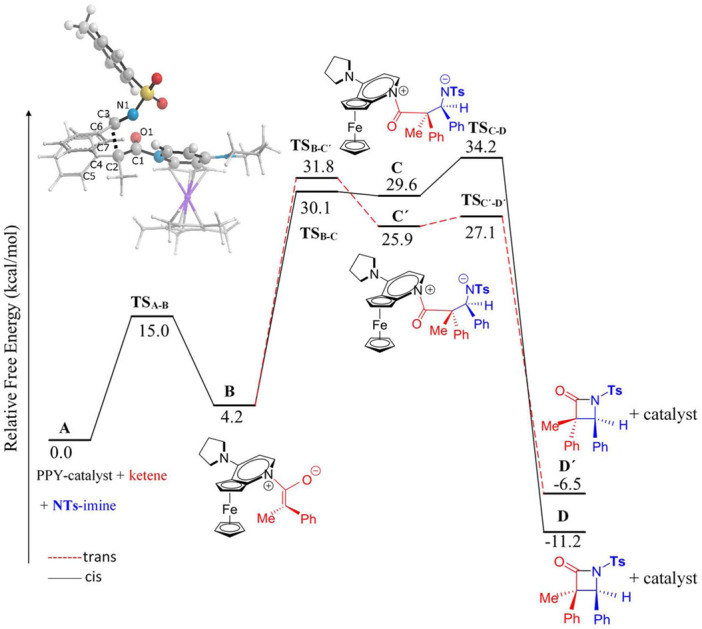

## Introduction

**Fig. 1:**
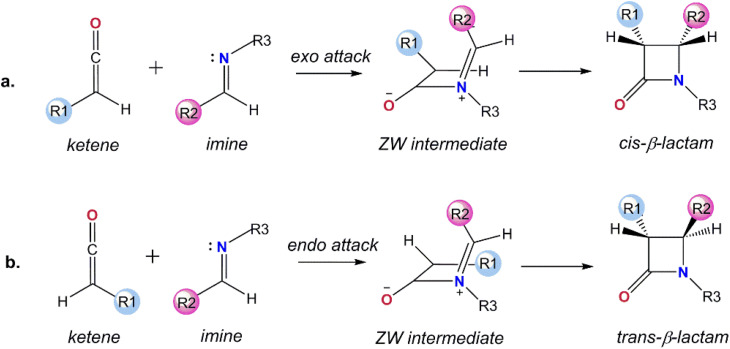
The mechanism for the Staudinger reaction: (a) *cis*, and (b) *trans* pathways.

β-Lactam antibiotics are the most popular family of medicines to treat bacterial infections.^1,2^ Therefore, synthesis of the β-lactam core, the key structural element in these antimicrobial agents, has received much attention over the years.^3–5^ Beside the bioactivity of β-lactams, their efficiency as precursors to the other families of compounds, including enantiopure amino acids and azetidines, make them interesting targets.^6–8^ Long before coming to the forefront of bioactivity and synthetic utility, Staudinger reported a cycloaddition reaction between a ketene and imine to produce the β-lactam scaffold (Fig. 1).^9,10^ The Staudinger reaction basically takes place in two steps: in the first step, a nucleophilic attack of the nitrogen of the imine on the central carbon of the ketene generates a zwitterionic (ZW) intermediate and the second step is the rate-determining and stereoselective step that involves a ring closure of the ZW intermediate through an intramolecular nucleophilic attack of the enolate on the imine moiety.^11^

The configuration of the substituents at the two chiral C centers in the β-lactam frame determines the stereochemistry of the final product, *cis*-, *trans*-, or a mixture of *cis*- and *trans*-β-lactam, which subsequently affects the bioactivity of the β-lactam derivatives.^12^ From a molecular point of view, the stereoselectivity of the asymmetric Staudinger reaction depends on a number of factors, including the spatial hindrance and electronic nature of the substituents attached to the end carbons of the ketene and imine. These are known features that determine the initial *endo* or *exo* approaches of imine to the ketene at the first step and direct ring closure or the isomerization of the imine segment in the ZW intermediate in the second step of the reaction mechanism. In spite of the considerable investigations performed focused on the impact of the substituents on the stereoselectivity of the Staudinger reaction, the pathway for the formation of the stereochemical outcomes has not yet been explained universally.^13–19^

Considering β-lactam's bioactivity as an antibiotic, the development of an efficient synthetic procedure to construct the β-lactam scaffold with the desired diastereochemistry is the most challenging task. Using chiral auxiliary groups attached to the imine, ketene, or both can control the substitution pattern at the chiral C sites of the β-lactam ring.^20–22^ Chiral auxiliary-based procedures are effective in cases where the auxiliary is a part of the target product, otherwise, this strategy requires additional steps to install and remove the auxiliary. Opposed to the chiral auxiliary, the application of chiral nucleophilic catalysts is extremely useful and presents a versatile method to introduce diastereoselectivity in the Staudinger cycloaddition reaction.^23,24^ As an example, transition metal catalysts have received significant attention during the last years to influence the configuration of the product ring by pairing with the reactants to form a compound with new characteristics, which can then undergo stereoselective reactions.^23,25–27^

Apart from what was mentioned above, in the case of the electron-deficient imine, the rate of nucleophilic attack on the ketene is minimal and the Staudinger reaction does not take place without a catalyst. Using a nucleophile or Lewis acid is necessary to make the cycloaddition reaction between ketenes and electron-deficient imines possible. The nucleophile can serve as an activation agent for the ketene and a Lewis acid can be employed to activate the less-nucleophilic imines. The first catalyzed Staudinger reaction between an electron-deficient imine and relatively stable ketene was reported by Lectka, who introduced transition metal salts as nucleophiles, activating the ketene to interact with the less-reactive imine.^25^ Lectka and co-workers extended the catalytic asymmetric synthesis of β-lactam using chiral nucleophilic amine catalysts, including benzoylquinine and cinchona alkaloid derivatives, which serve two discrete roles: one as the proper base to produce a monosubstituted ketene and another as a nucleophile catalyst to form β-lactam at the desired stereoselectivity, *i.e.*, *cis*-β-lactam.^23,24,28^ Although, using organocatalysts in the Staudinger reaction increased the enantioselectivity, the chemical yield of the reaction remained moderate, about 40–65%. Further efforts by Lectka's group continued to achieve high chemical yields and enantioselectivity by employing a bifunctional catalytic approach. In their suggested bifunctional Lewis acid-nucleophile based catalyst, a Lewis acid cooperates with a chiral nucleophile to promote the synthesis of *cis* β-lactams. They reasoned that an achiral Lewis acid is partly responsible for the additional activation of the imine.

In an effort to improve the enantioselectivity for β-lactam formation, Fu and co-workers proposed a planar-chiral nucleophile catalyst to complement the original study of Lectka.^29,30^ In their reported catalytic approach, they suggested the 4-(pyrrolidino) pyridine (PPY) derivative as an effective catalyst for the reaction of more stable disubstituted ketenes with a range of imines. In addition to a high chemical yield and enantioselectivity, good diastereomeric ratios were achieved in the case of unsymmetrical disubstituted ketenes. In these studies, the “ketene-first” mechanism was introduced as a favorable pathway for the catalyzed Staudinger reaction by a nucleophile.

Another group of studies has been dedicated to the umpolung (polarity inversion) Staudinger concept by using another type of chiral nucleophile as a catalyst, namely N-heterocyclic carbene (NHC).^31,32^ In these instances, the chemical yields and enantiomeric excess were evaluated as moderate to very good, whereas, the diastereomeric ratios appeared to be dependent on choosing the N-heterocyclic carbene catalysts. The Staudinger reaction catalyzed by an unsymmetrical NHCs furnished *cis* β-lactams, while the reaction catalyzed by a C2v symmetric (achiral) NHC, namely *N*,*N*′-bis(2,6-diisopropylphenyl)imidazole-2-ylidene, predominantly afforded *trans* β-lactams.^31^

Each method is interesting in its own right and as a group they show that the umpolung catalytic process should be taken seriously as a foundation for the nucleophile-catalyzed Staudinger reaction. While the formation of *cis* diastereomers has taken priority in most of these methods, there are relatively few studies that have concentrated on the synthesis of *trans* β-lactam isomers. In this regard, Fu and co-workers reported that the N-protecting group of the imine is effectively responsible for stereocontrol during the Staudinger cycloaddition catalyzed by PPY derivatives.^30^ The Staudinger reaction is *trans* selective in the case of coupling ketenes with *N*-triflyl (*N*-Tf) imines, whereas the process preferentially affords the *cis* isomer when employing *N*-tosyl (*N*-Ts) imines as reacting partners. Based on Fu's reasoning, the “imine-first” mechanism was introduced. Lectka also tested the construction of *trans* β-lactams based on the idea of designing an anionic nucleophilic catalyst with a bulky counterion.^33^

In general, the stereoselectivity in this category of reactions can be effectively controlled either by choosing a catalyst with superior nucleophilic characteristics ^24,27,33–35^ or by the N-protecting group of the imine.^30^ Filling gaps in the research reports regarding increasing the efficiency of the catalyzed Staudinger reaction as a synthetic tool for β-lactam is an immediate necessity. For this purpose, a detailed understanding of the reaction mechanism would be very important to progress applications by improving the catalytic reaction. Based on these observations, we focused on the catalyzed asymmetric Staudinger reaction that favors *trans* β-lactams, with an aim to find a superior procedure based on quantum mechanical rules. In this study, we performed a density functional theory (DFT) study of the Staudinger reaction mechanisms reported by Fu and co-workers.^30^

Therefore, we developed the first computational study addressing the catalytic *trans*-selective Staudinger reaction based on two previously reported mechanisms: ketene-first and imine-first mechanisms. To find out the key elements for the success of the process, it was necessary to ponder the electronic origin of the reversal in diastereoselectivity. A better understanding of the molecular mechanisms would allow researchers to design reactions with higher chemical yields of the target product with the desired stereochemistry.^36,37^ The quantum chemical approach is a powerful method for investigating catalytic reactions.^38,39^

## Models and computational detatils

Computations were carried out through a density functional theory (DFT) approach as implemented in the Gaussian 09 program.^40^ Becke's three parameters with Lee–Yang–Parr's (B3LYP) formulation of hybrid exchange approximation was used to optimize the gas-phase geometry of all the species.^41,42^ We employed an all-electron basis set, 6-31G (d), for the C, O, N, S, F, H atoms, and an effective core potential type basis set, LANL2DZ,^43^ for Fe. Frequency calculations were performed to ensure the reliability of the predicted stationary points as minima or transition states. The energy values reported in this study were zero-point and thermally-corrected free energies changes (Δ*G*) at 298 K. A scan technique was used to sample the potential energy surface (PES) for interactions and find points of interest on PES, including the minima and maxima corresponding to the equilibrium and transition structures. Although scan calculations provide insights into the PES, they do not define the lowest energy path between the reactants and products. To validate transition structures, we followed the obtained transition structure on a potential energy surface downhill to the reactants and to the products using intrinsic reaction coordinate (IRC) calculations. In this way, an IRC calculation connects the correct reactants and products on PES by a path which passes through the transition state between them.

Electrostatic potential (ESP) analysis was used to determine the effective factors in the stereoselectivity of β-lactam formation in the Staudinger reaction. The electrostatic potential was visualized as a mapped surface that provided useful information about the charge distribution over the reactant molecules. The electron density isosurface was colored according to the value of electrostatic potential at each point on it; regions of large positive and negative electrostatic potential conventionally appeared red and blue respectively within the ESP display.

The non-covalent interaction (NCI) technique was employed to locate and characterize the non-covalent interactions in the interacting complexes.^44^ Visual analysis of the electron density and its reduced gradient based on the NCI method can reveal the nature of the dominant interactions in the intermediates during the addition mechanism. Three-dimensional (3D) reduced density gradient (RDG) surfaces were considered to qualitatively explain the proper stereo-orientation of the ketene, amide, and catalyst toward each other and thus affecting the stability of the interacting complex. The dimensionless RDG(s) was determined based on the density, *ρ*, and its first derivative, ∇*ρ*:1
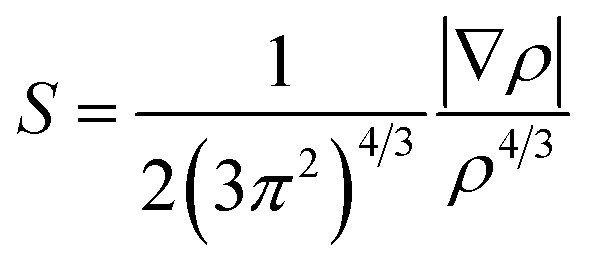


Graphs of the RDG surfaces were colored on a blue–green–red scale: red for repulsive, green for weak van der Waals, and blue for attractive interactions. RDG isosurfaces were visualized by the Visual Molecular Dynamics (VMD) program.^45^

Natural bond orbital (NBO) analysis was conducted to provide an explanation for the role of the imine N-protecting substituent in the charge-transfer interactions throughout the molecular complexes, and subsequently, in the β-lactam stereoselectivity.^46^ The most popular aspect of the NBO method is its ability to transform the delocalized molecular orbitals to their localized equivalents. Natural bond orbital analysis provides a convenient basis to study inter- and intramolecular bonding through investigation of the charge-transfer interactions in molecular complexes. The stabilization energy resulting from the donor NBO_*i*_ → acceptor NBO_*j*_ delocalization was evaluated by second-order perturbation theory,^47^2
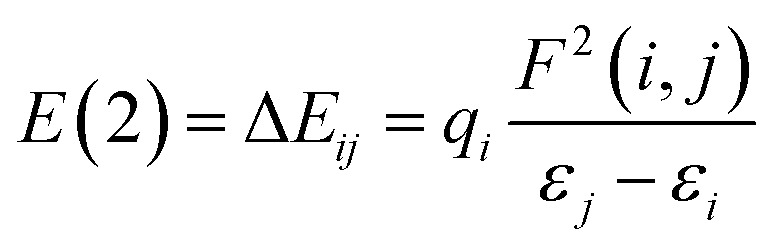
where *q*_*i*_ is the occupancy of the donor orbital, *ε*_*i*_ and *ε*_*j*_ are diagonal elements (orbital energies), and *F*(*i*,*j*) is the off diagonal element of the NBO Fock matrix. A larger *E*(2) value corresponds to a more intensive delocalization interaction and greater stabilization of the molecular complex.

## Results and discussions

**Fig. 2:**
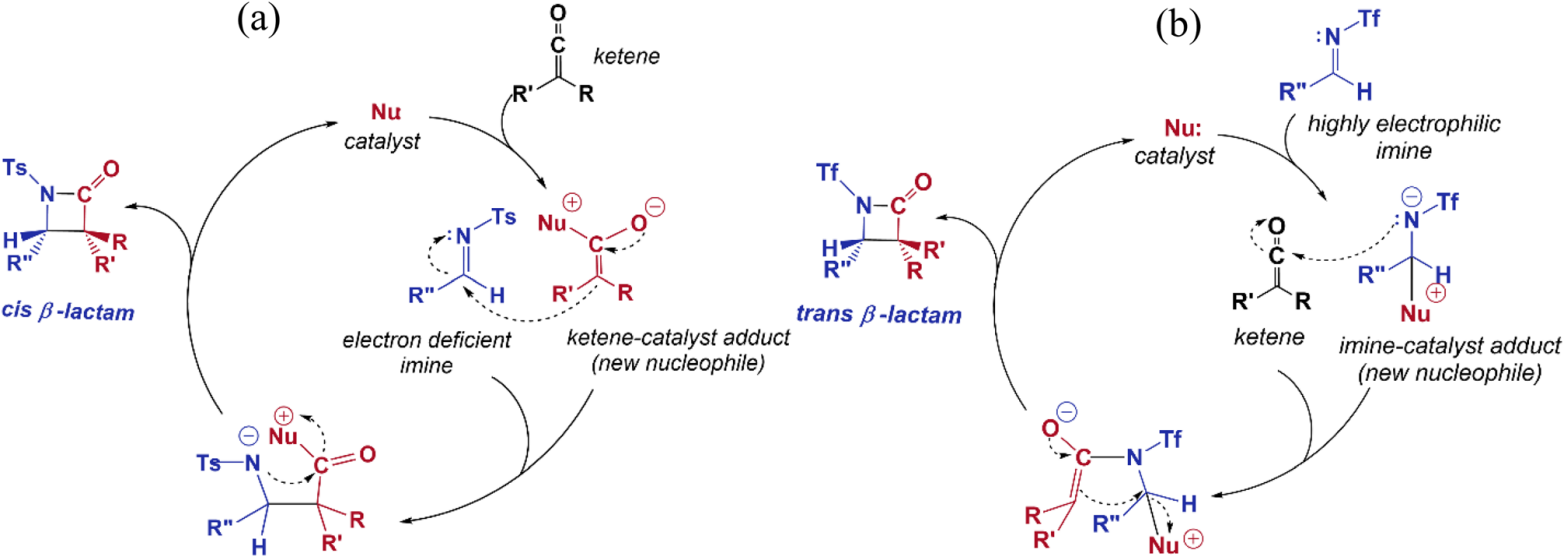
(a) “Ketene-first” and (b) “imine-first” mechanisms for the nucleophile-catalyzed reversed Staudinger reaction.

Imine electrophilicity necessitates an inversion of ketene's polarity, so, the classical Staudinger pathway is broken and restarted with a reversed mechanism, in which the ketene acts as a nucleophile toward the electrophile imine, *i.e.*, the “umpolung Staudinger approach”.^7^ The polarity conversion of ketene can be achieved *via* using a nucleophile catalyst that can attack at the central carbon of ketene to form a zwitterionic enolate. Subsequently, the ketene-catalyst adduct is able to participate in nucleophilic attack of the electron-deficient carbon of the imine. The nucleophile catalyst is then separated from the final complex to form the desired β-lactam product. The Staudinger reaction catalyzed by PPY thus proceeds through the “ketene-first” mechanism represented in Fig. 2a.

In this paper, we investigated the catalytic cycle and the electronic origins of the stereoselectivity in the Staudinger reaction. Our research was conducted by studying the switch in Staudinger selectivity based on Fu's methodology, involving changing the substituents over the nitrogen atom in the imine.^30^ In Fu's method, diastereoselectivity is directed to the favored stereoisomer through changing the substituents attached to the nitrogen atom of the imine instead of the groups over the imine carbon atom; whereby the *N*-Ts imine favors *cis* β-lactam and *N*-Tf imine predominantly generates *trans* β-lactam. Based on Fu's mechanistic supposition, the key point of the *trans*-selective Staudinger reaction is to intensify the electrophilicity of the imine by incorporation of an N-protecting group with a greater electron-withdrawing capacity, so that, the reaction will proceed through the imine-first pathway, Fig. 2b.

The imine and ketene substituents play a key role in determining the stereoselectivity of the Staudinger reaction. So, to elucidate the contribution of specific molecular substituents to the stereoselectivity in the catalyzed Staudinger reaction, we employed a DFT methodology to investigate the interaction between the ketene and imine without considering solvent and temperature effects. The catalytic asymmetric Staudinger reaction between a disubstituted ketene and imines with different nitrogen substituents is an appropriate reaction platform to assess the diastereoselectivity dependency on the N-protecting group of the imine.^30^ To answer whether the *N*-triflyl group of the imine increases the probability of reaction between the PPY catalyst and imine, rather than the initial formation of the ketene-enolate, we conducted a comprehensive study on catalyzed Staudinger reactions using both N-protecting groups in the imine, and triflyl and tosyl substituents.

**Fig. 3:**
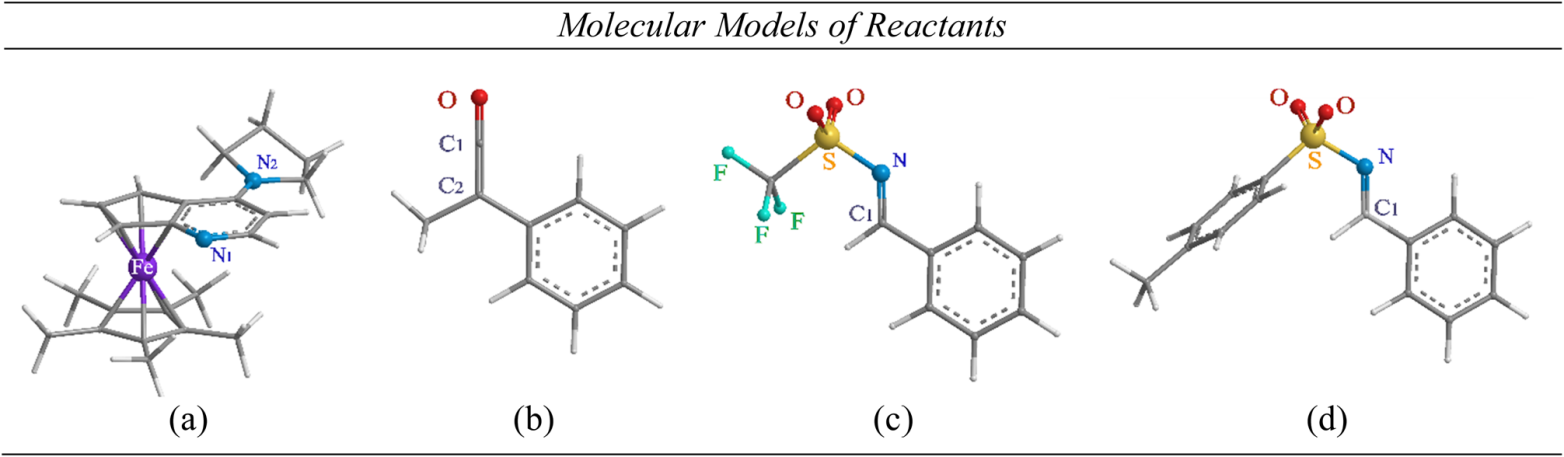
Molecular structures of the (a) PPY catalyst, (b) methyl phenyl ketene, (c) *N*-triflyl-, and (d) *N*-tosyl- substituted imines.

Our study started by selecting a model reaction, utilizing a planar-chiral nucleophile catalyst derived from 4-(pyrrolidino) pyridine (PPY), an unsymmetrical disubstituted ketene (methyl phenyl ketene), *N*-tosyl-, and *N*-triflyl-substituted imines as reactants, Fig. 3. The PPY-derivative catalyst was first effectively used by Fu and co-workers to produce β-lactams with satisfactory stereochemistry from the asymmetric Staudinger reaction between symmetrical or unsymmetrical disubstituted ketenes with a variety of imines.^30^ In the proposed catalyst, the transition metal is as an asymmetric center that does not take part in the catalytic reaction, taking a “spectator” role. The nitrogen in the pyridine (C_5_H_5_N) part of the PPY-derivative catalyst has a high electronegativity influence on the molecular system, which makes it a remarkable atom for taking part in a nucleophilic attack. The outward orientation of the lone pair orbital on nitrogen facilitates a strong overlap with an orbital on an electron-deficient atom in a substrate molecule. Accordingly, the first step of all the reaction pathways studied in this paper was conducted through the nucleophilic attack of *N*-pyridine in the PPY catalyst to the reactant (ketene or imine). In this study, our primary objective was to investigate how varying the N-protecting group in the imine fragment could influence the stereoselectivity of the PPY-catalyzed Staudinger reaction with a particular emphasis on (*E*)-imines and to understand the mechanistic details specific to (*E*)-imines. The choice to focus on (*E*)-imines was rooted in their prevalent reactivity and significance in many chemical transformations.^48,49^ It is, however, pertinent to highlight that under certain systems and conditions, imine isomerization can be an influential factor in the Staudinger reaction. A comprehensive study by Banik *et al.* underscored the role of isomerization steps prior to the formal (2 + 2) cycloadditions in specific Staudinger reactions.^50^

### Stereoselectivity of the nucleophile-catalyzed Staudinger reaction

The initial attack of the catalyst to the reactant can occur through *endo* or *exo* attack, leading to a zwitterionic intermediate. Here, we considered the more preferential *exo* attack of the PPY-derivative catalyst to the lower sterically hindered face of ketene, giving the *E* configuration of the zwitterionic enolate intermediate. The probable reaction between the (*E*)-zwitterionic enolate and two different reacting faces of the (*E*)-imine, Re or Si face, causes the stereoselectivity of the Staudinger reaction, favoring the *cis* or *trans* product, respectively. In similar fashion, the *exo* attack of the PPY catalyst to the (*E*)-imine generates a zwitterionic intermediate through the imine-first mechanism, which subsequently interacts with the ketene from the Re or Si face.

Unraveling the mechanism by which a ketene and an imine perform the Staudinger reaction in the presence of a catalyst requires evaluation of the energy profiles for all possible pathways, which entails a large number of energy calculations for all the intermediates and transition states. To understand the stereochemistry origin in depth and test the effect of the N-protecting group of imines on the reaction mechanism, we performed quantum mechanical analysis for the imine- and ketene-first pathways with both *cis*- and *trans*-stereoselectivity.

### Nucleophile-catalyzed Staudinger reaction between *N*-tosyl imine and ketene

**Fig. 4:**
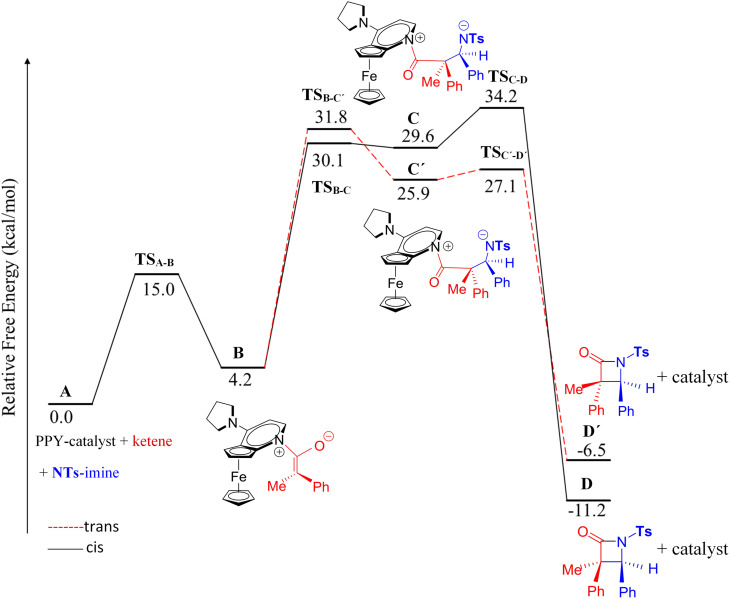
Gibbs free-energy profiles for the competing mechanisms of the cycloaddition catalyzed Staudinger reaction between ketene and NTs–imine.

In the case of the less-nucleophilic imine, we turned our attention to the reversed Staudinger reaction, where nucleophilic attack of the PPY catalyst first takes place at the carbon position of the ketene group. The free-energy profile for the ketene-first mechanism is shown in Fig. 4. The nucleophilic attack at C1 would disrupt the pi–pi interaction between the carbon and oxygen in the ketene, leading to the formation of an enolate species with a pi-OCC heteroallylic molecular orbital distribution, *i.e.*, intermediate B. We concurrently studied the possible side reactions in the formation of the zwitterionic *N*-tosyl imine through the imine-first attack of the catalyst. The results of these two calculations clearly indicated that the nucleophilic attack at C1 in the ketene is favored with a much lower energy barrier (about 16.2 kcal mol^−1^) compared nucleophilic attack at the carbon of the imine, which indicates that the ketene is more prone to be attacked by a nucleophilic catalyst through a ketene-first mechanism.

The zwitterionic ketene-enolate attack to the *N*-Ts imine installs the stereochemistry of the final product. To answer which conformation is favored though, both *cis* and *trans* pathways were studied here. The cycloaddition of the imine to the ketene occurs in a stepwise manner. In the first step, C–C bond formation between the imine and ketene through the transition states TS_B-C′_ and TS_B-C_ leads to the intermediate C′ and C for the *trans* and *cis* forms, respectively. This step passed the barrier of 27.6 kcal mol^−1^ for the *trans* conformation and 25.9 kcal mol^−1^ for the *cis* addition, and the processes were found to be endergonic by 21.7 and 25.4 kcal mol^−1^ relative to intermediate B, respectively. At the second step, intermediates C and C′ go through the ring-closure transition states TS_C-D_ and TS_C′-D′_ to form the four-membered ring of β-lactam. The overall barriers leading to the transition states TS_C-D_ (*cis*) and TS_C′-D′_ (*trans*) were 30.0 and 22.9 kcal mol^−1^ relative to B. *Cis* and *trans* β-lactam formations were exergonic by 11.2 and 6.5 kcal mol^−1^, relative to the isolated reactants, *i.e.*, A.

The free-energy profiles in Fig. 4 show that the *trans* diastereomer of β-lactam, *i.e.*, product D′, was thermodynamically less stable compared to the *cis* diastereomer, *i.e.*, product D. However, the energy barriers involved in the *trans* formation of β-lactam were lower than those in the case of *cis* β-lactam. Thus, the *cis* diastereomer could be expected to prevail when the Staudinger reaction is performed under thermodynamic control conditions, whereas the *trans* diastereomer would predominate when the Staudinger mechanism is conducted under kinetic control conditions. Considering that the most often used temperature for the Staudinger reaction is room temperature or below, an effective strategy to decrease the impact of the energy barrier on the rate of reaction would be to increase the reaction temperature, as described by the Arrhenius equation, reaction rate = *A*e^−*E*_a_/*RT*^. As a result, conducting the reaction at relatively high temperatures and giving an adequately long time can be the simplest way to obtain *cis* β-lactam as a thermodynamically controlled product, which is located at the global minimum in the Gibbs free energy. In this condition the kinetic control reaction might be reversible. In contrast, at lower temperatures, the kinetically controlled product, *trans* β-lactam, is inevitably formed faster, at which point the product probably would not have enough energy to undergo the reverse reaction. To increase the yield of the *trans* diastereomer, it is necessary to identify the rate-determining step and manipulate the interactions at the transition state therein, and to isolate the kinetic product as soon as it is formed. The attack of the zwitterionic ketene-enolate to the Si face of imine in the *trans* pathway is the rate-determining step.

### Nucleophile-catalyzed Staudinger reaction between *N*-triflyl imine and ketene

**Fig. 5:**
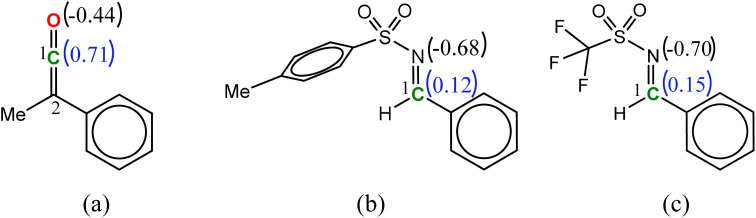
NBO charges for the reactants: (a) ketene, (b) imine *N*-Ts, and (c) imine *N*-Tf.

**Fig. 6:**
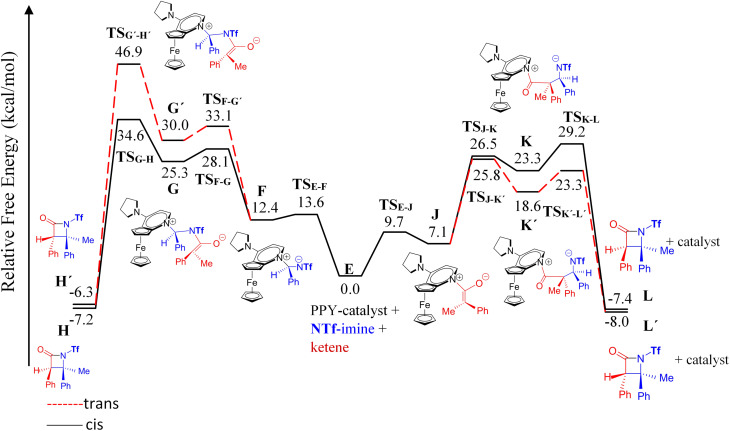
Free-energy profiles for two possible mechanisms, ketene-first (right side) and imine-first (left side), of the nucleophile-catalyzed Staudinger reaction involving *N*-Tf imine.

In the imine-first mechanism proposed by Fu *et al.* to explain obtaining the *trans* β-lactam product, they suggested that changing the functional group carrying the N in the imine from tosyl to triflyl makes a significant change in the electronic structure of the imine, so that it can be activated initially by the catalyst in competition with ketene. To identify whether the initial nucleophilic attack of the catalyst to the carbon of the ketene generating the PPY-ketene zwitterionic (J) intermediate has a lower energy barrier relative to the *N*-Tf imine activation by the catalyst, we performed NBO charge analysis on the ketene, and *N*-Tf and *N*-Ts imines, Fig. 5. As shown in this figure, the NBO charge on the C1s of *N*-Tf imine and *N*-Ts imine were in the same range (0.15 e and 0.12 e, respectively), and much lower than that for the C1 of ketene, 0.71 e. The replacement of the N-protecting substituent increased the electrophilicity of *N*-Tf imine compared to *N*-Ts imine and decreased the energy barrier for the interaction between the ketene-catalyst adduct and the imine. The free-energy results plotted in the right side of Fig. 6 (discussed in the following) support our justification that the *N*-Tf imine is more active compared to *N*-Ts in the ketene-first mechanism. For example, comparing the *trans* stereochemistry, *N*-Ts imine addition to the enolate occurred through a T_B-C′_ at 31.8 kcal mol^−1^, Fig. 4, while this step for *N*-Tf imine had a barrier of 25.8 kcal mol^−1^ (TS_J-K′_ in Fig. 6). Nevertheless, NBO charge analysis indicated that the stronger electrophilicity of C1 in the ketene relative to in our considered imines facilitated the catalyst nucleophilic attack on the carbon of the ketene. Therefore, the charge increment at the center of the nucleophilic attack, *i.e.*, at C1, from *N*-Ts imine to *N*-Tf imine was not enough to make the imine the first target for the catalyst. Accordingly, it could be expected that the ketene-first mechanism for the *N*-Tf imine is more energetically preferential.

In an attempt to comprehend the reason for the *trans* selectivity under different experimental conditions and *N*-triflyl imine as a reactant, we evaluated the overall free-energy profiles for all possible pathways, *i.e.*, for the ketene- and imine-first mechanisms for both *cis* and *trans* stereoselectivity, and the results are plotted in Fig. 6. With the side-by-side comparison of the free-energy profiles, it could be found that the switching mechanism from ketene-first to imine-first for *N*-Tf imine significantly shifted up the overall energy level. The overall energy barrier of the imine-first pathway reached 46.9 and 34.6 kcal mol^−1^ for *trans* and *cis* stereoselectivity, respectively. Furthermore, the nucleophilic attach of the catalyst to the imine proceeded through a 13.6 kcal mol^−1^ barrier, 3.9 kcal mol^−1^ higher than the corresponding step for the ketene-first pattern. These results suggest that the imine-first mechanism is hardly feasible (or even unfeasible), and the ketene-first mechanism is preferable to the imine-first for *N*-Tf imine, consistent with the reported results for the Staudinger reaction catalyzed by *N*-heterocyclic carbine.^51^ As a supporting note, the ketene-first mechanism is an umpolung catalyzed Staudinger reaction, which was successfully introduced by Lectka *et al.*^23,33,34^ However, different observations are expected depending on the experiment conditions due to competition between the initial interaction of the ketene or imine with the catalyst.

As shown in the ketene-first energy profiles (Fig. 6), the ring-closure step with the highest energy barrier for the *cis* pathway, 29.2 kcal mol^−1^, is the rate-determining step; while for the *trans* cycloaddition pathway, the imine coupling with the catalyst-ketene adduct is the rate-determining step at 25.8 kcal mol^−1^. Obviously, the *trans*-diastereoselective reaction in the case of the ketene-first reaction involving *N*-Tf imine has a lower free-energy barrier compared to the *cis*-selective pathway, indicating that the triflyl N-protecting group in the imine reactant makes the formation of *trans* β-lactam more likely. In this mechanism, *cis* β-lactam is thermodynamically 0.6 kcal mol^−1^ less stable relative to the *trans* product.

Fu *et al.* performed experiments for the catalyzed interaction between *N*-Tf imine and ketene under different conditions with respect to the *N*-Ts imine to examine the possibility of the imine-first path occurring. The mixture of *N*-Tf imine and the catalyst was cooled to get the imine-catalyst adduct, see eqn (3). At low temperatures, there was a suppression of the reverse reaction (with 1.2 kcal mol^−1^ energy barrier). Nevertheless, the activation of *N*-Tf imine by the catalyst was not guaranteed in the presence of ketenes in the reaction environment. It may thus be necessary to use substituents with stronger electron-withdrawing characteristics as an N-protecting group to competitively increase the electrophilicity of the imine.3
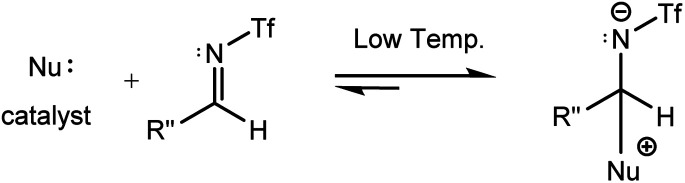


#### Pathway comparison

On the basis of energy considerations, we concluded that the ketene-first mechanism is a feasible pathway for the PPY-catalyzed Staudinger reaction. Thus, the divergent stereoselectivity could not be explained by the different mechanisms. Changes in the electronic structure of the starting imines led to different stereoselectivites through a unique mechanism. So, it is unequivocal that the crucial factors determining the relative *cis*/*trans* stereoselectivity through the β-lactam reaction is the electronic nature of the ketene and imine substituents. However, the experimental parameters, such as the temperature, interfere with the substituent's effects.

In the ketene-first mechanism for the reaction between *N*-Tf imine and the ketene, the formation of *trans* β-lactam was energetically favored over the *cis* β-lactam formation, which was in good agreement with the experimentally observed diastereoselectivity.^30^ The formation of *trans* β-lactam increased as the size of the N-protecting group of the imine decreased and its electron-withdrawing nature increased. The electronic effects of the N-substituent of the imine that obviously reversed the stereochemical outcome are discussed in the following.

### Discovering the origin of the stereoselectivity using electronic analysis

As can be seen in Fig. 4, the transition state of TS_C′-D′_ was 7.1 kcal mol^−1^ lower than the transition state TS_C-D_. Once the ring closure took place and the β-lactam molecule was separated from the catalyst, the relative stability became the opposite. In contrast to the trend observed for the mentioned TSs, the optimized system of *cis* β-lactam and the catalyst was found to be more stable than the *trans* product system by 4.7 kcal mol^−1^. This aligned with the stabilization donor–acceptor interactions in the transition states predicted by NBO analysis, as discussed in the following section.

**Fig. 7:**
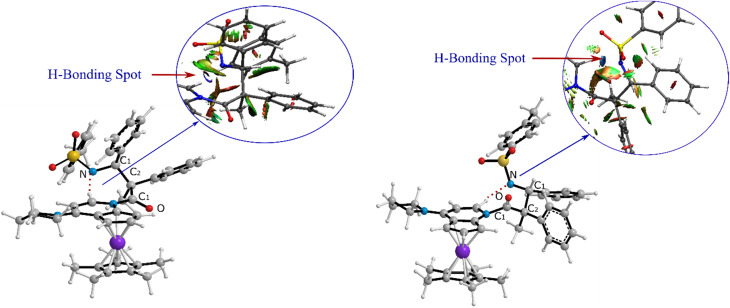
Non-covalent interaction analysis for (a) TS_C-D_ and (b) TS_C′-D′_ intermediates.

Visual analysis of the electron density and its reduced gradient were performed to reveal the nature of the dominant non-covalent interactions in the TS_C-D_ and TS_C′-D′_ transition states. The RDG 3D-gradient-isosurfaces presented in Fig. 7 could qualitatively explain how the different orientations of the imine toward the ketene-catalyst adduct affects the stability of the transition state. As inferred from Fig. 7, the presence of blue pill-shaped surfaces in the BGR coloring scheme was indicative of the presence of effective hydrogen bonding between the imine nitrogen and the hydrogen of the catalyst in TS_C′-D′_, leading to the *trans* diastereomer, and resulting in a higher stability for TS_C′-D′_ compared with TS_C-D_. The reason for this is that, in TS_C′-D′_, the N⋯H distance is shorter than that for the *cis* transition state (2.22 *vs.* 2.38 A). The significant difference between these two TSs appeared to be the nature of the transition states resulting from the different reacting faces of the imine, Re or Si. In the *trans* pathway, the imine takes the Re face in the reaction, in which the *N*-tosyl group is farther away from the catalyst moiety, leading to less steric hindrance in its transition state. A comparison of the rupturing and forming bond distances showed that the ring-closure transition state for the *trans* form, *i.e.*, TS_C′-D′_, resembles the product state, whereas the TS_C-D_ for the *cis* path resembles the previous intermediate state more than the next one. Therefore, it can be inferred that there is a “late” (or “early”) transition state for the *trans* (or *cis*) pathway. Increasing the intramolecular distance between the imine–ketene adduct and catalyst in the late TS_C′-D′_ has reduction effects on the main stabilization interactions between the donor and acceptor orbitals of the two fragments. So, the late transition state explains why the *trans* product was not thermodynamically favored. To see if local orbital interactions play a determining role in the stereoselectivity, we performed natural bond orbital (NBO) analysis in the following sections.

#### Electrostatic potential (ESP)

**Fig. 8:**
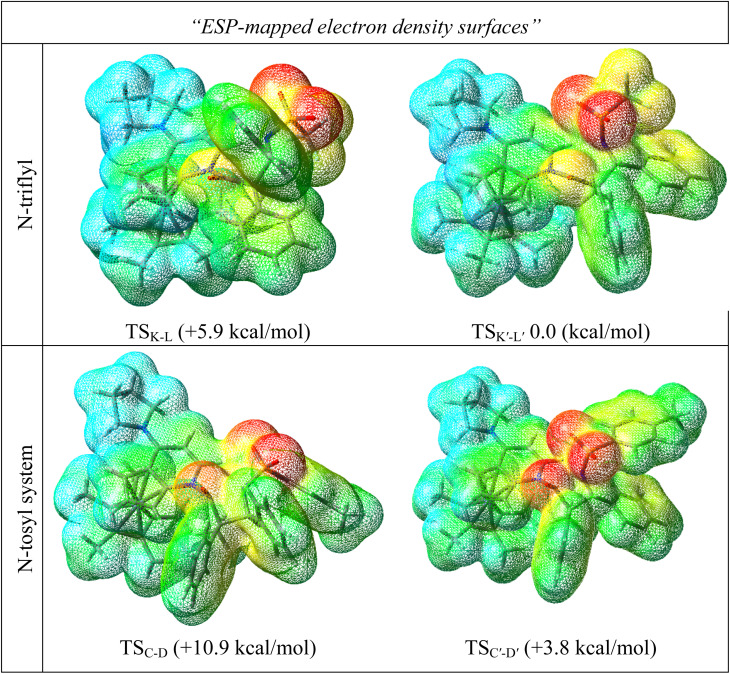
Optimized ring-closure transition states and corresponding ESP-mapped electron-density surfaces of the catalyzed ketene-first mechanism for *N*-Tf and *N*-Ts imine. Relative free energies (kcal mol^−1^) taken from the energy results reported in Fig. 4 and 6.

Electrostatic repulsion and steric hindrance are two main responsible factors related to the N-protecting group in the imine that should be considered when determining which is the more favorable stereochemistry. The comparison of the ESP-mapped electron density surfaces for both *N*-Tf and *N*-Ts imines presented in Fig. 8 shows that replacing *N*-tosyl with the *N*-triflyl group drew electron density out of interacting spot and decreased the electron density on the carbonyl group. This change in the electronic distribution resulted in 3.8 kcal mol^−1^ more stability in the *N*-Tf transition state, affording the *trans* diastereomer compared to the corresponding TS for *N*-triflyl imine. So, the stronger electron-withdrawing nature for *N*-Tf could diminish the role of steric repulsion in determining the more stable configuration, yet instead, the steric hindrance became bolder. Accordingly, the attack of the zwitterionic enolate to the Si face of *N*-Tf imine, favoring *trans* stereochemistry, was more energetically favorable than the transition state for the *cis* form. It could be concluded that N-protecting groups that increase the electrophilicity of the imine may control the stereoselectivity of the reaction predominantly by steric effects. As can be seen, steric hindrance overcame the electrostatic repulsions for the imine carrying a bulky N-substituent and the enolate addition to the Re face of the imine was more energetically preferential in transition state. Although, it is worth noting that possible stabilization charge transfers through transition state complexes may determine the stereochemistry of the final products. As a result, the electronically-favored and sterically-unfavored transition state for the *N*-Ts imine system, *i.e.*, TS_C-D_, may compensate for its instability through donor–acceptor interactions, affording the *cis* lactam. To verify this hypothesis, second-order perturbative interactions for the above-discussed transition states were analyzed.

#### Natural bond orbital (NBO) analysis

We first studied the second-order perturbative interactions for the corresponding transition states produced at the stereo-determining steps through the imine-Ts reaction, TS_B-C_, and TS_B-C′_ in Fig. 4, to address the question of why the *trans* product is kinetically favored while the *cis* diastereomer is the thermodynamically favored product. Listed in Table 1 are the second-order perturbation energies in the TS_B-C_ and TS_B-C′_ transition states. As shown in this table, the major stabilizations in TS_B-C′_ came from the lone pair orbital of N1 of imine-Ts to the σ* orbital of the C2–C3 bond formed between the imine and ketene (*E*_n → σ^∗^_ = 79.2 kcal mol^−1^) and charge transfer from the σ orbital of C2–C3 to the π* of the C1=O1 bond in the ketene (*E*_σ → π^∗^_ = 37.2 kcal mol^−1^). Further, there were other considerable charge transfers in the transition state related to the *trans* pathway, TS_B-C′_, like the interactions of C2–C3 σ and the σ* orbital with the C4–C5 π* orbital (*E*_σ/σ^∗^ → π^∗^_ = 14.3 or 22.4 kcal mol^−1^, respectively), and charge transfer from the σ* of C2–C3 to the π* of C6–C7 with *E*_σ^∗^ → π^∗^_ = 39.2 kcal mol^−1^, as well as the delocalization between π of C6–C7 and σ* of C2–C3 with *E*_π → σ^∗^_ = 13.5 kcal mol^−1^ stabilization. By comparison, the main corresponding interactions in TS_B-C_ resulted in lower stabilization compared to the corresponding interactions in the *trans* TS as explained above; including the interaction between the lone pair orbital of N1 and the σ* of C2–C3 between the imine and ketene (*E*_n → σ^∗^_ = 30.6 kcal mol^−1^) and the interaction between the σ of C2–C3 and the π* of C1=O1 in the ketene (*E*_σ → π^∗^_ = 20.6 kcal mol^−1^). Taken together, the stabilizing interactions through the *trans* configuration of the transition state, TS_B-C′_, lead to a more stable zwitterionic ketene-enolate, C′, and effectively lower the energy barrier for the next ring-closure step, Fig. 4. However, the overall energy analysis demonstrated that the reaction through *cis* stereoselectivity was more thermodynamically favorable, affording more stable β-lactam. It is reasonable that the stabilization donor–acceptor interactions in the transition states for the last step, *i.e.*, the ring closure, play an important role in determining the stereochemistry of the final product. With this in mind, we also analyzed the second-order perturbative interactions in TS_C-D_ and TS_C′-D′_. The ring-closure step occurred with the synchronous shortening distance between N of the imine and C of the ketene, leading to N–C bond formation, and a lengthening of the intermolecular bonds between the ketene and catalyst. Therefore, besides the charge transfers helping N–C bond formation, those facilitating bond breakage between the β-lactam moiety and catalyst are of special importance. Comparing the second-order perturbation energies for TS_C-D_ and TS_C′-D′_, it was demonstrated that there were significant donor–acceptor interactions from both the above-mentioned categories within the transition state for the *cis* configuration, TS_C-D,_ as shown in Table 1. Although, both TS_C-D_ and TS_C′-D′_ tended to significantly stabilize the interacting complex through charge-transfer interactions, like those from nitrogen of the imine to the carbon of the carbonyl group in the ketene part, affording the four-membered β-lactam ring, the *cis* product was more thermodynamically favorable by about 5 kcal mol^−1^, Fig. 4. This could be due to the major contribution of the charge transfers in TS_C-D_ from the nitrogen of the imine to the carbon of the ketene to close the lactam ring, 
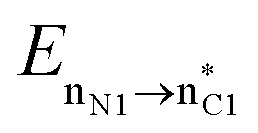
 = 125.6, 32.1, and 12.0 kcal mol^−1^, and the charge transfers from the ketene-imine adduct to the catalyst, 
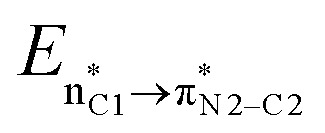
 = 97.8 and 472.8 kcal mol^−1^, causing β-lactam to detach from the catalyst (Table 1); while electron delocalization between the imine–ketene and catalyst in TS_C′-D′_ could not generate such great stabilization energies. To sum up, it is clear that the stereo-electronic effect in the *cis* pathway is more favorable than that in the *trans* pathway, confirming the reaction between NTs–imine and the ketene would preferentially furnish the *cis* diastereomer.

**Table 1:** Donor–acceptor interactions and their second-order perturbation energies (kcal mol^−1^) for the TS_B-C′_, TS_B-C_, and TS_C-D_ transition states

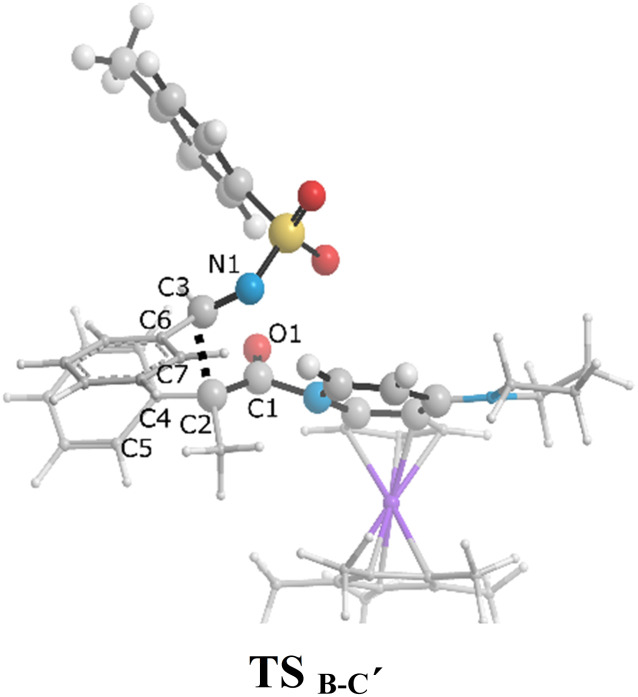		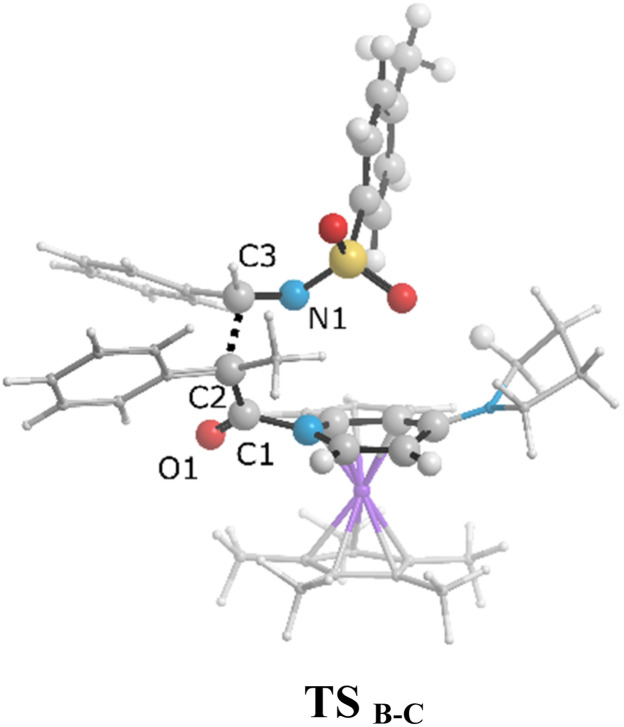		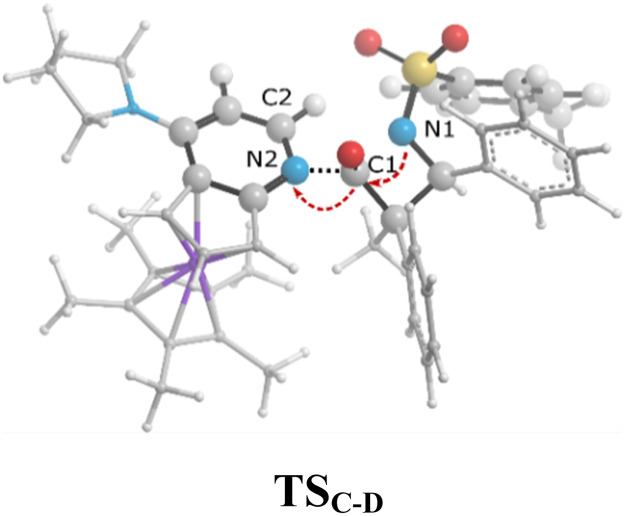	
TS_B-C′_		TS_B-C_		TS_C-D_	
Interactions	*E*(2)	Interactions	*E*(2)	Interactions	*E*(2)
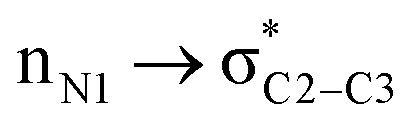	79.2	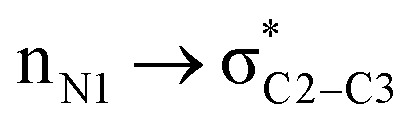	30.6	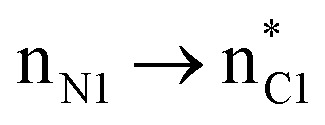	125.6
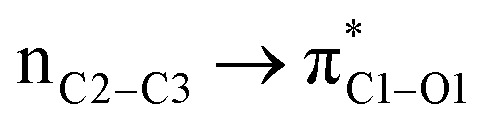	37.2	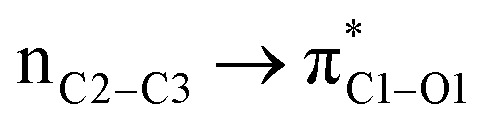	20.6	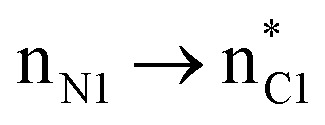	32.1
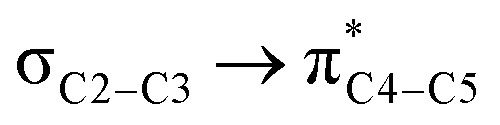	14.3			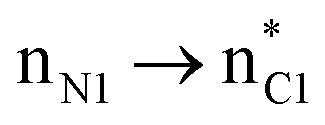	12.0
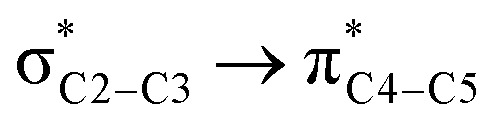	22.4			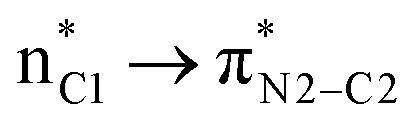	472.8
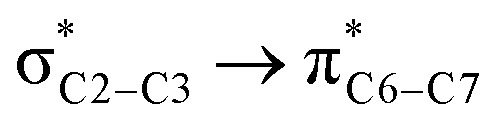	39.2			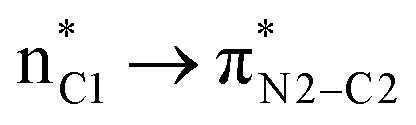	97.8
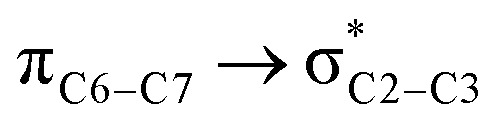	13.5				

In the case of the ketene-first mechanism for *N*-Tf, the second-order perturbation energies for the ring-closure step of the *cis* and *trans* pathways, TS_K-L_ and TS_K′-L′_, are listed in Table 2. The main donor–acceptor interactions in these transition states belonged to two categories: charge transfers responsible for closing the β-lactam cycle and charge transfers effective for the detachment of β-lactam from the catalyst. Comparing the *E*(2) values shows that the major charge transfers came from the charge transfers from the N1 of the imine or the O1 of the ketene to the vacant lone pair orbital C1 in both the *cis* and *trans* transition states. However, the resulting stabilization energies in TS, affording the *trans* stereochemistry, were higher than the corresponding values in the *cis* transition state; *E*_n → n^∗^_ = 261.2 and 275.8 kcal mol^−1^*vs.* 200.8 and 256.9 kcal mol^−1^, respectively. Besides, other charge-transfer interactions occurred from the nitrogen of the catalyst or oxygen of the ketene group to the σ-antibonding orbital of the N2–C1 bond between the catalyst moiety and the ketene-imine adduct, 
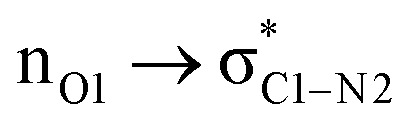
. and 
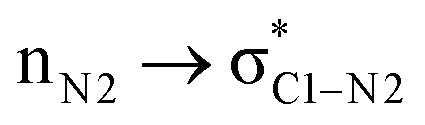
. These interactions helped rupture the connecting bond between the catalyst and the reactants, resulting in greater stabilization for the *trans* configuration compared to the *cis* one.

**Table 2:** Donor–acceptor interactions and their second-order perturbation energies (kcal mol^−1^) for the TS_K-L_ (left) and TS_K′-L′_ (right) transition states

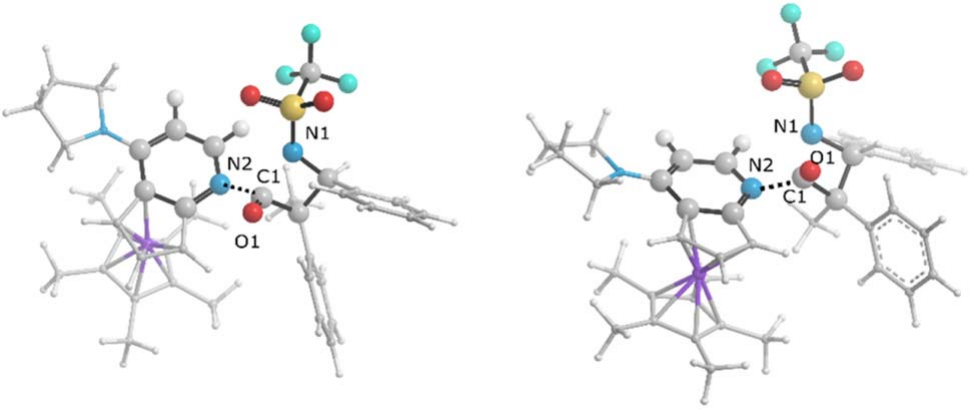		
*E*(2)	Interactions	*E*(2)
200.9	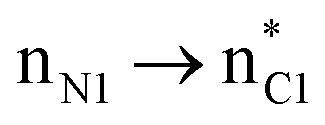	261.2
256.9	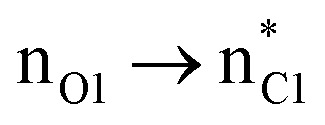	275.8
26.5	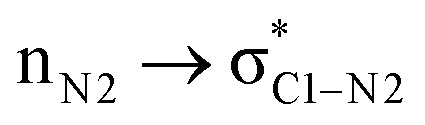	34.2
49.5	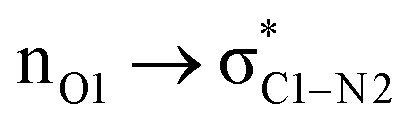	50.5

#### Dispersion corrections and solvent effect

**Fig. 9:**
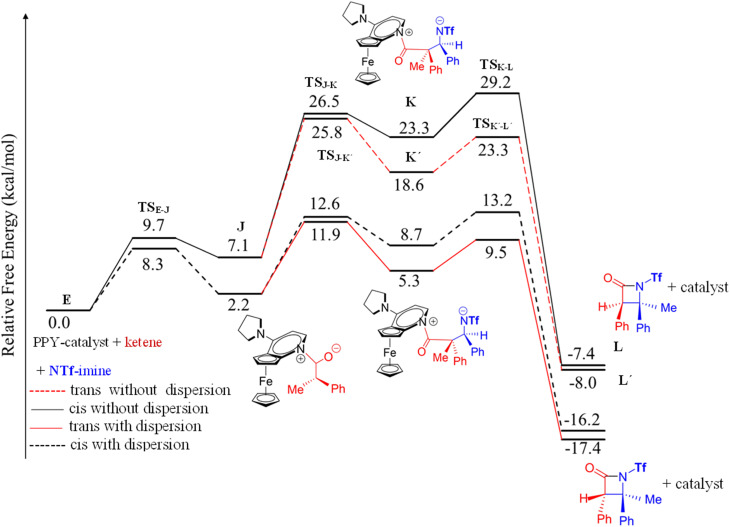
The Gibbs free-energy profile for the ketene-first mechanism with and without dispersion corrections; comparing the *cis* and *trans* selectivity of *N*-Tf imine.

To offer a thorough comparison between the calculations incorporating dispersion and those excluding it, we performed additional calculations for all the examined pathways. The outcomes are compared with those of systems lacking dispersion in Fig. 9, as well as in ESI Fig. S1 and S2,† which illustrate the Gibbs free-energy profiles. Despite the lower energies obtained in the calculations with dispersion corrections compared to those without dispersion, our results consistently confirmed the stereoselectivity observed in our original findings. For instance, the *trans* selectivity remained more favorable than the *cis* selectivity in the ketene-first mechanism involving *N*-Tf imine. This convergence between the calculations with and without dispersion corrections significantly strengthens the reliability and validity of our initial findings.

Furthermore, we noted an important observation that supports the reliability of the calculations without dispersion: the Gibbs free-energy profiles consistently exhibited a similar shape and pattern. This consistency implies that the relative energies and barriers between the different intermediates and transition states in the reaction pathway were in close agreement, indicating the qualitative aspects of the reaction mechanism were accurately represented. Furthermore, the comparison for systems accounting for solvent effects is illustrated in Fig. S3.† The congruence in the shapes and patterns provides additional substantiation of the robustness of our initial findings, and lends further support to the conclusion that the computational accuracy in the solvent phase aptly reflects both the *cis* and *trans* selectivity, as observed in the gas phase.

## Conclusion

In the present study, molecular and electronic factors affecting the mechanism and stereoselectivity of a catalytic Staudinger reaction were studied by means of DFT calculations. Quantum-based computations were performed on the Staudinger reaction between a disubstituted ketene and two different imines, *N*-tosyl and *N*-triflyl imines, by applying a PPY-derivative nucleophile catalyst, similar to the system investigated by Fu *et al.*^30^ They suggested a switching mechanism from ketene-first to imine-first by changing the N-protecting group for the imine in the catalyzed Staudinger reaction, and subsequently, a reversal in diastereoselectivity favoring the *trans* stereochemistry for β-lactam as a result of altering the preference in the mechanism.

Observations for the free-energy profiles were in agreement with the stereochemistry for the products based on the experimental findings, determining the *cis* β-lactam as the predominant product for the catalyzed Staudinger interaction between *N*-tosyl imine and the ketene through the ketene-first mechanism. While, the energy results with the *N*-triflyl-substituted imine exhibited a reversal in β-lactam diastereoselectivity, favoring the *trans* stereochemistry. However, the main purpose of our research was to provide a mechanistic explanation for the *trans* diastereoselectivity in the case of *N*-triflyl imine. In accordance, precise electronic analysis was employed to address the question of whether switching from the ketene-first to imine-first mechanism for *N*-triflyl imine was the main reason for the reversal in diastereoselectivity (as suggested by Fu *et al.*). We believe charge distribution and the nature of the donor–acceptor interactions over the transition states for the ring-closure step were the essence for understanding the origin of the stereocontrol for the catalyzed Staudinger reaction.

The stereochemical outcomes (*cis* or *trans*) could be mostly determined and explained by the electronic effects of the imine N-protecting group in both approaching possibilities. Changing the N-substituent imine from tosyl to triflyl increased the electrophilicity of the imine, but not to the extent that it could compete with the ketene to interact with the nucleophilic catalyst to initiate the imine-first mechanism. While a change in the imine electrophilicity lowered the overall barriers for the catalyzed interaction between *N*-Tf imine and the ketene through ketene-first mechanisms compared to the corresponding pathways in the case of *N*-Ts imine. Mechanistically, the distinct stereochemical preference of the *trans* diastereomer for the *N*-Tf imine system could be expected to be achieved by the Staudinger reaction operated in the ketene-first pathway, which is the methodology based on the concept of umpolung catalysis (polarity inversion) originally proposed by Lectka *et al.*^23,33,34^

NBO analysis revealed that the *N*-triflyl group increased the feasibility of the Staudinger reaction by increasing the stabilization of the interacting complex through donor–acceptor interactions. Further electron delocalization analysis on the transition states of the stereo-determining step, *i.e.*, β-lactam ring closure, revealed that effective charge transfers with higher second-order perturbation energies determined the observed *trans* stereochemistry for the *N*-Tf system. Moreover, steric repulsion and steric exclusion were two possible contributing factors that influenced the reaction patterns. A comparison of the ESP-mapped electron-density surfaces for the transition states indicated that a bulky N-substituent in the imine caused the steric exclusion to overwhelm that of repulsion, and therefore, the transition state for the reaction pattern with lower steric hindrance was preferred.

It is worth nothing that the electronic effects interfered with the experimental factors, such as the solvent and temperature affecting the stereochemical outcomes. For example, our free-energy results were in accord with the idea that the ketene-first mechanism for the Staudinger reaction in the case of *N*-tosyl imine could be under thermodynamic control at high temperature, furnishing *cis* stereochemistry, whereas kinetic control governed the stereochemical outcome (*trans* diastereomer) at lower temperature.

Considering the size and complexity of the systems studied in our research, the inclusion of dispersion corrections would have posed substantial computational challenges and extended the computational time required to obtain meaningful results. However, we performed additional calculations specifically focusing on the ketene-first pathway for the *N*-Tf imine; this time including dispersion corrections. By doing so, we sought to address any concerns related to the omission of dispersion in our initial calculations and provide a more comprehensive analysis of the system.

In summary, as our computational results clearly showed, the role of the N-protecting group in the imine fragment was not significant in controlling the reaction mechanism, while it was important to control the stereochemistry. The outcome of the study provides experimentalist and mechanistic insights into the design of switchable catalyzed Staudinger reactions with high efficacy for producing the desirable β-lactam while ensuring that the reaction yield is not compromised. This study provides an in-depth understanding of the correlation between the electronic structure of the reactants and stereochemistry of Staudinger reactions.

## Author contributions

FP and SSM are co-first authors.

## Conflicts of interest

There are no conflicts to declare.

## Supplementary Material

RA-013-D3RA05286A-s001
