# Supplementary material for: Electronic origins of the stereochemistry in β-lactam formed through the Staudinger reaction catalyzed by a nucleophile
Source: RSC Adv. 2023 Nov 16;13(48):33654–67. doi: 10.1039/d3ra05286a (PMC10653035; doi:10.1039/d3ra05286a)
Supplement: RA-013-D3RA05286A-s001 [file RA-013-D3RA05286A-s001.pdf]

Electronic Supplementary Material (ESI) for RSC Advances.
This journal is © The Royal Society of Chemistry 2023

Electronic Supplementary Information

Electronic Origins of Stereochemistry in β-Lactam Formation through Staudinger Reaction Catalyzed by a Nucleophile

Farideh Pahlavan, Sedigheh Sadat Moosavi, Amin Reza Zolghadr,* Nasser Iranpoor*

Department of Chemistry, Shiraz University, Shiraz, 71946-84795, Iran

*Corresponding author: arzolghadr@shirazu.ac.ir; Tel: +98 713 613 7157, Fax: +98 713 646 0788, ORCID: 0000-0002-6289-3794 (A.R.Z), Nasser Iranpoor, E-mail: iranpour@shirazu.ac.ir

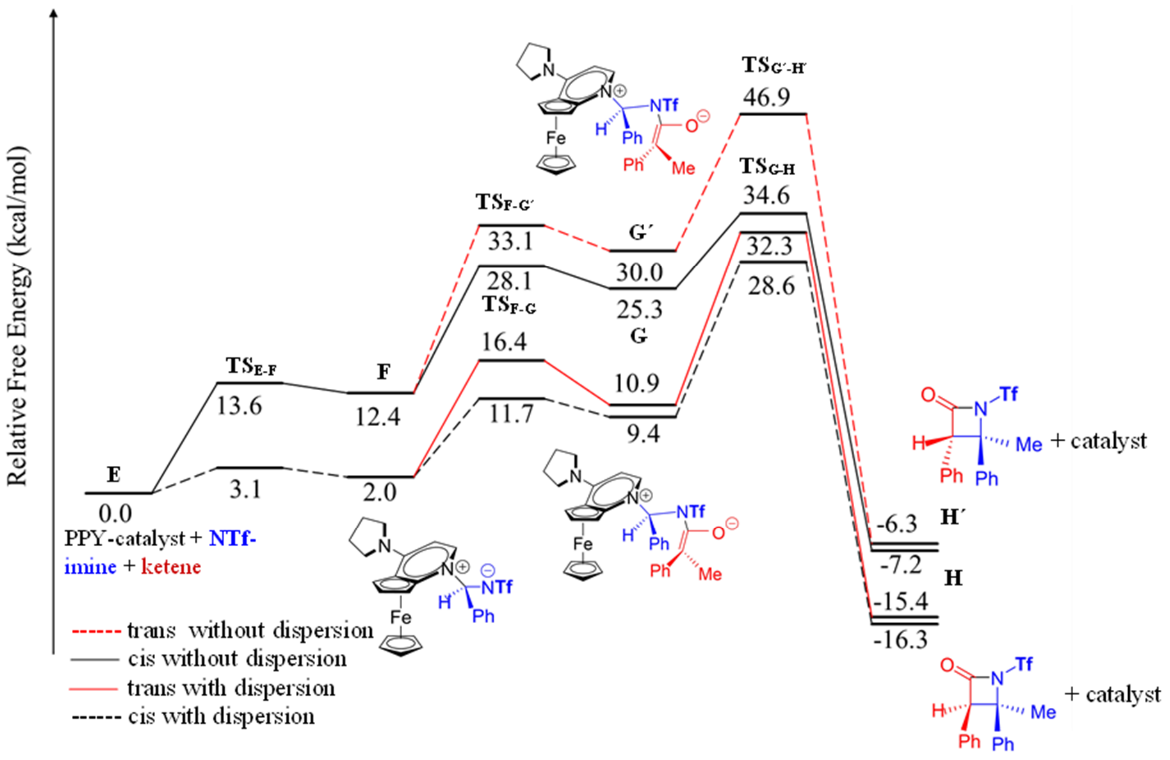

Fig. S1 Gibbs free energy profile for the imine-first mechanism with and without dispersion corrections, comparing cis and trans selectivity of N-Tf imine.

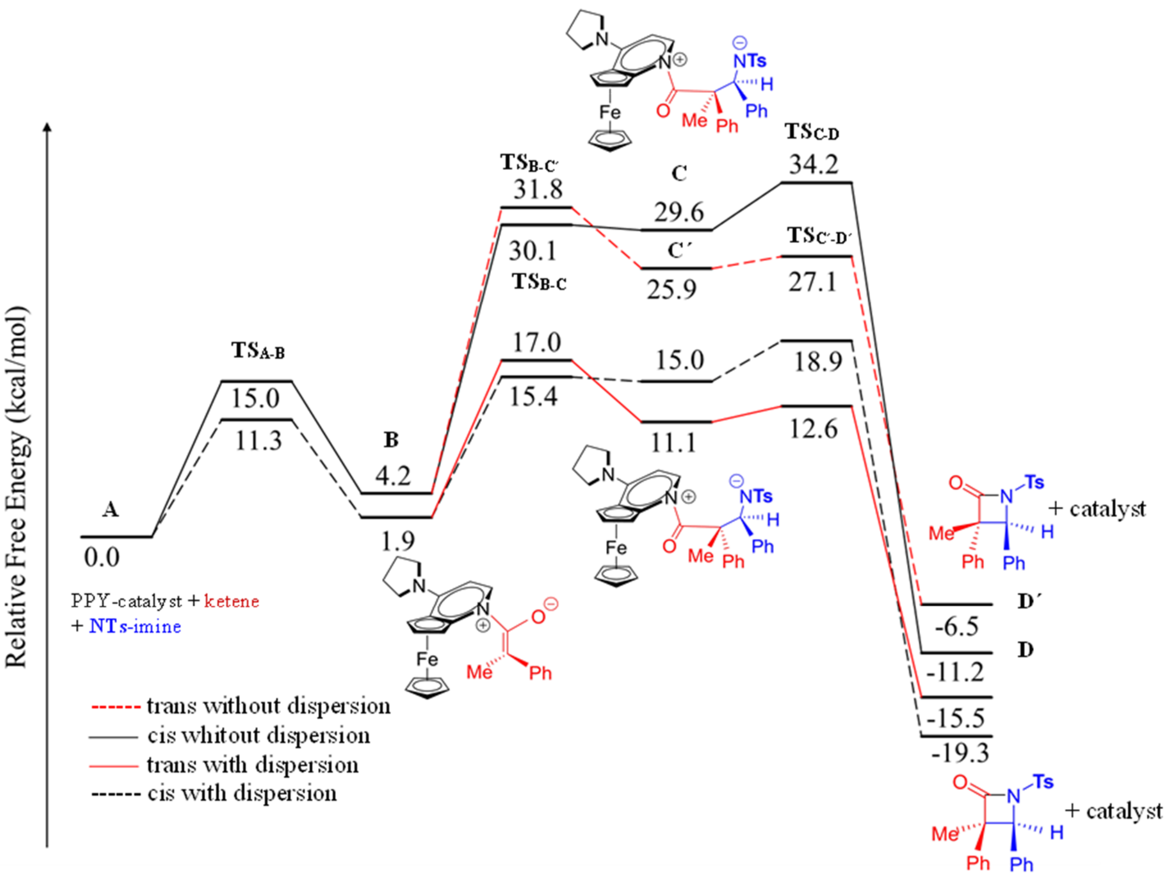

Fig. S2 Gibbs free energy profile for the ketene-first mechanism with and without dispersion corrections, comparing cis and trans selectivity of N-Ts imine.

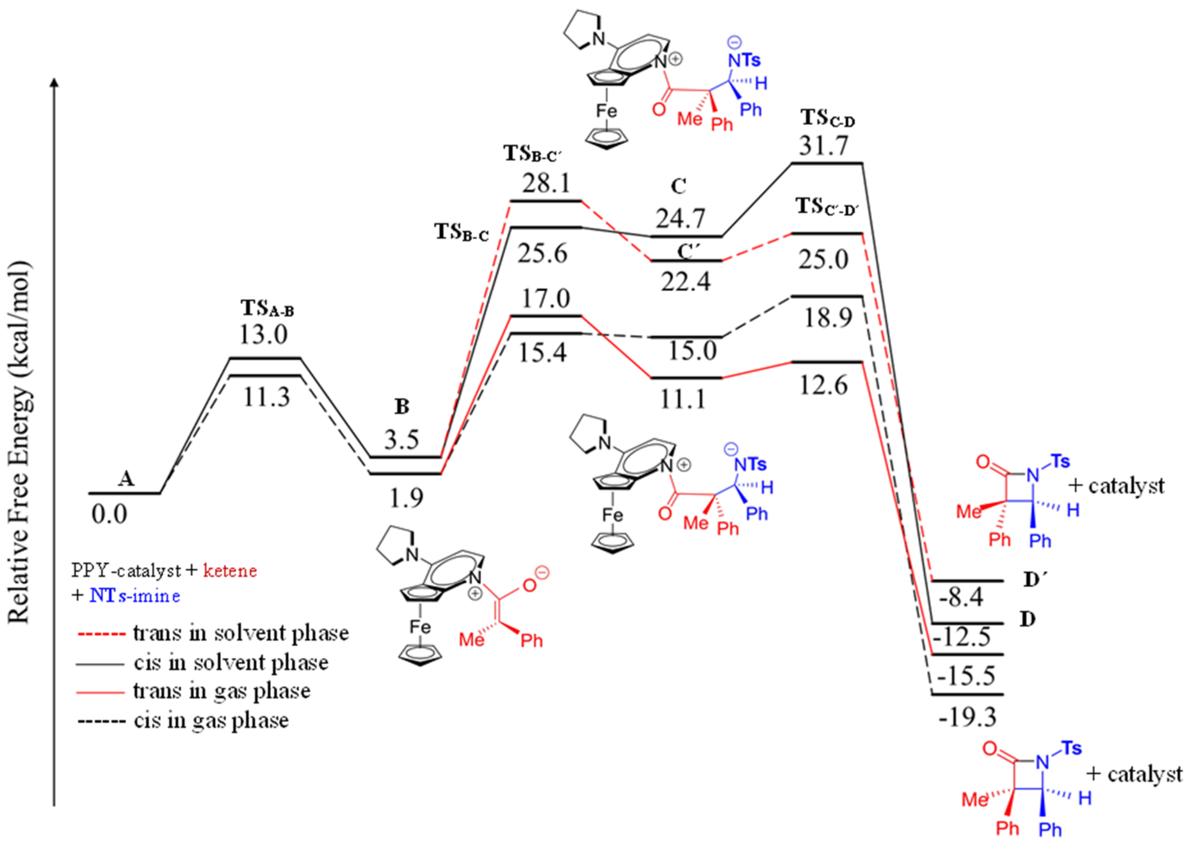

Fig. S3 Gibbs free energy profile for the ketene-first mechanism in gas and solvent (toluene) phase, comparing cis and trans selectivity of N-Ts imine.

Table S1. Optimized energies of reactants, intermediates, and products for the competing mechanisms of cycloaddition-catalyzed Staudinger reaction between ketene and N-Ts imine.

Name of molecules	Opt and Freq without dispersion for N-Ts, ketene-first (Hartree)	Opt and Freq with dispersion for N-Ts, ketene-first (Hartree)	Opt and Freq with dispersion for N-Ts, ketene-first in solvent phase (Hartree)
Reactants	-2655.011729	-2655.193217	-2635.312746
Intermediate-B	-2655.004973	-2655.190269	-2635.307168
Intermediate-C	-2654.964499	-2655.169312	-2635.273384
Intermediate-C`	-2654.970511	-2655.175527	-2635.277049
TSA-B	-2654.987763	-2655.175208	-2635.292029
TSB-C	-2654.963732	-2655.176642	-2635.271949
TSB-C`	-2654.961048	-2655.166103	-2635.267965
TSC-D	-2654.957243	-2655.163025	-2635.262228
TSC`-D`	-2654.968506	-2655.173100	-2635.272906
Catal+product-D	-2655.029491	-2655.223910	-2635.332666
Catal+product-D`	-2655.022172	-2655.217848	-2635.326291

Table S2. Optimized energies of reactants, intermediates, and products for the ketene-first mechanism of nucleophile-catalyzed Staudinger reaction involving N-Tf imine.

Name of molecules	Opt and Freq without dispersion for N-Tf, imine-first (Hartree)	Opt and Freq with dispersion for N-Tf, imine-first (Hartree)
Reactants	-2721.761756	-2721.930808
Intermediate-F	-2721.742033	-2721.925885
Intermediate-G	-2721.721492	-2721.915780
Intermediate-G`	-2721.713921	-2721.913348
TSE-F	-2721.740128	-2721.925978
TSF-G	-2721.717004	-2721.912138
TSF-G`	-2721.709054	-2721.904684
TSG-H	-2721.706676	-2721.885248
TSG`-H`	-2721.687089	-2721.879332
Catal+product-H	-2721.773150	-2721.955397
Catal+product-H`	-2721.771727	-2721.956774

continued

Name of molecules	Opt and Freq without dispersion for N-Tf, ketene-first (Hartree)	Opt and Freq with dispersion for N-Tf, ketene-first (Hartree)
Reactants	-2721.761756	-2721.930808
Intermediate-J	-2721.750431	-2721.927334
Intermediate-K	-2721.724645	-2721.916946
Intermediate-K`	-2721.732160	-2721.922293
TSE-J	-2721.746368	-2721.917550
TSJ-K	-2721.719600	-2721.910708
TSJ-K`	-2721.720711	-2721.911900
TSK-L	-2721.715227	-2721.909790
TSK`-L`	-2721.724689	-2721.915725
Catal+product-L	-2721.773150	-2721.956619
Catal+product-L`	-2721.771727	-2721.958543

Optimized molecular structure of reactants, intermediates, and products for the competing mechanisms of cycloaddition-catalyzed Staudinger reaction between ketene and NTs–imine.

A. PPY-catalyst + ketene + N-Ts imine

```
%nprocshared=16
%mem=800MB
# opt=modredundant freq=noraman b3lyp/gen pseudo=read

Title Card Required

0 1
 C                 -5.95098200   -1.96733800   -0.73282100
 C                 -5.87688400   -0.62866800   -0.21319400
 C                 -5.09105600   -0.66181500    0.98489500
 C                 -5.21815000   -2.83000200    0.15468600
 C                 -4.68432400   -2.02328500    1.21159800
 Fe                -3.97867500   -1.35655600   -0.63155800
 C                  0.85828500   -4.70460000    0.55994500
 C                  0.85995200   -4.30467300    2.04313400
 C                  0.48363800   -2.81970300    1.99795600
 N                 -0.39530500   -2.70968200    0.82134800
 C                 -0.32420700   -3.91439700   -0.02126500
 C                 -0.95985400   -1.52369600    0.46375000
 C                 -0.70281900   -0.34500200    1.18096100
 C                 -1.22487800    0.89751300    0.75965100
 N                 -2.00237900    1.10484200   -0.28947200
 C                 -2.32031300   -0.03058900   -0.98852600
 C                 -1.84060600   -1.36922900   -0.67485600
 H                  0.74907300   -5.78215300    0.40187400
```

H	1.79116600	-4.38095400	0.08702900
H	0.10392700	-4.87420500	2.59829900
H	1.82899300	-4.45949000	2.52480700
H	-0.04560000	-2.48769200	2.89919700
H	1.37664600	-2.19303700	1.87562900
H	-1.25968000	-4.48963000	0.04369800
H	-0.16310100	-3.64448100	-1.06844500
H	-0.03907300	-0.35539000	2.03579600
H	-0.95099600	1.78429700	1.33124500
C	-3.20798100	-1.49156600	-2.54780900
H	-3.77467800	-1.88774700	-3.38079800
C	-2.42905900	-2.26854200	-1.64000700
H	-2.29837200	-3.33782600	-1.69939000
C	-3.18640400	-0.13127000	-2.12566800
H	-3.70607600	0.69918800	-2.58437100
C	-6.71367100	-2.39945900	-1.95312700
H	-7.74813200	-2.67328600	-1.70122000
H	-6.76090600	-1.60166400	-2.70156000
H	-6.25328900	-3.27101200	-2.43059000
C	-5.08710800	-4.32140800	0.02454600
H	-5.90945400	-4.83867700	0.53912900
H	-5.10840400	-4.64066200	-1.02280400
H	-4.15105300	-4.68550800	0.46111900
C	-3.88905600	-2.51056800	2.38919900
H	-4.54184300	-2.71211700	3.25045200
H	-3.35297100	-3.43716400	2.16104000
H	-3.14611800	-1.77116000	2.70568300
C	-4.77842000	0.50409200	1.87765600
H	-5.50373200	0.57900000	2.70043100
H	-3.78197400	0.41332300	2.32124500
H	-4.80122800	1.44863600	1.32650500
C	-6.53504500	0.58609300	-0.80366600
H	-6.63563900	0.50334100	-1.89081200
H	-7.54363700	0.73228600	-0.39162200
H	-5.95950300	1.49330100	-0.59542100
C	-0.76679500	5.87124300	-1.26222700
O	-0.19038300	6.42696900	-2.12458700
C	-1.43058400	5.22778400	-0.31588900
C	-0.93627900	5.20499200	1.07563200
C	-1.64518200	4.49863000	2.06336700
C	0.23705700	5.88347000	1.46247700
C	-1.19548300	4.47292800	3.38553600
H	-2.54961300	3.96085100	1.79942000
C	0.68087400	5.85410700	2.78007100
H	0.80724900	6.44063700	0.72189700
C	-0.03290600	5.14826600	3.75413800
H	-1.76357400	3.91889900	4.12855700
H	1.58929600	6.38707800	3.04914400
H	0.31431700	5.12734200	4.78329200
C	-2.72538900	4.53887800	-0.73025900
H	-2.65449200	3.45410300	-0.57784600
H	-3.56865800	4.93123600	-0.14772400
H	-2.94604700	4.71583500	-1.78777100
S	4.29145600	-2.00912200	0.33011400

```
 O                  3.98187000   -2.04150500    1.76458400
 O                  3.99352900   -3.17083300   -0.51701900
 N                  3.37717200   -0.72718200   -0.30018800
 C                  3.93681300    0.27373300   -0.88166400
 H                  5.02609800    0.34632900   -0.98995000
 C                  3.17258500    1.38336600   -1.44340700
 C                  3.86425600    2.41213100   -2.10558700
 C                  1.77177900    1.44526100   -1.33327000
 C                  3.16686600    3.48622700   -2.65280200
 H                  4.94800600    2.36424200   -2.18805100
 C                  1.07924000    2.52159100   -1.87627800
 H                  1.24539500    0.65386300   -0.81025900
 C                  1.77512400    3.54199600   -2.53767300
 H                  3.70429300    4.28030100   -3.16345700
 H                 -0.00031100    2.56500400   -1.76935400
 H                  1.23435300    4.38859400   -2.95030300
 C                  8.05291300   -0.64607300    1.02444500
 C                  6.69688600   -0.93104100    1.16770900
 C                  6.03975400   -1.63638100    0.15556000
 C                  6.72352600   -2.05666300   -0.98769000
 C                  8.08048000   -1.76197700   -1.11290300
 C                  8.76540900   -1.05610400   -0.11321700
 H                  8.56897200   -0.10101600    1.81105100
 H                  6.15224300   -0.62848600    2.05609500
 H                  6.19956000   -2.61824200   -1.75414100
 H                  8.61759000   -2.09041800   -1.99918200
 C                 10.24247800   -0.76897400   -0.24087900
 H                 10.50263400    0.19748300    0.20372800
 H                 10.83587700   -1.53446100    0.27637300
 H                 10.56128500   -0.76083600   -1.28797900

B 15 54 F
B 56 76 F

Fe 0
LANL2DZ
****
H C O N S 0
6-31G*
****

Fe 0
LANL2DZ
```

TS$_{A\text{-}B}$

```
%nprocshared=16
%mem=800MB
# opt=(modredundant,ts,calcfc,noeigen) freq=noraman b3lyp/gen pseudo=read

Title Card Required

0 1
 C                 -3.62344300   -1.05464500   -2.95354900
```

C	-3.36201300	0.35929100	-2.94310800
C	-4.28842200	0.97178900	-2.03854200
C	-4.72154900	-1.31149600	-2.06118500
C	-5.12612000	-0.06158100	-1.49070100
Fe	-3.13148000	-0.44926800	-1.03892100
C	-5.54166500	-2.05141800	4.14024300
C	-6.41876700	-0.79192400	4.18758900
C	-5.46292300	0.30935000	3.71465700
N	-4.56909300	-0.38001800	2.77171300
C	-4.69376000	-1.84335100	2.87679800
C	-3.63974400	0.28845200	2.03778100
C	-3.43278800	1.66885400	2.21649700
C	-2.41010400	2.34655700	1.53664400
N	-1.60992500	1.80304800	0.63288000
C	-1.79795000	0.46711600	0.37842400
C	-2.78379800	-0.35238200	1.06462800
H	-6.11705700	-2.98072600	4.10246900
H	-4.89261400	-2.09308100	5.02268400
H	-7.25847600	-0.88625700	3.48886700
H	-6.82824600	-0.58598700	5.18036900
H	-5.98192800	1.13607800	3.21703000
H	-4.88831200	0.73040100	4.55327500
H	-5.19701200	-2.25410000	1.99038700
H	-3.70872200	-2.31014900	2.95735800
H	-4.00628000	2.22322500	2.94764200
H	-2.21789200	3.39194700	1.76582900
C	-1.58233600	-1.68500000	-0.40656400
H	-1.23785200	-2.54663500	-0.96266200
C	-2.65131300	-1.69054900	0.53507300
H	-3.23133400	-2.56017400	0.80031000
C	-1.08804800	-0.35764200	-0.54449400
H	-0.28757400	-0.02483600	-1.18841000
C	-2.92553100	-2.07275700	-3.80977600
H	-3.41542400	-2.16650100	-4.78909800
H	-1.88163900	-1.79909400	-3.99412100
H	-2.93239900	-3.06520400	-3.34732600
C	-5.35874100	-2.64897800	-1.81157300
H	-6.16099600	-2.84654900	-2.53615200
H	-4.63426200	-3.46534300	-1.89807200
H	-5.80299100	-2.70611600	-0.81209500
C	-6.26929700	0.16125100	-0.54221600
H	-7.18773900	0.42313000	-1.08612100
H	-6.48636900	-0.73303900	0.05096100
H	-6.05748000	0.97920400	0.15422800
C	-4.42560500	2.44006100	-1.75499300
H	-5.27041100	2.86771100	-2.31285800
H	-4.60227600	2.63660900	-0.69206800
H	-3.52881100	2.99192200	-2.04692400
C	-2.33006700	1.06933900	-3.77303600
H	-1.46421600	0.42983600	-3.97159800
H	-2.74512500	1.36985100	-4.74518500
H	-1.96281200	1.97343800	-3.27840500
C	0.23908600	2.48629300	0.48150900
O	0.92861500	1.58282600	0.87058500

C	0.19324000	3.77309700	0.04006800
C	1.42266200	4.57855100	0.20288500
C	1.57743200	5.79495400	-0.49923500
C	2.47014100	4.21505600	1.08099000
C	2.71805100	6.58439300	-0.35161700
H	0.79897300	6.12944200	-1.17648700
C	3.60683700	5.00655700	1.22598700
H	2.37990100	3.30632600	1.66666200
C	3.74728800	6.19833100	0.50760100
H	2.79824100	7.51076100	-0.91622700
H	4.38636700	4.69214000	1.91620900
H	4.63388900	6.81546400	0.62517600
C	-0.97970000	4.38946900	-0.68039500
H	-1.30060400	5.33380800	-0.21709800
H	-0.72929000	4.62551000	-1.72563100
H	-1.83000600	3.71014100	-0.70253800
S	6.21772500	-2.00808300	1.43627900
O	5.81896500	-1.71529200	2.81518900
O	7.55781200	-2.51006200	1.12598100
N	5.14651700	-3.23784500	0.91236000
C	4.19882700	-2.97748700	0.08808300
H	4.05123700	-1.98381200	-0.34966700
C	3.24765300	-4.00885400	-0.34528600
C	2.28542600	-3.68151600	-1.31313000
C	3.28567800	-5.31305300	0.17794900
C	1.37713700	-4.64182900	-1.75820200
H	2.25732700	-2.67296200	-1.71919100
C	2.37431100	-6.26671400	-0.26268600
H	4.03502100	-5.55385500	0.92506200
C	1.41974600	-5.93458900	-1.23165200
H	0.64464500	-4.38535800	-2.51901000
H	2.40610200	-7.27330000	0.14494900
H	0.71349500	-6.68513300	-1.57671300
C	4.59880500	1.38991200	-0.08936500
C	4.83609800	0.28133900	0.71887100
C	5.92635800	-0.54978500	0.43675100
C	6.78255400	-0.27209700	-0.63050900
C	6.54347800	0.85671200	-1.41423700
C	5.45032600	1.69769400	-1.16337500
H	3.74359700	2.02520800	0.11821100
H	4.18813800	0.06747900	1.56319700
H	7.62881000	-0.92292700	-0.82280800
H	7.21878700	1.08826800	-2.23441500
C	5.17769900	2.90582600	-2.02741500
H	4.94858800	3.78641000	-1.41758800
H	6.03229500	3.14043700	-2.67010300
H	4.30909300	2.73505100	-2.67667400

H C O N S 0
6-31G*
****
Fe 0
LANL2DZ
****

```
Fe 0
LANL2DZ
```

Intermediate B

```
nprocshared=16
%mem=800MB
# opt=(modredundant) freq=noraman b3lyp/gen pseudo=read

Title Card Required

0 1
 C                  4.87986700    1.66431900    1.17225800
 C                  4.17718200    2.48424300    0.22319400
 C                  4.12952400    1.77431500   -1.01951900
 C                  5.27728700    0.45275100    0.50796600
 C                  4.80477200    0.51674700   -0.84300500
 Fe                 3.21161600    0.64828300    0.49146500
 C                  2.13527100   -4.93535700   -1.39304400
 C                  2.17782600   -4.55910200   -2.88033400
 C                  1.48007900   -3.19621600   -2.90339700
 N                  1.85009900   -2.58587100   -1.61158200
 C                  2.38155700   -3.60019400   -0.67698300
 C                  1.41916200   -1.36032300   -1.25927300
 C                  0.62697000   -0.58549300   -2.14499300
 C                  0.01000800    0.58660400   -1.73253800
 N                  0.18835800    1.14806200   -0.53085700
 C                  1.06378500    0.51886900    0.33472900
 C                  1.67751900   -0.75939100    0.03179900
 H                  2.87421800   -5.69287900   -1.11617700
 H                  1.14013100   -5.30303500   -1.12601800
 H                  3.21391100   -4.46081600   -3.22712900
 H                  1.67360000   -5.28566100   -3.52334900
 H                  1.80818800   -2.56356100   -3.73489600
 H                  0.38888200   -3.30669100   -2.95191100
 H                  3.45192900   -3.42498400   -0.50349200
 H                  1.84645700   -3.54807900    0.27139300
 H                  0.37562100   -0.96073700   -3.12687600
 H                 -0.70965900    1.08796800   -2.37117100
 C                  2.27034900   -0.11104700    2.17913100
 H                  2.72810000   -0.10262100    3.15943500
 C                  2.44856500   -1.12669000    1.19723200
 H                  3.04852300   -2.01283400    1.32684100
 C                  1.45380600    0.92509100    1.63990400
 H                  1.14055500    1.83563600    2.12963600
 C                  5.20587300    2.04370800    2.58872200
 H                  6.15015300    2.60368700    2.63875100
 H                  4.42859100    2.67792000    3.02688100
 H                  5.31649200    1.16349800    3.23020400
 C                  6.08087000   -0.66156100    1.11595500
 H                  7.15818900   -0.46717500    1.02112500
 H                  5.86548600   -0.78325800    2.18268000
 H                  5.87905800   -1.61999600    0.62662500
```

C	5.03102700	-0.50396700	-1.92147000
H	5.93034000	-0.26559600	-2.50665200
H	5.16793000	-1.50926800	-1.51013700
H	4.18870300	-0.54215700	-2.61949100
C	3.55537600	2.26624600	-2.31774700
H	4.35690300	2.55828200	-3.01043600
H	2.95588600	1.49675100	-2.81602900
H	2.91661200	3.14079500	-2.17298600
C	3.64529500	3.86470000	0.48365300
H	3.34016600	3.98779800	1.52730300
H	4.41161000	4.62436200	0.27507700
H	2.77645500	4.09193700	-0.13954100
C	-0.96627800	2.02651000	0.08256700
O	-1.65438200	1.33661600	0.86643900
C	-1.03265600	3.32728700	-0.37658400
C	-2.15440300	4.19691000	0.01601300
C	-2.30811900	5.47497500	-0.57544500
C	-3.13006800	3.83027900	0.97869400
C	-3.36219600	6.32284800	-0.23765900
H	-1.59525700	5.81578100	-1.31878600
C	-4.18033000	4.68289600	1.31054700
H	-3.03832500	2.86903900	1.46708600
C	-4.31266600	5.93696000	0.70804400
H	-3.43777500	7.29442800	-0.72193500
H	-4.90442600	4.36160500	2.05688500
H	-5.13444800	6.59777900	0.97196700
C	0.00458700	3.90677000	-1.31065900
H	-0.39904600	4.14430700	-2.30807100
H	0.41698300	4.84795500	-0.91769800
H	0.84157700	3.22283100	-1.45458900
S	-2.14136500	-3.68424100	0.15955800
O	-2.74995000	-5.01583300	0.17509600
O	-0.72457300	-3.53525100	-0.22708400
N	-3.03093600	-2.78609400	-0.98328300
C	-3.31067800	-1.55048700	-0.75505200
H	-3.03312400	-1.03766200	0.17123000
C	-4.00228000	-0.71007800	-1.73253500
C	-4.09777000	0.66739700	-1.46567400
C	-4.55423700	-1.23584100	-2.91513600
C	-4.73409400	1.51147500	-2.37588200
H	-3.65239100	1.06969200	-0.55842900
C	-5.19489600	-0.38997000	-3.81311600
H	-4.47600200	-2.30189500	-3.10468400
C	-5.28458500	0.98317500	-3.54537000
H	-4.79554600	2.57538800	-2.16655300
H	-5.62876700	-0.79459500	-4.72358300
H	-5.78627700	1.63882800	-4.25250700
C	-3.44974600	-3.00852600	3.93034500
C	-3.25099600	-3.57302900	2.67193900
C	-2.36318800	-2.95886500	1.78463300
C	-1.68576100	-1.78849500	2.14149200
C	-1.91667700	-1.22697000	3.39524400
C	-2.79241200	-1.82900000	4.31022200
H	-4.13374800	-3.48770400	4.62658300

```
 H                  -3.76358500   -4.48160000    2.37543500
 H                  -1.01044400   -1.29720900    1.45133400
 H                  -1.41944600   -0.29481600    3.64803300
 C                  -3.01080300   -1.22504500    5.67707200
 H                  -3.95779600   -1.55545400    6.11568300
 H                  -2.20901900   -1.51622500    6.36880700
 H                  -3.01803600   -0.13079300    5.63191500

 B 56 76 F

H C O N S 0
6-31G*
****
Fe 0
LANL2DZ
****

Fe 0
LANL2DZ
```

TS$_{B\text{-}C}$'

```
%nprocshared=16
%mem=1GB
# freq=noraman b3lyp/gen pseudo=read

Title Card Required

0 1
 C                   4.54981300   -2.63308600   -1.08499500
 C                   3.52088800   -2.86137900   -0.10791900
 C                   3.76143300   -1.98272700    0.99768700
 C                   5.43412900   -1.62175000   -0.57414300
 C                   4.94304400   -1.21555500    0.70860400
 Fe                  3.50615700   -0.87549600   -0.75980200
 C                   4.99639800    4.75226900    0.41516700
 C                   4.76112400    4.57022700    1.92138000
 C                   3.47786200    3.73428600    1.96985100
 N                   3.55044700    2.90314300    0.75283000
 C                   4.58737000    3.39428000   -0.17353600
 C                   2.63625000    1.94403700    0.48454800
 C                   1.52576700    1.75566600    1.33495900
 C                   0.48890900    0.89762000    1.00649600
 N                   0.50548200    0.07781600   -0.05521900
 C                   1.60774300    0.14614700   -0.90194600
 C                   2.68474900    1.09172600   -0.68121300
 H                   6.02817300    5.01474400    0.16513100
 H                   4.34336500    5.54024500    0.02292800
 H                   5.59107600    4.01388000    2.37293100
 H                   4.65605900    5.51576600    2.46002000
 H                   3.41584100    3.10112500    2.86096600
 H                   2.58130100    4.36897600    1.94150400
 H                   5.43744800    2.69945400   -0.19873700
```

H	4.18429600	3.48415700	-1.18495000
H	1.37517800	2.39254200	2.19515400
H	-0.42855800	0.91780500	1.58243900
C	3.09260800	-0.08688800	-2.63473300
H	3.56308200	-0.44190200	-3.54192000
C	3.61816200	0.91280200	-1.77104700
H	4.54310200	1.44224900	-1.93094800
C	1.88290200	-0.59639800	-2.08286500
H	1.24920400	-1.35875200	-2.50636500
C	4.71055500	-3.37358400	-2.38231300
H	5.29862300	-4.29100700	-2.24085700
H	3.74415000	-3.66789800	-2.80355100
H	5.22846600	-2.76878800	-3.13364400
C	6.68169700	-1.12085200	-1.24419300
H	7.55410300	-1.72049800	-0.94958200
H	6.60667000	-1.17285000	-2.33510600
H	6.89693200	-0.08118700	-0.97628100
C	5.59216900	-0.22945300	1.63790500
H	6.26105200	-0.73842600	2.34613000
H	6.19682600	0.50422000	1.09443600
H	4.85135200	0.31875300	2.22891500
C	2.98272400	-1.91857500	2.28020000
H	3.47978200	-2.50574300	3.06503300
H	2.88923900	-0.89196800	2.64968300
H	1.97308600	-2.31798000	2.16259700
C	2.42549300	-3.88381000	-0.21335500
H	2.13274500	-4.05746800	-1.25316400
H	2.74993100	-4.84831800	0.20137200
H	1.52785100	-3.57864500	0.33128200
C	-0.75987800	-0.60754400	-0.56992000
O	-0.94847800	-0.43996500	-1.76603400
C	-1.55556700	-1.35616100	0.37426100
C	-2.44632300	-2.37388200	-0.27549200
C	-2.60012400	-3.66184000	0.28174500
C	-3.20214600	-2.09874500	-1.43918400
C	-3.44572100	-4.61597400	-0.28596000
H	-2.05528800	-3.93760800	1.17646700
C	-4.05219800	-3.05075900	-1.99970700
H	-3.11754700	-1.13200400	-1.91515100
C	-4.18254100	-4.31987100	-1.43163400
H	-3.52659400	-5.59623900	0.17826000
H	-4.61798500	-2.79304100	-2.89210700
H	-4.84487000	-5.06027900	-1.87267600
C	-0.88901300	-1.84072100	1.65760200
H	-1.63773200	-2.19309900	2.37027400
H	-0.20379500	-2.68082100	1.46985100
H	-0.31833700	-1.06952100	2.17240800
S	-2.45588700	2.59113800	0.26886300
O	-2.68535200	3.83079800	1.02597400
O	-1.20887200	2.48665000	-0.52217200
N	-2.57358600	1.36047700	1.37267200
C	-3.05470700	0.15612500	1.03822800
H	-3.55314300	0.01072400	0.07838300
C	-3.65555500	-0.61743900	2.16202800

```
 C                  -4.63014600   -1.59570300    1.90919300
 C                  -3.32420400   -0.32667500    3.49723400
 C                  -5.24321100   -2.27785800    2.96196600
 H                  -4.91717600   -1.81558400    0.88652300
 C                  -3.92990300   -1.01478100    4.54532600
 H                  -2.60778100    0.46370000    3.69443900
 C                  -4.89110100   -1.99640900    4.28230700
 H                  -5.99968800   -3.02765700    2.74563000
 H                  -3.66257900   -0.77659400    5.57195100
 H                  -5.36896100   -2.52756600    5.10164500
 C                  -6.13943100    2.75284100   -1.44561900
 C                  -5.08817200    2.87830500   -0.54149300
 C                  -3.81688400    2.42140000   -0.90117600
 C                  -3.59270100    1.84971100   -2.15455800
 C                  -4.65924800    1.73248200   -3.04909000
 C                  -5.94291100    2.17918400   -2.71214000
 H                  -7.12899400    3.10945500   -1.16791700
 H                  -5.23927100    3.33821500    0.42981900
 H                  -2.59783200    1.50513700   -2.41710500
 H                  -4.48746300    1.28772800   -4.02669500
 C                  -7.08834400    2.07129300   -3.69180100
 H                  -8.00118400    1.71171100   -3.20287700
 H                  -7.32418600    3.04767900   -4.13557400
 H                  -6.85120500    1.38607700   -4.51194900

H C O N S 0
6-31G*
****
Fe 0
LANL2DZ
****

Fe 0
LANL2DZ
```

Intermediate C

```
%nprocshared=16
%mem=800MB
# freq=noraman b3lyp/gen pseudo=read

Title Card Required

0 1
 C                   5.27179900    1.78496000   -1.27536000
 C                   4.01296700    2.17868100   -1.84427100
 C                   3.38359200    1.00692700   -2.38084000
 C                   5.42245600    0.36749800   -1.46508900
 C                   4.25580400   -0.11129200   -2.14799500
 Fe                  3.72360100    0.77875000   -0.33712700
 C                   4.38220500   -4.63254700    1.96128100
 C                   3.74188800   -5.29358300    0.73249000
```

C	2.55819100	-4.37332100	0.41942400
N	3.02541600	-3.03498900	0.84826900
C	4.25076700	-3.13191400	1.66502100
C	2.32044500	-1.91802300	0.59347000
C	1.05311000	-2.00094300	-0.03021700
C	0.24430100	-0.90401100	-0.22002300
N	0.59326300	0.36227000	0.13865900
C	1.88678700	0.55661900	0.66857600
C	2.75159900	-0.58299100	0.94777600
H	5.42263600	-4.92819000	2.12153300
H	3.81686900	-4.88519500	2.86555900
H	4.44653100	-5.30092300	-0.10757000
H	3.42269700	-6.32323100	0.91301200
H	2.29775900	-4.36092800	-0.64265100
H	1.66108900	-4.65487000	0.98571900
H	5.11389000	-2.75942800	1.09776400
H	4.15012000	-2.53447000	2.57397400
H	0.61295900	-2.95404000	-0.29194800
H	-0.80742000	-1.05126600	-0.52662300
C	3.79710900	1.35145800	1.67210400
H	4.53590000	2.03156200	2.07466500
C	3.95472700	-0.05208100	1.54484800
H	4.82651600	-0.60609600	1.85162100
C	2.54473800	1.73961500	1.11730100
H	2.17364200	2.74462000	1.02300400
C	6.28674500	2.70060400	-0.65207200
H	6.99554500	3.06976700	-1.40607600
H	5.81654600	3.57559900	-0.19299100
H	6.87112100	2.19342900	0.12254200
C	6.62246800	-0.44912700	-1.07640400
H	7.37083200	-0.45886600	-1.88086400
H	7.11205700	-0.04993700	-0.18226800
H	6.35504700	-1.49116800	-0.87135600
C	4.00285700	-1.52210200	-2.59652800
H	4.35118000	-1.67391300	-3.62728700
H	4.52510700	-2.24883000	-1.96592200
H	2.93619700	-1.76590800	-2.57204900
C	2.07981200	0.97133500	-3.12553600
H	2.24433400	1.10739400	-4.20343500
H	1.56055800	0.01615600	-2.99655000
H	1.40538200	1.76595000	-2.79456200
C	3.45831000	3.57381700	-1.90517600
H	3.89666300	4.21571200	-1.13494600
H	3.67423900	4.03680500	-2.87781700
H	2.37393700	3.58259100	-1.76206500
C	-0.27188700	1.51583500	-0.09628500
O	0.29489300	2.58266600	-0.20234700
C	-1.81762400	1.41088700	-0.29308700
S	-3.37072300	-2.22419200	0.30173800
O	-2.64524500	-3.41596300	-0.19653800
O	-3.93445900	-2.26349600	1.66993800
N	-2.42667300	-0.96976200	-0.00256900
C	-5.77401900	-2.11343300	-3.00038900
C	-4.69256800	-2.32862300	-2.15063500

```
 C                  -4.80514500   -2.02530300   -0.78924400
 C                  -6.00127300   -1.51046100   -0.28906100
 C                  -7.07897200   -1.29456100   -1.15270600
 C                  -6.98531200   -1.59112400   -2.51691800
 H                  -5.68340700   -2.35933800   -4.05707900
 H                  -3.76394000   -2.74764000   -2.52551700
 H                  -6.08409400   -1.30452200    0.77315400
 H                  -8.01058500   -0.89579000   -0.75596900
 C                  -8.16034500   -1.38479000   -3.44539300
 H                  -7.85429200   -0.90343400   -4.38215500
 H                  -8.63012700   -2.34036200   -3.71501800
 H                  -8.93150400   -0.76015300   -2.98247400
 C                  -2.67053400    0.33389300    0.55528700
 C                  -2.40566000    2.80853000    0.01791600
 C                  -3.43925700    3.33620900   -0.76896300
 C                  -2.00199900    3.55272900    1.13863900
 C                  -4.03817100    4.56041200   -0.46047000
 H                  -3.79785000    2.79314900   -1.63572700
 C                  -2.59246700    4.77695000    1.44730100
 H                  -1.21778400    3.17678400    1.78497800
 C                  -3.61492900    5.29091800    0.64787500
 H                  -4.83686100    4.93790300   -1.09386000
 H                  -2.25298400    5.32668200    2.32139000
 H                  -4.07588600    6.24528600    0.88862700
 H                  -3.70061000    0.67370200    0.34773600
 C                  -2.47700000    0.46160300    2.07491000
 C                  -3.33956100    1.26893900    2.82826600
 C                  -1.46763000   -0.23398300    2.75318200
 C                  -3.18036600    1.40811000    4.20820900
 H                  -4.15096900    1.79089000    2.32818100
 C                  -1.29811900   -0.09288500    4.13085600
 H                  -0.84618200   -0.93398300    2.20533400
 C                  -2.14974800    0.73531300    4.86530400
 H                  -3.86840000    2.03587200    4.76926900
 H                  -0.51309200   -0.65078100    4.63648200
 H                  -2.02488900    0.83680400    5.94054800
 C                  -1.98183900    1.08701500   -1.80946400
 H                  -3.02585400    0.85553500   -2.03247900
 H                  -1.66124100    1.94318900   -2.41241800
 H                  -1.40849500    0.20861000   -2.10431200

Fe 0
LANL2DZ
****
H C O N S 0
6-31G*
****

Fe 0
LANL2DZ
```

Intermediate C′

```
%nprocshared=16
%mem=1GB
# freq=noraman b3lyp/gen pseudo=read

Title Card Required

0 1
 C                  5.18210800   -1.98648000    0.47512300
 C                  3.96400100   -2.15840800    1.21866400
 C                  3.73371700   -0.96102800    1.97083600
 C                  5.70861000   -0.68347200    0.77781400
 C                  4.81008200   -0.04793500    1.69508400
 Fe                 3.82482500   -0.52592300   -0.07831900
 C                  4.36765000    5.33093400   -0.94156200
 C                  3.80808700    5.65708900    0.45030700
 C                  2.65644300    4.65739000    0.60240700
 N                  3.10513100    3.47886300   -0.16747700
 C                  4.27282100    3.80039000   -1.01013200
 C                  2.40100800    2.32922700   -0.19605300
 C                  1.14949600    2.23597600    0.45786800
 C                  0.30695000    1.15329100    0.29087500
 N                  0.65937300    0.04400000   -0.39580600
 C                  1.93364200    0.00623300   -0.96395700
 C                  2.82184800    1.15028200   -0.92040500
 H                  5.39078500    5.68587200   -1.09314600
 H                  3.73652500    5.77815600   -1.71789400
 H                  4.56852400    5.47760800    1.21961000
 H                  3.46792200    6.69136900    0.54776700
 H                  2.46698600    4.38153000    1.64449200
 H                  1.72200000    5.04857000    0.17823900
 H                  5.17683300    3.32730900   -0.60377500
 H                  4.11958900    3.43681500   -2.02861500
 H                  0.73111500    3.09058700    0.97041100
 H                 -0.74166600    1.17596100    0.59161900
 C                  3.81481600   -0.54144700   -2.15493500
 H                  4.52915800   -1.10035300   -2.74450500
 C                  4.00587500    0.77775100   -1.66129900
 H                  4.88521200    1.37779800   -1.83021700
 C                  2.56319900   -1.03920100   -1.69182000
 H                  2.13971400   -2.00950400   -1.90183100
 C                  5.82353700   -3.01739200   -0.40944700
 H                  6.44331400   -3.70813800    0.17864800
 H                  5.07688400   -3.61883600   -0.93788900
 H                  6.47290400   -2.55901500   -1.16193700
 C                  6.99622400   -0.11044700    0.25767900
 H                  7.84337700   -0.40422500    0.89252600
 H                  7.21438900   -0.45738400   -0.75748700
 H                  6.97192000    0.98405100    0.23512200
 C                  4.99888900    1.29570100    2.33983200
 H                  5.56464000    1.20260900    3.27737400
 H                  5.55419000    1.98486000    1.69484200
 H                  4.04100000    1.76499000    2.58603000
 C                  2.62500000   -0.71612400    2.95422700
 H                  2.99053600   -0.82871200    3.98427400
```

H	2.20815500	0.29236300	2.86347900
H	1.80326900	-1.42348000	2.82265400
C	3.11930300	-3.40049600	1.23485200
H	3.18125500	-3.94267700	0.28649500
H	3.44743200	-4.08718700	2.02742200
H	2.06393800	-3.17558000	1.41190100
C	-0.38872900	-0.88913800	-0.96678500
O	-0.31988200	-1.04373700	-2.15538700
C	-1.36113800	-1.66121900	-0.04523300
C	-1.38290300	-3.12860400	-0.52541000
C	-1.00379700	-4.18529800	0.31630200
C	-1.79444900	-3.45627400	-1.83158200
C	-1.03408200	-5.51322600	-0.12046300
H	-0.68770600	-3.98973400	1.33434800
C	-1.82482400	-4.77953900	-2.26886800
H	-2.08259700	-2.67008100	-2.51788600
C	-1.44471300	-5.81828100	-1.41579200
H	-0.73882600	-6.30632500	0.56203300
H	-2.14975400	-4.99738700	-3.28302800
H	-1.47208500	-6.84981500	-1.75718700
C	-0.99164300	-1.54487700	1.43902000
H	-1.66228000	-2.16735300	2.03527500
H	0.03795300	-1.86182400	1.63253100
H	-1.11482300	-0.51958100	1.78604600
S	-3.00349600	1.60209700	-1.08360400
O	-2.11358500	2.77965900	-0.92310900
O	-3.22519700	1.06607100	-2.44961600
N	-2.48756500	0.49795600	-0.04060000
C	-2.77842200	-0.89627500	-0.29980700
H	-3.02121600	-1.07275600	-1.35485100
C	-3.89897200	-1.47616700	0.56467400
C	-4.59331000	-2.63251100	0.17789000
C	-4.26968600	-0.84806600	1.76166300
C	-5.61506600	-3.15540300	0.97255500
H	-4.33850200	-3.12444100	-0.75622100
C	-5.28905800	-1.36961800	2.55941900
H	-3.76499500	0.07296500	2.03376100
C	-5.96360200	-2.52886000	2.17079200
H	-6.14426100	-4.04891400	0.64982200
H	-5.56489900	-0.86246000	3.48114600
H	-6.76231200	-2.93282600	2.78821200
C	-5.98189100	3.54815800	0.92445800
C	-4.72823800	3.13685100	0.47583600
C	-4.63221800	2.17975000	-0.53738200
C	-5.79133200	1.64656200	-1.10241900
C	-7.04127400	2.06547800	-0.64483000
C	-7.15881600	3.01583200	0.37753400
H	-6.05051600	4.30238800	1.70639100
H	-3.82088300	3.56932000	0.88508600
H	-5.70567700	0.92690700	-1.90973700
H	-7.94125800	1.65142000	-1.09519700
C	-8.51504900	3.44416200	0.88956700
H	-8.50445100	4.48413100	1.23500100
H	-9.28364200	3.35373900	0.11413900

```
 H                  -8.83652000    2.82443800    1.73814700

Fe 0
LANL2DZ
****
H C O N S 0
6-31G*
****

Fe 0
LANL2DZ
```

TS$_{C-D}$

```
nprocshared=16
%mem=800MB
# opt=(ts,calcfc,noeigen) freq=noraman b3lyp/gen pseudo=read

Title Card Required

0 1
 C                  -4.37824200    2.07552400    1.75916000
 C                  -3.12250100    1.75318200    2.37859900
 C                  -3.04478400    0.32805000    2.50362000
 C                  -5.08873200    0.84849800    1.52347800
 C                  -4.25951800   -0.22936000    1.97194600
 Fe                 -3.29069000    0.86374100    0.48463500
 C                  -5.70468000   -3.33657900   -2.92292000
 C                  -5.31761700   -4.44748000   -1.93635900
 C                  -3.90628000   -4.04151000   -1.49693900
 N                  -3.92476400   -2.56896900   -1.55865500
 C                  -5.10721000   -2.07367300   -2.28595800
 C                  -2.86500600   -1.82097500   -1.16701900
 C                  -1.66563100   -2.43141400   -0.74655000
 C                  -0.50047400   -1.69791800   -0.53351300
 N                  -0.44821800   -0.36455000   -0.57089800
 C                  -1.63309200    0.31172100   -0.81887600
 C                  -2.85567500   -0.37585600   -1.18956300
 H                  -6.78372300   -3.24881200   -3.07782800
 H                  -5.23788400   -3.51799000   -3.89793500
 H                  -5.99646600   -4.44792300   -1.07513900
 H                  -5.33817500   -5.44637200   -2.38042100
 H                  -3.66316200   -4.37899900   -0.48405100
 H                  -3.14179900   -4.44113000   -2.17838300
 H                  -5.81774200   -1.60495900   -1.59138800
 H                  -4.81578600   -1.33036900   -3.03190800
 H                  -1.57145500   -3.50844400   -0.72169000
 H                   0.44614800   -2.21298700   -0.41026300
 C                  -3.20214800    1.91321900   -1.30917900
 H                  -3.67793700    2.87441800   -1.45195400
 C                  -3.83158900    0.64894300   -1.48320400
 H                  -4.85614800    0.50634200   -1.78790700
 C                  -1.86854500    1.71482000   -0.85222300
 H                  -1.14024100    2.48270700   -0.63556200
```

C	-4.88744400	3.46317800	1.49115300
H	-5.41522100	3.86523400	2.36737400
H	-4.07027300	4.15338100	1.25819500
H	-5.58959800	3.48427700	0.65138800
C	-6.47638300	0.72810900	0.96070600
H	-7.23081900	0.79243800	1.75702500
H	-6.69489800	1.52459400	0.24179700
H	-6.62590600	-0.22958600	0.45086500
C	-4.61257200	-1.68905500	1.98395400
H	-5.06503700	-1.97418800	2.94413600
H	-5.33047600	-1.94415100	1.19806200
H	-3.72607500	-2.31597900	1.84259400
C	-1.95637600	-0.47844300	3.15394100
H	-2.30442200	-0.89895700	4.10731600
H	-1.63806600	-1.31565000	2.52290500
H	-1.07170400	0.12538400	3.36597400
C	-2.12945200	2.76572700	2.87509200
H	-1.95314900	3.56041200	2.14298400
H	-2.49200400	3.24114900	3.79700600
H	-1.16271700	2.31067900	3.10181100
C	0.92035700	0.41585100	-1.02077000
O	0.84852400	0.72530500	-2.18989000
C	1.63254700	1.23873000	0.12005700
C	1.62562300	2.75621200	-0.09686000
C	1.48683500	3.64092700	0.98838200
C	1.78240500	3.32792800	-1.37442400
C	1.50432400	5.02664100	0.81282400
H	1.36821600	3.25920500	1.99564000
C	1.79367400	4.71153800	-1.55134800
H	1.87981400	2.68188900	-2.23466400
C	1.65650500	5.57147800	-0.46099200
H	1.40070800	5.67613800	1.67874900
H	1.91621800	5.11619000	-2.55279800
H	1.67096400	6.64929100	-0.60187700
C	1.07491400	0.84230600	1.49481600
H	1.69081500	1.25807700	2.29820500
H	0.04887500	1.19375000	1.62702200
H	1.09079400	-0.24387800	1.59537600
S	3.12058400	-2.12631300	-0.94406300
O	2.07867600	-3.17452000	-1.06713700
O	4.08244700	-1.99168000	-2.05213200
N	2.33198200	-0.75734900	-0.52452800
C	3.00427200	0.46098500	-0.05150600
H	3.45007500	0.31263500	0.94325900
C	4.06961400	1.11250000	-0.92037400
C	5.07575500	1.85716900	-0.28930700
C	4.08407800	1.01682500	-2.31855700
C	6.06495800	2.50662500	-1.02916700
H	5.08414100	1.92897000	0.79683500
C	5.07420900	1.66193900	-3.05988500
H	3.32716100	0.42602800	-2.81889300
C	6.06580900	2.41061300	-2.42148100
H	6.83517300	3.07946600	-0.51837800
H	5.07494500	1.57047500	-4.14312400

```
 H                  6.83799900    2.90708700   -3.00402800
 C                  4.10682700   -2.52462900    0.51761400
 C                  3.52568500   -3.22566800    1.57692800
 C                  5.44235000   -2.12426100    0.58460100
 C                  4.28679400   -3.51926900    2.70645900
 H                  2.49578900   -3.55843500    1.49765300
 C                  6.19081600   -2.42136300    1.72436200
 H                  5.88798800   -1.60434400   -0.25691800
 C                  5.62781600   -3.11762500    2.80213100
 H                  3.83715900   -4.07793100    3.52473600
 H                  7.23351200   -2.11503000    1.77172300
 C                  6.43799100   -3.41559100    4.04262400
 H                  7.50486900   -3.50610200    3.81247000
 H                  6.33313100   -2.61551600    4.78818900
 H                  6.11356800   -4.34696900    4.51931700

H C O N S 0
6-31G*
****
Fe 0
LANL2DZ
****

Fe 0
LANL2DZ
```

TS$_{C'-D'}$

```
%nprocshared=16
%mem=1GB
# freq=noraman b3lyp/gen pseudo=read

Title Card Required

0 1
 C                  4.96920500   -1.91239700    0.83842600
 C                  3.72216400   -2.14000600    1.51555100
 C                  3.35455600   -0.92575900    2.18157900
 C                  5.37977500   -0.55972900    1.10049000
 C                  4.37830200    0.05015400    1.92192100
 Fe                 3.54787100   -0.59270200    0.12067500
 C                  4.70181700    5.01047600   -1.32791200
 C                  4.15604900    5.52317900    0.01270100
 C                  2.89381400    4.67813300    0.21565400
 N                  3.22154900    3.39350200   -0.42582000
 C                  4.42603100    3.50089800   -1.26789300
 C                  2.39074200    2.32455700   -0.36281300
 C                  1.13007900    2.42600800    0.26028700
 C                  0.18729300    1.40397700    0.17048800
 N                  0.42547300    0.21224700   -0.37591200
 C                  1.69930800   -0.01028800   -0.86944900
 C                  2.69901200    1.03909600   -0.94671300
```

H	5.76279600	5.22943300	-1.47753900
H	4.14482700	5.45838100	-2.15889500
H	4.87445800	5.32604800	0.81735700
H	3.93891800	6.59480300	0.01025400
H	2.65050500	4.52659400	1.27264500
H	2.01972900	5.13532900	-0.27010600
H	5.26546500	2.96148800	-0.80846300
H	4.24305400	3.07245600	-2.25639100
H	0.80540900	3.35995600	0.69806600
H	-0.83438300	1.55469000	0.49318800
C	3.53493000	-0.88219200	-1.93757400
H	4.19740800	-1.57423400	-2.44007100
C	3.84888300	0.46665200	-1.61000100
H	4.78280200	0.95567600	-1.83690300
C	2.23874900	-1.19650000	-1.44044600
H	1.72042200	-2.13852300	-1.53833700
C	5.75103900	-2.93600000	0.06486000
H	6.40543900	-3.51603300	0.73058800
H	5.09517700	-3.64756100	-0.44667000
H	6.38966100	-2.47044500	-0.69275200
C	6.66281100	0.07216000	0.64079800
H	7.48793300	-0.16911500	1.32522100
H	6.95496400	-0.27645700	-0.35540600
H	6.58607800	1.16387300	0.60066100
C	4.40827900	1.43840000	2.49469000
H	4.83456800	1.43546300	3.50770600
H	5.01501600	2.11855000	1.88826600
H	3.40162100	1.86330600	2.56521900
C	2.17718800	-0.70526200	3.08846500
H	2.50192400	-0.65286500	4.13694700
H	1.65373400	0.22995000	2.86138100
H	1.44799800	-1.51474200	3.01383200
C	2.99219200	-3.45174500	1.56736100
H	3.09892800	-4.01108900	0.63284100
H	3.38468500	-4.08456800	2.37571100
H	1.92215300	-3.31846400	1.74644400
C	-0.80305900	-0.68821400	-1.09046800
O	-0.59132600	-0.78743700	-2.27859400
C	-1.45230400	-1.81039300	-0.19220800
C	-1.35584200	-3.20294200	-0.81521100
C	-0.86932900	-4.30789900	-0.09902100
C	-1.77959100	-3.42128100	-2.14091800
C	-0.80811300	-5.58042100	-0.67470100
H	-0.54064700	-4.19183600	0.92773700
C	-1.71739300	-4.68913700	-2.71643400
H	-2.13554300	-2.58645800	-2.73265800
C	-1.23229800	-5.77760500	-1.98717400
H	-0.43264600	-6.41572100	-0.08854200
H	-2.04908200	-4.82490000	-3.74266100
H	-1.18844200	-6.76611500	-2.43733500
C	-0.94548100	-1.75898600	1.24911000
H	-1.50355500	-2.45288000	1.88451700
H	0.11589000	-2.01648100	1.29489600
H	-1.06813600	-0.75899000	1.66842800

```
 S                  -3.05495400    1.23211100   -1.60959800
 O                  -2.06383500    2.28262800   -1.90347500
 O                  -3.68981200    0.47339900   -2.70419900
 N                  -2.32774300    0.23739300   -0.49365000
 C                  -2.86671500   -1.12744200   -0.32036700
 H                  -3.33164000   -1.48241400   -1.24651000
 C                  -3.83678300   -1.34932200    0.82262100
 C                  -4.59462100   -2.53007800    0.85355100
 C                  -3.98770200   -0.43006400    1.86792500
 C                  -5.47205000   -2.79022200    1.90650600
 H                  -4.49490700   -3.24843100    0.04313000
 C                  -4.87052000   -0.68498400    2.91926600
 H                  -3.42423100    0.49672900    1.83639000
 C                  -5.61282200   -1.86693000    2.94557900
 H                  -6.05108500   -3.71037900    1.91159700
 H                  -4.98122400    0.04499900    3.71758300
 H                  -6.30104100   -2.06421500    3.76364100
 C                  -5.18490200    3.77544100    0.75631000
 C                  -4.14203500    3.16226900    0.06396400
 C                  -4.40425300    2.03556700   -0.71742400
 C                  -5.70229200    1.53422500   -0.82143700
 C                  -6.73586500    2.15800500   -0.12405200
 C                  -6.49508200    3.28067700    0.68002900
 H                  -4.98234900    4.65906200    1.35784900
 H                  -3.13468500    3.56424600    0.10172500
 H                  -5.89456700    0.67638600   -1.45672900
 H                  -7.74804200    1.76910900   -0.21001200
 C                  -7.61712500    3.93069200    1.45585000
 H                  -7.39281200    4.97766800    1.68582000
 H                  -8.56006600    3.90032000    0.89891100
 H                  -7.78928200    3.41556800    2.41066000

H C O N S 0
6-31G*
****
Fe 0
LANL2DZ
****

Fe 0
LANL2DZ
```

Product D + catalyst

```
%nprocshared=16
%mem=800MB
# opt freq=noraman b3lyp/gen pseudo=read

Title Card Required

0 1
```

C	4.71080200	-2.91420600	-1.26004600
C	3.50026800	-3.39973400	-0.65550900
C	3.39637500	-2.82324500	0.65248500
C	5.36214100	-2.04523500	-0.31677200
C	4.54742400	-1.98544500	0.86057200
Fe	3.46785300	-1.32924300	-0.79369800
C	5.63869900	4.00997800	0.61543900
C	5.48743700	3.73542500	2.11881200
C	4.11529100	3.05713700	2.20640300
N	4.00367000	2.32640900	0.93894500
C	5.00996200	2.76844600	-0.03657400
C	2.95804800	1.48279600	0.68203200
C	1.90071400	1.33671300	1.58791800
C	0.80178500	0.49008900	1.30635300
N	0.64807400	-0.24237200	0.21816300
C	1.67036200	-0.14105100	-0.68725100
C	2.84859200	0.70071800	-0.52990200
H	6.67608700	4.16087200	0.30266900
H	5.07111800	4.90624000	0.33774000
H	6.26914900	3.04627600	2.46027100
H	5.54456800	4.63913200	2.73226100
H	4.03652000	2.36922200	3.05574900
H	3.30667100	3.79862600	2.30445100
H	5.76330600	1.98577300	-0.20448500
H	4.54605200	3.00036500	-0.99989800
H	1.87945900	1.90157400	2.51162400
H	-0.01101900	0.45048800	2.02981200
C	2.98124700	-0.39009000	-2.57662400
H	3.34661000	-0.72546100	-3.53887100
C	3.66654400	0.50885800	-1.70654600
H	4.61511700	0.97301600	-1.92711700
C	1.78922600	-0.83288600	-1.93653000
H	1.07120000	-1.54433900	-2.32157100
C	5.24175500	-3.30999700	-2.60876400
H	5.85749600	-4.21810000	-2.54125100
H	4.43292900	-3.51768500	-3.31690900
H	5.86831900	-2.52525200	-3.04526000
C	6.68992200	-1.37075500	-0.51501800
H	7.51774400	-2.02110000	-0.19878700
H	6.86125300	-1.11446500	-1.56578400
H	6.76378300	-0.44599300	0.06665200
C	4.84639800	-1.22644700	2.12151900
H	5.31372900	-1.87744900	2.87362600
H	5.53107600	-0.39181800	1.93982900
H	3.93380700	-0.81273500	2.56331800
C	2.30727000	-3.07199000	1.65584200
H	2.62448800	-3.80620600	2.40978900
H	2.02580600	-2.15479000	2.18276300
H	1.40311900	-3.45969700	1.17744500
C	2.53441100	-4.36914400	-1.27583000
H	2.52325100	-4.28470200	-2.36739100
H	2.80105100	-5.40672200	-1.03036900
H	1.51244200	-4.20003100	-0.92281600
C	-3.78223900	-0.70816100	-1.09218700

S	-3.35505000	2.21626100	1.31311300
O	-4.63887300	2.69594400	1.81641100
O	-2.26985000	1.81231800	2.20882700
N	-3.66565600	0.83001400	0.35188900
C	-3.12955900	5.08043100	-1.53967800
C	-3.62932400	4.16428300	-0.61724800
C	-2.72751300	3.40589600	0.13390800
C	-1.34756200	3.55102300	-0.02308100
C	-0.87110000	4.47598700	-0.95067000
C	-1.74901900	5.25336000	-1.71951400
H	-3.82480100	5.67047700	-2.13160200
H	-4.69640900	4.02688400	-0.48716800
H	-0.66320200	2.95627600	0.57256900
H	0.20209800	4.59432700	-1.07894300
C	-1.22218600	6.27252800	-2.70190500
H	-1.88221500	6.37003800	-3.57027500
H	-1.14816600	7.26491700	-2.23726000
H	-0.22324200	6.00440500	-3.06099700
C	-2.93065600	-0.46493800	0.24379000
C	-4.74445400	-1.88091000	-1.14018700
C	-4.26217700	-3.19184100	-1.00308700
C	-6.11078100	-1.69009600	-1.38643400
C	-5.12476100	-4.28246800	-1.09883900
H	-3.20766200	-3.36071000	-0.80386300
C	-6.97445300	-2.78413800	-1.48398800
H	-6.49817200	-0.68428800	-1.51371000
C	-6.48630200	-4.08249100	-1.33993000
H	-4.73314500	-5.28956000	-0.98148100
H	-8.03126300	-2.61605600	-1.67452400
H	-7.15911100	-4.93274900	-1.41456500
H	-1.87263100	-0.31166300	0.01088800
C	-3.06634700	-1.38999000	1.42389400
C	-1.97078500	-2.18827700	1.77934400
C	-4.25261800	-1.49052100	2.16277800
C	-2.06532000	-3.08493100	2.84572900
H	-1.03945500	-2.08377400	1.22738400
C	-4.34321600	-2.38146800	3.23117100
H	-5.10172200	-0.86302200	1.90882500
C	-3.25224000	-3.18507900	3.57297800
H	-1.20676500	-3.69504200	3.11510200
H	-5.26715100	-2.44686000	3.79961100
H	-3.32552900	-3.87757900	4.40758700
C	-4.40640500	0.67721200	-0.81669000
O	-5.16990200	1.41832800	-1.39046700
C	-2.90484600	-0.65562500	-2.35673600
H	-2.33267000	-1.58341900	-2.46087900
H	-3.53655300	-0.53880500	-3.24305300
H	-2.19922300	0.18189100	-2.31850900

H C O N S 0
6-31G*
****
Fe 0
LANL2DZ

```
****

Fe 0
LANL2DZ
```

Product D′ + catalyst

```
%nprocshared=16
%mem=1GB
# freq=noraman b3lyp/gen pseudo=read

Title Card Required

0 1
 C                  4.83244100    2.10226200    1.38302800
 C                  3.62529900    2.58878300    0.77226000
 C                  3.52835000    2.01262100   -0.53607200
 C                  5.48864600    1.23457000    0.44185200
 C                  4.68151400    1.17610800   -0.74061700
 Fe                 3.59182000    0.51854600    0.90553800
 C                  5.28478000   -4.91777200   -0.91049300
 C                  5.08463400   -4.55009200   -2.38804000
 C                  3.78649600   -3.73513500   -2.36304400
 N                  3.81893700   -3.06279500   -1.05926700
 C                  4.82573000   -3.65401100   -0.16622500
 C                  2.84485300   -2.17665300   -0.68651600
 C                  1.74549100   -1.91378400   -1.51015000
 C                  0.70863900   -1.04389500   -1.09848000
 N                  0.66233100   -0.37909500    0.04183700
 C                  1.73882300   -0.58440900    0.86378000
 C                  2.86061000   -1.46553600    0.57297700
 H                  6.31515000   -5.19206900   -0.66529500
 H                  4.63929400   -5.76291300   -0.64329400
 H                  5.91460900   -3.92626300   -2.74141700
 H                  5.01486900   -5.42115300   -3.04587200
 H                  3.73075100   -2.99999400   -3.17411900
 H                  2.90255900   -4.38683800   -2.44573100
 H                  5.66596700   -2.96230400   -0.00904600
 H                  4.39325800   -3.88623200    0.81135000
 H                  1.63009400   -2.42245200   -2.45881300
 H                 -0.14645600   -0.92604000   -1.76250400
 C                  3.19154300   -0.50629500    2.66271300
 H                  3.63949600   -0.24614900    3.61330200
 C                  3.76960600   -1.37898000    1.69349300
 H                  4.70826100   -1.89684900    1.81777200
 C                  1.97859100    0.02441700    2.13934300
 H                  1.32029300    0.73626400    2.61893500
 C                  5.35542100    2.49660700    2.73530400
 H                  5.96914500    3.40660800    2.67370900
 H                  4.54230600    2.69983700    3.43998700
 H                  5.98167500    1.71240500    3.17310100
 C                  6.81292600    0.55513100    0.64669500
```

H	7.64458000	1.19954400	0.32827800
H	6.98098500	0.30385200	1.69916400
H	6.88246100	-0.37361200	0.07093100
C	4.99128600	0.41915300	-2.00019600
H	5.47966200	1.06659200	-2.74210700
H	5.66145100	-0.42534700	-1.81079100
H	4.08098500	0.01929300	-2.45892100
C	2.43646800	2.25388300	-1.53766300
H	2.70953100	3.05628100	-2.23819100
H	2.22928500	1.35506100	-2.12702200
H	1.50082000	2.53826900	-1.04738800
C	2.65292700	3.55583900	1.38578100
H	2.63635700	3.47314000	2.47746200
H	2.91594600	4.59465300	1.14021200
H	1.63391900	3.38038700	1.02599000
C	-2.83126500	-1.83315100	-0.57065500
O	-2.34234600	-2.67388100	-1.26285000
C	-3.43895700	-1.75849700	0.84594200
C	-4.63469000	-2.68748400	1.03320800
C	-4.83125900	-3.41532900	2.21357000
C	-5.59019400	-2.81322000	0.00893900
C	-5.94869000	-4.23973600	2.37038800
H	-4.11360800	-3.34604100	3.02370500
C	-6.70268200	-3.63758300	0.16301000
H	-5.46834400	-2.26395800	-0.92173400
C	-6.88808100	-4.35538900	1.34718800
H	-6.07920000	-4.79273100	3.29701300
H	-7.42353200	-3.72040000	-0.64586100
H	-7.75513800	-4.99916600	1.46817400
C	-2.34717000	-1.89592300	1.91210300
H	-2.71238900	-1.57561500	2.89307100
H	-2.02435000	-2.93965300	1.97838100
H	-1.47422800	-1.28993500	1.65888600
S	-3.50853900	0.33663000	-2.27426900
O	-2.63435200	-0.27134900	-3.27622300
O	-4.97167300	0.28089800	-2.39988200
N	-3.08237800	-0.38997900	-0.78771200
C	-3.85608100	-0.26001300	0.48173900
H	-4.93007100	-0.22008400	0.27967000
C	-3.46503300	0.87198200	1.39654000
C	-4.43728100	1.40868800	2.25218200
C	-2.15981900	1.38405600	1.44116500
C	-4.11654400	2.43290000	3.14421700
H	-5.45387700	1.02241000	2.21676700
C	-1.84471200	2.41454300	2.32816500
H	-1.39047100	0.97544600	0.79057300
C	-2.81686300	2.94093500	3.18245100
H	-4.88260500	2.83789300	3.80035700
H	-0.83138100	2.80861600	2.34957900
H	-2.56464300	3.74374200	3.87059700
C	-1.36288600	3.76149400	-2.03724700
C	-1.70766000	2.42168400	-2.18779100
C	-3.04704200	2.04935300	-2.04667700
C	-4.03510300	2.99472600	-1.77258100

```
 C                  -3.66887500    4.33215700   -1.62519300
 C                  -2.33289400    4.73501600   -1.74862200
 H                  -0.32363800    4.05907100   -2.15296800
 H                  -0.95406400    1.67641000   -2.41787500
 H                  -5.07102900    2.68439200   -1.69172500
 H                  -4.43509000    5.07408300   -1.41515400
 C                  -1.93731300    6.17996900   -1.55891200
 H                  -1.14132500    6.47211500   -2.25245500
 H                  -2.78712800    6.85259800   -1.71205100
 H                  -1.56096400    6.35235600   -0.54183800

H C O N S 0
6-31G*
****
Fe 0
LANL2DZ
****

Fe 0
LANL2DZ
```

Optimized molecular structure of reactants, intermediates, and products for the ketene-first mechanism of nucleophile-catalyzed Staudinger reaction involving N-Tf imine.

E. PPY-catalyst + ketene + N-Ts imine

```
%nprocshared=16
%mem=800MB
# opt=modredundant freq=noraman b3lyp/gen pseudo=read

Title Card Required

0 1
 C                  -3.15575900   -4.14241500    1.00972200
 C                  -1.93775200   -4.52321800    0.34833300
 C                  -1.96111200   -3.97276900   -0.97538500
 C                  -3.93950100   -3.36694900    0.08630200
 C                  -3.20039000   -3.25992100   -1.13666000
 Fe                 -2.10295200   -2.45699500    0.44070600
 C                  -4.85288300    2.58389900   -1.03025100
 C                  -4.71142600    2.30461500   -2.53347500
 C                  -3.27949200    1.77037900   -2.64593300
 N                  -3.06964500    1.05952700   -1.37683800
 C                  -4.08879700    1.42207800   -0.37663900
 C                  -1.93929500    0.34116200   -1.13520000
 C                  -0.90183700    0.26952600   -2.07935700
 C                   0.28452800   -0.44805700   -1.81179300
 N                   0.56280100   -1.11911800   -0.70771300
 C                  -0.43794100   -1.09718900    0.23082600
 C                  -1.70298400   -0.38768900    0.09317400
```

H	-5.89305300	2.63361000	-0.69408700
H	-4.36285500	3.53042200	-0.78106300
H	-5.42659400	1.53593600	-2.85203100
H	-4.86963700	3.19131100	-3.15400300
H	-3.14665300	1.08888300	-3.49392600
H	-2.55653200	2.59252100	-2.74886100
H	-4.75412200	0.56910900	-0.18006600
H	-3.61389900	1.72123300	0.55973000
H	-0.97447600	0.80132300	-3.01971000
H	1.06621800	-0.43707500	-2.57249900
C	-1.64994600	-1.41145400	2.17530000
H	-1.94275100	-1.74597800	3.16225500
C	-2.45178400	-0.61665000	1.30629200
H	-3.42955500	-0.23379900	1.55241500
C	-0.44258300	-1.75506400	1.50373100
H	0.35599300	-2.37576400	1.88547000
C	-3.57196600	-4.54809700	2.39513800
H	-4.11515200	-5.50381300	2.38298700
H	-2.70857100	-4.67337400	3.05665700
H	-4.23283300	-3.80482100	2.85306500
C	-5.31452000	-2.81956600	0.34472900
H	-6.09067400	-3.54693000	0.06729200
H	-5.45966900	-2.57682900	1.40246500
H	-5.50343000	-1.90781100	-0.23172500
C	-3.64984600	-2.56601800	-2.39052800
H	-4.11080400	-3.27569500	-3.09192800
H	-4.38928300	-1.78770000	-2.17715900
H	-2.81070100	-2.08842500	-2.90680700
C	-0.90360500	-4.13872300	-2.02889100
H	-1.13123600	-4.98669900	-2.69047900
H	-0.81632600	-3.24455600	-2.65410200
H	0.08024300	-4.31785800	-1.58546600
C	-0.85491900	-5.38677600	0.93018400
H	-0.77614500	-5.25988700	2.01491600
H	-1.05221700	-6.45123400	0.73927100
H	0.12310300	-5.15305200	0.49802300
S	-1.03236700	4.08764600	0.46751400
O	-1.87165600	4.54886400	-0.64138300
O	-1.41916800	2.90311400	1.24329000
C	-0.92378000	5.50234100	1.68342700
F	-0.03853100	5.21195500	2.63810000
F	-2.13051500	5.66364000	2.23059000
F	-0.56229200	6.62387400	1.06435000
N	0.57506000	4.08641300	-0.02673000
C	1.17598300	2.93872600	-0.04490500
H	0.66705300	2.02220400	0.26981400
C	2.55909500	2.81360000	-0.47732000
C	3.14559000	1.53536900	-0.51377400
C	3.31408000	3.94367200	-0.85085000
C	4.47179100	1.39003900	-0.91822100
H	2.55720500	0.66176600	-0.24232300
C	4.63593200	3.79191100	-1.24705200
H	2.84641500	4.92235900	-0.82078300
C	5.21433700	2.51574400	-1.28050000

```
 H                   4.92345200    0.40479400   -0.95204000
 H                   5.22034500    4.66228900   -1.53180500
 H                   6.24893300    2.39761500   -1.59122400
 C                   4.32571900   -2.40634500    2.35414800
 O                   4.31243600   -2.21650500    3.51227500
 C                   4.32400800   -2.63306000    1.04847000
 C                   5.43756200   -2.15770800    0.20564700
 C                   5.40096800   -2.34765500   -1.18803500
 C                   6.56379000   -1.50692400    0.75202400
 C                   6.44853100   -1.90518200   -1.99930800
 H                   4.54761600   -2.83766200   -1.64480100
 C                   7.60247300   -1.06499700   -0.06035400
 H                   6.62581800   -1.34709200    1.82640200
 C                   7.55440800   -1.26071100   -1.44520400
 H                   6.39297400   -2.06686100   -3.07284500
 H                   8.45732100   -0.56783300    0.39091100
 H                   8.36901900   -0.92002200   -2.07804600
 C                   3.13418100   -3.39014700    0.47453700
 H                   2.51446400   -2.74287600   -0.15860000
 H                   3.47974200   -4.25049500   -0.11199500
 H                   2.49237200   -3.77712300    1.27217900

B 15 75 F
B 62 77 F


H C O N F S 0
6-31G*
****
Fe 0
LANL2DZ
****

Fe 0
LANL2DZ
```

TS$_{E-F}$

```
%nprocshared=16
%mem=800MB
# opt=(modredundant,ts,calcfc,noeigen) freq=noraman b3lyp/gen pseudo=read

Title Card Required

0 1
 C                   5.33983100   -1.51025800   -1.07325600
 C                   4.35712900   -2.39134900   -0.50499200
 C                   4.07372400   -1.94025800    0.82543200
 C                   5.67630000   -0.52204700   -0.08455400
 C                   4.89169800   -0.78508800    1.08485200
 Fe                  3.65347600   -0.43147000   -0.55087300
```

C	3.54281600	5.03596000	1.73580900
C	3.38903700	4.49701400	3.16503100
C	2.39057300	3.34652700	2.99278400
N	2.66938800	2.83489000	1.64042200
C	3.51864900	3.76474000	0.87482600
C	2.04679700	1.73627600	1.14384300
C	1.04248000	1.07953100	1.88445900
C	0.29965900	0.03640600	1.32942400
N	0.51351600	-0.48806600	0.13114700
C	1.54802300	0.03735300	-0.60951400
C	2.32691100	1.18302000	-0.16125500
H	4.45696200	5.61723000	1.58618900
H	2.69074200	5.67601400	1.47931900
H	4.34747500	4.11146500	3.53253600
H	3.03373200	5.24816300	3.87567100
H	2.52370700	2.55451800	3.73758400
H	1.35206900	3.70226800	3.06055200
H	4.52867600	3.35072200	0.75050300
H	3.09634100	3.94319500	-0.11694700
H	0.75612600	1.43483600	2.86510300
H	-0.54050400	-0.37815900	1.88006400
C	3.04357400	0.56092700	-2.27623000
H	3.59320900	0.53278800	-3.20767400
C	3.27010500	1.48026200	-1.21448500
H	4.00991200	2.26435000	-1.22767100
C	2.02764300	-0.35848300	-1.89292600
H	1.64363800	-1.16762900	-2.49517100
C	5.96260700	-1.65063800	-2.43319100
H	6.82709600	-2.32848200	-2.40163400
H	5.25523100	-2.05915200	-3.16205800
H	6.31783300	-0.68978200	-2.81901600
C	6.70858200	0.55904000	-0.23578000
H	7.70554400	0.19592700	0.05026400
H	6.77620600	0.91449300	-1.26920000
H	6.48521400	1.42375700	0.39818400
C	4.95775400	-0.03978100	2.38724000
H	5.71291100	-0.47967400	3.05344700
H	5.22344200	1.01241900	2.24243900
H	3.99951300	-0.07045800	2.91547600
C	3.13766900	-2.58046800	1.80967900
H	3.67504700	-3.27671000	2.46876400
H	2.65254200	-1.83405200	2.44670700
H	2.34258400	-3.14285400	1.31403700
C	3.77709000	-3.60229600	-1.17831500
H	3.65488200	-3.44923400	-2.25532900
H	4.42881600	-4.47669700	-1.04321100
H	2.79718100	-3.86009700	-0.76687900
S	-2.07397900	0.26273300	-2.16768100
O	-1.01310700	-0.03024700	-3.15117800
O	-3.47371900	0.29929900	-2.60945100
C	-1.75700700	2.02582300	-1.63751600
F	-2.62709400	2.40503700	-0.68919400
F	-1.88361700	2.84384600	-2.68744100
F	-0.51328400	2.16745700	-1.13968600

```
 N                  -1.95321600   -0.55630200   -0.77758600
 C                  -0.89974900   -1.39759100   -0.63973700
 H                  -0.34896200   -1.68682200   -1.53634400
 C                  -1.11323700   -2.53008000    0.32124900
 C                  -0.28423900   -3.65831000    0.26562600
 C                  -2.16579400   -2.50089600    1.24564400
 C                  -0.48773100   -4.73262200    1.13113300
 H                   0.51307100   -3.69835400   -0.47308300
 C                  -2.36334200   -3.57181400    2.11933500
 H                  -2.84199500   -1.65234700    1.24268900
 C                  -1.52442900   -4.68701500    2.06770600
 H                   0.15434500   -5.60739700    1.06983900
 H                  -3.18645600   -3.53962100    2.82803300
 H                  -1.68581600   -5.52402200    2.74189100
 C                  -7.94194100    2.28003600    0.45061100
 O                  -8.72506800    3.01217900    0.93382700
 C                  -7.05386500    1.47036900   -0.10452300
 C                  -6.90540000    0.07760500    0.36816100
 C                  -5.92306700   -0.75574000   -0.19585600
 C                  -7.73128900   -0.45546200    1.37899800
 C                  -5.78080500   -2.07573100    0.23906200
 H                  -5.26241600   -0.38491900   -0.97265200
 C                  -7.58131400   -1.76975400    1.80881400
 H                  -8.50249400    0.16600300    1.83040300
 C                  -6.60337200   -2.59246700    1.24020100
 H                  -5.01343500   -2.69692900   -0.21466300
 H                  -8.23436800   -2.15468900    2.58842900
 H                  -6.49042900   -3.62167100    1.57093900
 C                  -6.20878700    2.03495800   -1.23761000
 H                  -5.14459900    2.02366400   -0.98287800
 H                  -6.33394600    1.43896300   -2.14854000
 H                  -6.49176500    3.06673100   -1.46815300

H C O N F S 0
6-31G*
****
Fe 0
LANL2DZ
****

Fe 0
LANL2DZ
```

Intermediate F

```
%nprocshared=16
%mem=800MB
# opt=(modredundant) freq=noraman b3lyp/gen pseudo=read

Title Card Required
```

0 1

C	-5.25298600	-1.26260600	0.01776500
C	-4.32013800	-1.95043000	-0.83182200
C	-3.79495800	-1.00362400	-1.77094900
C	-5.31690800	0.10837900	-0.40966000
C	-4.41406800	0.26883800	-1.50997800
Fe	-3.38664500	-0.38341200	0.18191900
C	-2.21913900	5.40912400	0.65024300
C	-1.88718500	5.52889700	-0.84392700
C	-1.08437600	4.25336200	-1.12294900
N	-1.65150400	3.26503900	-0.18592000
C	-2.50264700	3.91105800	0.83002800
C	-1.27230100	1.96703300	-0.19375700
C	-0.24752200	1.52615000	-1.06228200
C	0.27693500	0.24643600	-0.97078900
N	-0.19182000	-0.70131500	-0.15375300
C	-1.28198600	-0.38316100	0.63883600
C	-1.82857200	0.96376500	0.68245800
H	-3.06658700	6.02775200	0.95841800
H	-1.35236300	5.69916200	1.25514500
H	-2.80741700	5.53283300	-1.44014000
H	-1.32266100	6.43204000	-1.09080600
H	-1.18853100	3.90458900	-2.15552700
H	-0.01363300	4.39584800	-0.92094600
H	-3.56095000	3.68172200	0.64496100
H	-2.24185600	3.55548700	1.82936100
H	0.24937300	2.21094800	-1.73377600
H	1.14647900	-0.02655700	-1.55690100
C	-2.94568800	-0.36340000	2.21859500
H	-3.63652200	-0.68989400	2.98423900
C	-2.88326000	0.94568100	1.66989000
H	-3.51553600	1.76758100	1.96495100
C	-2.00108200	-1.19779200	1.55828500
H	-1.80932400	-2.23561400	1.78156400
C	-6.07827500	-1.88609600	1.10720600
H	-7.03107000	-2.26361500	0.71045100
H	-5.56322800	-2.73153400	1.57415100
H	-6.31745900	-1.16688800	1.89720200
C	-6.21040000	1.16907700	0.16734900
H	-7.18988700	1.17479200	-0.33066000
H	-6.39015500	1.01124100	1.23571300
H	-5.77987500	2.16895700	0.04794600
C	-4.19943900	1.52241900	-2.30854700
H	-4.86965500	1.55257400	-3.17885300
H	-4.39541600	2.42201600	-1.71613000
H	-3.17386600	1.58856600	-2.68507300
C	-2.82711700	-1.28577200	-2.88429600
H	-3.35998100	-1.52331900	-3.81569900
H	-2.18313900	-0.42416900	-3.08791000
H	-2.17153000	-2.12934600	-2.65559000
C	-4.01244200	-3.41971200	-0.77721600
H	-4.03332000	-3.80259900	0.24804200
H	-4.74853800	-3.99419200	-1.35660600

H	-3.02640600	-3.64305100	-1.19362700
S	1.88566500	-1.60889300	2.46969800
O	0.69805600	-2.28436800	3.04214200
O	3.21889600	-1.96771800	2.97579500
C	1.72495700	0.15759700	3.05859700
F	2.72200100	0.91647800	2.57365200
F	1.75380400	0.22336100	4.39534900
F	0.55857800	0.70621300	2.64432900
N	1.88650900	-1.48907100	0.88317300
C	0.74516900	-1.93200700	0.18952100
H	0.08238700	-2.57615100	0.77432400
C	1.10144400	-2.62746300	-1.11828500
C	0.16398700	-3.45584200	-1.74766700
C	2.37357000	-2.48925500	-1.68492900
C	0.47745700	-4.11515100	-2.93658500
H	-0.81063500	-3.60317400	-1.28795900
C	2.68578200	-3.14015500	-2.88074900
H	3.11539900	-1.89790100	-1.15839200
C	1.74006400	-3.95035600	-3.51183400
H	-0.25640900	-4.76535400	-3.40601400
H	3.67756100	-3.02561900	-3.31037500
H	1.98937200	-4.46462400	-4.43635600
C	3.94548200	2.09717000	-2.05319800
O	2.78202100	2.23070700	-2.18870400
C	5.25469700	1.96613900	-1.93203300
C	5.85174500	1.09066000	-0.89596500
C	7.24480900	0.91799300	-0.84556900
C	5.05943500	0.41226900	0.05112800
C	7.82317000	0.08904000	0.11843500
H	7.88560900	1.42740000	-1.55851600
C	5.63755600	-0.41822500	1.00746900
H	3.97766000	0.52607500	0.05429400
C	7.02667000	-0.58253600	1.04443500
H	8.90398400	-0.02881800	0.13809600
H	4.99842800	-0.93394300	1.71883500
H	7.47884200	-1.22937500	1.79116000
C	6.11097300	2.74406300	-2.91992700
H	6.79095600	3.42273600	-2.39067600
H	6.71780900	2.06360300	-3.52971300
H	5.50022000	3.34540300	-3.59995400

H C O N F S 0
6-31G*
****
Fe 0
LANL2DZ
****

Fe 0
LANL2DZ

TS$_{F\text{-}G'}$

```
%nprocshared=16
%mem=800MB
# freq=noraman b3lyp/gen pseudo=read

Title Card Required

0 1
 C                  3.47052100   -3.22881400   -0.84283200
 C                  2.33403400   -3.30368100    0.03314300
 C                  2.59916500   -2.47360100    1.16997600
 C                  4.44667500   -2.36466800   -0.23566200
 C                  3.90524000   -1.89421400    1.00379300
 Fe                 2.67541400   -1.33613100   -0.58319600
 C                  5.51158300    3.73324900    0.39533500
 C                  5.24415100    3.65115500    1.90481000
 C                  3.78845000    3.17618900    1.97299900
 N                  3.63091200    2.33941100    0.76752800
 C                  4.76168500    2.51560900   -0.16285100
 C                  2.50620100    1.63153600    0.52476900
 C                  1.38491600    1.74925000    1.37930600
 C                  0.15472500    1.19740000    1.05790100
 N                 -0.03682500    0.39652200   -0.00067600
 C                  1.05839300    0.09243700   -0.79311200
 C                  2.33529500    0.76129100   -0.61545200
 H                  6.57485100    3.71246700    0.14091700
 H                  5.08475800    4.65508900   -0.01614700
 H                  5.90449900    2.90968700    2.36978400
 H                  5.38981600    4.60292700    2.42245600
 H                  3.57466500    2.59201100    2.87386600
 H                  3.08625200    4.02083000    1.94184200
 H                  5.39927100    1.62124700   -0.16212200
 H                  4.39755700    2.68066700   -1.17951500
 H                  1.41346200    2.40533200    2.23788400
 H                 -0.73435800    1.46320500    1.62824600
 C                  2.43462900   -0.58227500   -2.50306900
 H                  2.80065800   -1.08880400   -3.38596100
 C                  3.18585300    0.31463800   -1.69618400
 H                  4.20785400    0.60115000   -1.88370500
 C                  1.14368000   -0.76408500   -1.92656500
 H                  0.36649200   -1.41721500   -2.29434800
 C                  3.64397100   -3.98414200   -2.12977800
 H                  4.10283000   -4.96593800   -1.94857700
 H                  2.68618800   -4.16108500   -2.62887400
 H                  4.29039600   -3.44826400   -2.83228900
 C                  5.80803000   -2.05473700   -0.78988000
 H                  6.53604200   -2.82375700   -0.49726400
 H                  5.80089900   -2.01368700   -1.88398600
 H                  6.18721200   -1.09447500   -0.42481600
 C                  4.59564600   -1.01587600    2.00809700
 H                  5.08687400   -1.62015300    2.78322800
 H                  5.36775300   -0.39269500    1.54562200
 H                  3.88704200   -0.35237000    2.51420200
 C                  1.72361400   -2.30344100    2.37768800
```

H	2.04545000	-2.97574700	3.18546800
H	1.76410800	-1.28138700	2.76915600
H	0.67908400	-2.53147100	2.15494900
C	1.10956700	-4.14459200	-0.18492400
H	0.90501700	-4.29053700	-1.25014300
H	1.23953900	-5.14022100	0.26174800
H	0.21931800	-3.69557300	0.26089100
S	-2.55691500	2.21731100	-1.32944500
O	-2.21441000	1.71542000	-2.67233100
O	-3.78853600	2.96974400	-1.09449000
C	-1.22976000	3.51286400	-1.02771100
F	-1.18636800	3.87929100	0.25789700
F	-1.47604900	4.58978200	-1.77798900
F	-0.01206000	3.03392800	-1.36879500
N	-2.40485900	1.06196700	-0.19258800
C	-1.46204800	0.02057800	-0.47488300
H	-1.33587400	-0.07747100	-1.55591400
C	-1.86712600	-1.33348100	0.08516600
C	-2.35764700	-2.29686200	-0.80262800
C	-1.79944000	-1.63873600	1.45008900
C	-2.76305000	-3.55128400	-0.34217000
H	-2.44841100	-2.05453800	-1.85872800
C	-2.20226900	-2.89285100	1.91202900
H	-1.44438200	-0.89728600	2.15870900
C	-2.68018700	-3.85407400	1.01675600
H	-3.15149300	-4.28334900	-1.04434000
H	-2.15052500	-3.11575100	2.97453300
H	-2.99656700	-4.82802000	1.38036900
C	-3.28534300	1.30333600	1.56758800
O	-2.55054800	2.02636400	2.20218400
C	-4.40409300	0.54842400	1.67280200
C	-5.12288200	-0.26545700	0.67364200
C	-5.64628700	-1.51766200	1.06014800
C	-5.41042300	0.17299700	-0.63398400
C	-6.38261200	-2.30739300	0.17779400
H	-5.46099800	-1.88561000	2.06487500
C	-6.13730700	-0.62245400	-1.51961800
H	-5.09519300	1.16158400	-0.93977900
C	-6.62684800	-1.86990700	-1.12512000
H	-6.76501600	-3.26982400	0.51172600
H	-6.34199800	-0.24865600	-2.52045000
H	-7.20387700	-2.48140400	-1.81474300
C	-4.94686600	0.53103600	3.10380200
H	-6.04099100	0.60831000	3.09901200
H	-4.68794200	-0.39408800	3.64179500
H	-4.54775900	1.36656400	3.68460500

H C O N F S 0
6-31G*
****
Fe 0
LANL2DZ
****

Fe 0
LANL2DZ

TS$_{F-G}$

```
%nprocshared=8
%mem=800MB
# freq=noraman b3lyp/gen pseudo=read

Title Card Required

0 1
 C                  4.14610500   -2.88401000   -0.33703100
 C                  2.83784200   -3.12936800    0.20334600
 C                  2.67934800   -2.30626000    1.36531400
 C                  4.80290500   -1.91977300    0.50329200
 C                  3.89520900   -1.56021200    1.55096500
 Fe                 3.06729000   -1.11593500   -0.30941800
 C                  4.96870300    4.23254600    1.27422300
 C                  4.38136100    4.07910300    2.68440600
 C                  3.02108700    3.42260800    2.42367800
 N                  3.24633600    2.60458200    1.21600200
 C                  4.52453100    2.94611600    0.56403700
 C                  2.30462100    1.77005100    0.72543400
 C                  1.01809100    1.71151800    1.31110100
 C                 -0.02888500    1.02509900    0.71568900
 N                  0.12035800    0.25563800   -0.37133100
 C                  1.39405400    0.11467400   -0.89732000
 C                  2.50592300    0.92315000   -0.42783000
 H                  6.05568000    4.35106600    1.26733600
 H                  4.53164800    5.10440200    0.77417000
 H                  5.01002600    3.41590900    3.29021700
 H                  4.28312400    5.02844500    3.21739600
 H                  2.68808500    2.79340000    3.25532300
 H                  2.24077800    4.17199400    2.23145800
 H                  5.25433200    2.13827900    0.70962000
 H                  4.37914700    3.09106100   -0.50899000
 H                  0.77384800    2.32369900    2.16787100
 H                 -1.04507700    1.13113100    1.08518600
 C                  3.19852600   -0.35075900   -2.23834700
 H                  3.81869200   -0.79031900   -3.00802200
 C                  3.62956900    0.60815400   -1.28169500
 H                  4.62610600    1.01440400   -1.22461700
 C                  1.83914100   -0.69464300   -1.97973000
 H                  1.25079200   -1.42634200   -2.51217700
 C                  4.75364400   -3.57336100   -1.52558900
 H                  5.29220400   -4.48066200   -1.21895700
 H                  3.99272800   -3.87740400   -2.25109800
 H                  5.47052700   -2.93011900   -2.04609000
 C                  6.21308600   -1.42882300    0.33854400
 H                  6.92152200   -2.09153400    0.85439500
 H                  6.51093100   -1.39312300   -0.71443800
 H                  6.34728000   -0.42531400    0.75604700
```

C	4.17595100	-0.62896900	2.69524600
H	4.52636500	-1.18553400	3.57537600
H	4.95052100	0.10317400	2.44501600
H	3.27784500	-0.07912700	2.99446000
C	1.49473500	-2.27993400	2.28756500
H	1.65360900	-2.95327500	3.14151100
H	1.31575900	-1.27793100	2.69139200
H	0.58104500	-2.60006600	1.78169300
C	1.84157000	-4.12015400	-0.32606900
H	1.91946400	-4.23021400	-1.41229600
H	2.01287600	-5.11184100	0.11551800
H	0.81305800	-3.83110800	-0.09795600
S	-2.39888500	1.82310600	-2.12083900
O	-1.73099800	1.49438200	-3.39384900
O	-3.77181600	2.32003300	-2.09790600
C	-1.40233600	3.28091100	-1.49225300
F	-1.69237300	3.54605400	-0.21034900
F	-1.65424400	4.36819300	-2.22488300
F	-0.07873900	3.01613400	-1.57315500
N	-2.22516600	0.64741000	-1.01326000
C	-1.10722200	-0.22842800	-1.18453100
H	-0.74103800	-0.18041200	-2.21411300
C	-1.44035900	-1.67681000	-0.86213500
C	-1.48769700	-2.59733200	-1.91617000
C	-1.76803000	-2.10782600	0.43086600
C	-1.84944000	-3.92730300	-1.68877800
H	-1.26445000	-2.26507300	-2.92783700
C	-2.12729100	-3.43715200	0.65914900
H	-1.77184100	-1.40273300	1.25589600
C	-2.16763100	-4.35066600	-0.39798900
H	-1.88925700	-4.62579700	-2.52024900
H	-2.39425300	-3.75441100	1.66343100
H	-2.45602500	-5.38243100	-0.21628000
C	-3.57062800	0.46170700	0.53914200
O	-2.96355100	0.88470800	1.49058900
C	-4.70729300	-0.11177400	0.08663200
C	-5.77945400	-0.30030100	1.09868800
C	-7.05639900	-0.74209700	0.68510300
C	-5.60614000	-0.07341900	2.48368400
C	-8.09085800	-0.94977200	1.59724300
H	-7.25100100	-0.91694200	-0.36675700
C	-6.64214400	-0.28331500	3.38956900
H	-4.64793000	0.27352500	2.85186300
C	-7.89606600	-0.72538700	2.95962200
H	-9.05858800	-1.28712300	1.23215000
H	-6.46481700	-0.09771600	4.44695900
H	-8.70219100	-0.88696800	3.67056900
C	-4.97556200	-0.54459900	-1.33170100
H	-5.37893300	-1.56547000	-1.34944400
H	-5.70212900	0.10985000	-1.83016000
H	-4.06480300	-0.53568900	-1.92438000

H C O N F S 0
6-31G*

```
****
Fe 0
LANL2DZ
****

Fe 0
LANL2DZ
```

Intermediate G

```
%nprocshared=16
%mem=800MB
# freq=noraman b3lyp/gen pseudo=read

Title Card Required

0 1
 C                  4.14939400   -2.83658100   -0.39023700
 C                  2.85895800   -3.11563700    0.17555600
 C                  2.69814200   -2.29244400    1.33748500
 C                  4.79454000   -1.85167300    0.43455800
 C                  3.89648100   -1.51300700    1.49761400
 Fe                 3.02295300   -1.09953700   -0.34985400
 C                  4.91211700    4.29454600    1.10136900
 C                  4.39649600    4.13662400    2.53881300
 C                  3.03968600    3.44971500    2.34725600
 N                  3.22276200    2.63214200    1.13175500
 C                  4.46401700    2.99355900    0.42128700
 C                  2.27265400    1.78130600    0.68780600
 C                  1.01468700    1.70539300    1.32976100
 C                 -0.05193600    1.00550100    0.78858400
 N                  0.06186000    0.23950400   -0.31083800
 C                  1.31675200    0.10672200   -0.89057800
 C                  2.43572800    0.93104500   -0.46949100
 H                  5.99454000    4.43743800    1.04147700
 H                  4.43171100    5.15283000    0.61773700
 H                  5.06801100    3.49044000    3.11636200
 H                  4.30375400    5.08656900    3.07169500
 H                  2.76141800    2.81641100    3.19563400
 H                  2.23481300    4.18138300    2.19173700
 H                  5.21671300    2.20262300    0.54167700
 H                  4.26911300    3.12539500   -0.64559000
 H                  0.80228600    2.31535600    2.19683900
 H                 -1.07201700    1.08513600    1.19317300
 C                  3.07861500   -0.35334800   -2.29049000
 H                  3.67527400   -0.79453900   -3.07757800
 C                  3.53197900    0.61979600   -1.35941800
 H                  4.52621100    1.03532300   -1.33811700
 C                  1.73238700   -0.70814400   -1.98066800
 H                  1.13577100   -1.45144000   -2.48712500
 C                  4.75497900   -3.51584900   -1.58569300
 H                  5.31515200   -4.41177100   -1.28433700
 H                  3.99118900   -3.83662900   -2.30098600
```

H	5.45274900	-2.85982200	-2.11629000
C	6.18752000	-1.32287100	0.24232700
H	6.92571800	-1.97905100	0.72356200
H	6.45558100	-1.25561900	-0.81724700
H	6.30846600	-0.32639800	0.67991100
C	4.17443000	-0.56724700	2.63050600
H	4.59119700	-1.10335200	3.49418100
H	4.89608000	0.20593600	2.34791500
H	3.26132300	-0.06674000	2.96782500
C	1.52906400	-2.29635000	2.27938200
H	1.70875100	-2.98484800	3.11694900
H	1.34725800	-1.30462700	2.70613200
H	0.60922400	-2.61482600	1.78346600
C	1.88284200	-4.13779900	-0.33007800
H	1.94024000	-4.24793000	-1.41764600
H	2.09488900	-5.12258600	0.10923900
H	0.85123300	-3.88053100	-0.07981600
S	-2.46244100	1.80189600	-2.04992100
O	-1.68703600	1.45268400	-3.24705000
O	-3.84434300	2.24903800	-2.11950200
C	-1.54870900	3.27927500	-1.33780600
F	-1.90175800	3.49404200	-0.07036700
F	-1.83546300	4.35931100	-2.06652100
F	-0.21863300	3.07289300	-1.38812700
N	-2.31700500	0.57691500	-0.93519800
C	-1.16366900	-0.29877100	-1.05197100
H	-0.84761000	-0.27325600	-2.09469300
C	-1.48299900	-1.73909300	-0.68643700
C	-1.48742400	-2.69070000	-1.71355000
C	-1.82963100	-2.13346800	0.61429900
C	-1.81858000	-4.02228600	-1.45194400
H	-1.25494000	-2.38557100	-2.73175800
C	-2.15546000	-3.46541400	0.87441400
H	-1.88839300	-1.39457500	1.40757200
C	-2.14853100	-4.41234600	-0.15379900
H	-1.82536100	-4.74657700	-2.26180400
H	-2.44000000	-3.75737700	1.88135000
H	-2.41314000	-5.44539800	0.05515500
C	-3.32247500	0.44053600	0.25326400
O	-2.82802100	0.81923300	1.35258900
C	-4.53918700	-0.12845400	-0.05927300
C	-5.57481600	-0.29113300	0.98041100
C	-6.86201300	-0.76849600	0.62992900
C	-5.37072600	-0.00029700	2.35348300
C	-7.86784500	-0.95097300	1.57754300
H	-7.09116700	-0.99199600	-0.40582400
C	-6.37974600	-0.18601500	3.29523800
H	-4.40678000	0.37923500	2.66210300
C	-7.63849700	-0.66514100	2.92362700
H	-8.84065400	-1.31708000	1.25502300
H	-6.17671200	0.04999500	4.33847300
H	-8.42192000	-0.80762900	3.66412000
C	-4.88543900	-0.61708400	-1.44835800
H	-5.27927100	-1.64212300	-1.41343000

```
 H                  -5.64849200    0.00560100   -1.93523000
 H                  -4.01805400   -0.63391400   -2.10838000

H C O N F S 0
6-31G*
****
Fe 0
LANL2DZ
****

Fe 0
LANL2DZ
```

Intermediate G′

```
%nprocshared=16
%mem=800MB
# freq=noraman b3lyp/gen pseudo=read

Title Card Required

0 1
 C                   3.47128300   -3.22814100   -0.87655900
 C                   2.32610800   -3.33656200   -0.01598300
 C                   2.55895600   -2.51435800    1.13410900
 C                   4.42136600   -2.35129500   -0.24702800
 C                   3.85548300   -1.90820300    0.99160900
 Fe                  2.63281800   -1.35523300   -0.60422300
 C                   5.51193000    3.71396600    0.26224700
 C                   5.31263800    3.61566500    1.78127300
 C                   3.86240900    3.13647000    1.90926000
 N                   3.65352800    2.31100100    0.70354300
 C                   4.74340700    2.49780600   -0.27291700
 C                   2.51842900    1.60718500    0.50170800
 C                   1.43061100    1.72301600    1.39864900
 C                   0.18436500    1.18366000    1.12307800
 N                  -0.04801900    0.39252400    0.06036000
 C                   1.01843000    0.07996600   -0.77184900
 C                   2.30338600    0.74167200   -0.63512100
 H                   6.56272200    3.70133700   -0.03999400
 H                   5.06207100    4.63733900   -0.12023700
 H                   5.99455400    2.87087400    2.20828000
 H                   5.47966400    4.56228900    2.30189400
 H                   3.68915400    2.54276800    2.81248200
 H                   3.15817200    3.97998900    1.91742800
 H                   5.38403900    1.60630800   -0.30563400
 H                   4.33588800    2.66788400   -1.27224000
 H                   1.49612600    2.37298700    2.26017300
 H                  -0.70422900    1.45263500    1.71471300
```

C	2.33977400	-0.60903100	-2.51982200
H	2.67620300	-1.12077800	-3.41144000
C	3.11847100	0.28786600	-1.73989400
H	4.13581300	0.56910200	-1.95833800
C	1.06546200	-0.78147500	-1.90373200
H	0.27590400	-1.43361000	-2.24612600
C	3.67743200	-3.96584600	-2.16905800
H	4.14555100	-4.94379400	-1.99073600
H	2.73138100	-4.14913700	-2.68800300
H	4.33009300	-3.41379700	-2.85312100
C	5.78378100	-2.00927500	-0.77920000
H	6.52362500	-2.76467300	-0.48080400
H	5.79124900	-1.96154600	-1.87300700
H	6.13773900	-1.04379600	-0.40271400
C	4.51894700	-1.02968600	2.01369900
H	5.02777800	-1.63511400	2.77642000
H	5.27212900	-0.37461200	1.56432900
H	3.79118600	-0.39812500	2.53303000
C	1.66139800	-2.37420100	2.32917200
H	1.96138600	-3.07396400	3.12191100
H	1.70345300	-1.36511100	2.75239100
H	0.61830500	-2.58362300	2.08196700
C	1.12616800	-4.20577100	-0.25634900
H	0.93756400	-4.34633600	-1.32542600
H	1.27753900	-5.20223500	0.18158900
H	0.21942100	-3.78525800	0.18413700
S	-2.51450200	2.26683100	-1.25083300
O	-2.07424500	1.73795600	-2.54816600
O	-3.76490400	2.99504200	-1.08518800
C	-1.21506000	3.57095500	-0.85547700
F	-1.22802900	3.90871700	0.43166600
F	-1.47078500	4.64994100	-1.59866000
F	0.01424300	3.12063300	-1.17467900
N	-2.43541100	1.05206700	-0.12189400
C	-1.46793500	-0.00434800	-0.35451700
H	-1.38639000	-0.13055200	-1.43342900
C	-1.87991700	-1.32906200	0.26267600
C	-2.29751600	-2.35519400	-0.59069000
C	-1.89167800	-1.54061000	1.64840700
C	-2.70446700	-3.58841200	-0.07565200
H	-2.33343100	-2.18199100	-1.66353100
C	-2.29491800	-2.77379700	2.16168800
H	-1.61838600	-0.73789900	2.32639200
C	-2.69593700	-3.80140000	1.30219600
H	-3.03721400	-4.37146400	-0.75076900
H	-2.30919300	-2.92678100	3.23739500
H	-3.01488700	-4.75745400	1.70864600
C	-3.14862300	1.21948700	1.28122600
O	-2.43365000	1.79808900	2.14113100
C	-4.38063700	0.62516100	1.42695000
C	-5.12609600	-0.20788000	0.46508900
C	-5.76043900	-1.38269300	0.93574100
C	-5.30378300	0.09292300	-0.90293900
C	-6.48131300	-2.22300200	0.09003300

```
 H                  -5.66734300   -1.64957800    1.98418100
 C                  -6.01843000   -0.75293200   -1.75134000
 H                  -4.93187000    1.02985800   -1.29329600
 C                  -6.60848600   -1.92270600   -1.26816100
 H                  -6.94439400   -3.12045600    0.49587300
 H                  -6.13546600   -0.47626600   -2.79721500
 H                  -7.17313700   -2.57463700   -1.93035100
 C                  -4.92521000    0.70779200    2.84398800
 H                  -6.02035500    0.66402600    2.84971800
 H                  -4.56167700   -0.10796000    3.49182800
 H                  -4.60786200    1.64220000    3.31445200

H C O N F S 0
6-31G*
****
Fe 0
LANL2DZ
****

Fe 0
LANL2DZ
```

TS$_{G\text{-}H}$

```
%nprocshared=16
%mem=800MB
# opt=(ts,calcfc,noeigen) freq=noraman b3lyp/gen pseudo=read

Title Card Required

0 1
 C                  -4.65581800   -2.88650800   -0.73528000
 C                  -3.47872900   -2.66865000   -1.53090300
 C                  -3.54274400   -1.33993600   -2.06519400
 C                  -5.45578100   -1.69264800   -0.78948200
 C                  -4.76639000   -0.73801700   -1.60600700
 Fe                 -3.57877700   -1.28967700    0.01548300
 C                  -6.12931400    3.33008400    2.77555300
 C                  -6.10589700    4.17440500    1.49287100
 C                  -4.72394200    3.86500100    0.90717200
 N                  -4.47641100    2.47529300    1.30818900
 C                  -5.40259000    2.03987500    2.36401500
 C                  -3.36581000    1.79522300    0.89042700
 C                  -2.38235900    2.41490700    0.11183700
 C                  -1.21085200    1.72643000   -0.27715000
 N                  -0.91990400    0.46890600    0.00589700
 C                  -1.87254900   -0.18432300    0.74327500
 C                  -3.11111000    0.41150800    1.22374000
 H                  -7.13846600    3.13513000    3.15024800
 H                  -5.56867300    3.83758700    3.56953000
 H                  -6.89044700    3.84127200    0.80274700
 H                  -6.25004200    5.24318200    1.67524200
 H                  -4.69308400    3.96118200   -0.18407500
```

H	-3.95433400	4.53544700	1.32125400
H	-6.11154800	1.29298700	1.97945200
H	-4.85857200	1.58939600	3.19945600
H	-2.47053000	3.45842400	-0.16322300
H	-0.45706300	2.27792800	-0.83607000
C	-3.01986800	-1.79272000	1.94694400
H	-3.28742500	-2.73109900	2.41564400
C	-3.82417700	-0.61541200	1.94837500
H	-4.78116200	-0.52640600	2.43842200
C	-1.85325100	-1.55371700	1.16590700
H	-1.06450800	-2.26169500	0.94914700
C	-5.01944200	-4.16242100	-0.03017200
H	-5.58469900	-4.83508000	-0.69085900
H	-4.13054700	-4.70723700	0.30479300
H	-5.64147400	-3.97451300	0.85122400
C	-6.80383300	-1.51082600	-0.15142100
H	-7.60651900	-1.87221200	-0.80972900
H	-6.88299700	-2.06327100	0.79074600
H	-7.01171100	-0.45749500	0.06387200
C	-5.24739000	0.63606400	-1.97605100
H	-5.73451400	0.63131400	-2.96123300
H	-5.97438800	1.02063600	-1.25356500
H	-4.41881300	1.35031600	-2.02157500
C	-2.54324600	-0.69462500	-2.98175000
H	-2.83203400	-0.82285200	-4.03458800
H	-2.45754400	0.37971300	-2.79130500
H	-1.54410500	-1.12234000	-2.85812100
C	-2.39297300	-3.67514000	-1.78494000
H	-2.23225400	-4.32538500	-0.91858200
H	-2.64156400	-4.32079600	-2.63903900
H	-1.44097600	-3.18489200	-2.01110600
S	3.23440100	2.79382800	-0.32281900
O	1.89627000	3.03525600	-0.85779700
O	3.75667600	3.47517700	0.85184300
C	4.42304300	3.15172600	-1.74790400
F	4.37077000	2.14788900	-2.62726400
F	5.65815800	3.31003200	-1.30576900
F	4.00751300	4.27565900	-2.33710300
N	3.36674600	1.10016500	-0.08542700
C	2.43971500	0.21449500	-0.57176300
H	1.38670600	0.45190600	-0.41625500
C	2.74536500	-0.94305500	-1.35617300
C	1.68168300	-1.84600600	-1.61301900
C	4.01285900	-1.19216600	-1.94893300
C	1.88531800	-2.96857800	-2.40569800
H	0.70734800	-1.63697200	-1.17978300
C	4.19663700	-2.31537900	-2.74062800
H	4.82652300	-0.49390400	-1.78942100
C	3.14213700	-3.21133500	-2.96942800
H	1.06416400	-3.65561600	-2.59132800
H	5.16645500	-2.49445700	-3.19640300
H	3.29861200	-4.08542500	-3.59537100
C	4.52255400	0.51484100	0.58735700
O	5.64912600	0.75183600	0.14007300

```
 C                  4.11685200   -0.36246700    1.65390700
 C                  5.05203100   -1.34131500    2.18695900
 C                  4.66919500   -2.16898100    3.27807500
 C                  6.33957000   -1.57551300    1.62822800
 C                  5.50564900   -3.16441100    3.76775200
 H                  3.69851300   -2.03851500    3.74116000
 C                  7.16804600   -2.57472600    2.12492800
 H                  6.67770900   -0.94485700    0.81861900
 C                  6.76317300   -3.37693900    3.19553100
 H                  5.17507800   -3.77846500    4.60154300
 H                  8.14630000   -2.72422900    1.67507800
 H                  7.41889900   -4.15254700    3.58218700
 C                  2.81466300   -0.16101700    2.40194500
 H                  2.19205500   -1.06430100    2.41456300
 H                  3.02449400    0.09263700    3.45065400
 H                  2.21480400    0.64859600    1.98880600

H C O N F S 0
6-31G*
****
Fe 0
LANL2DZ
****

Fe 0
LANL2DZ
```

TS$_{G'-H'}$

```
%nprocshared=16
%mem=800MB
# opt=(ts,calcfc,noeigen) freq=noraman b3lyp/gen pseudo=read

Title Card Required

0 1
 C                  3.15121900   -3.21322400   -0.05497200
 C                  2.19346400   -2.95912300    0.98578400
 C                  2.67778100   -1.86466900    1.77380300
 C                  4.23803800   -2.28505300    0.10292100
 C                  3.94379900   -1.44944600    1.22901500
 Fe                 2.45055400   -1.27476100   -0.21251100
 C                  5.28118600    3.84261600   -1.27666100
 C                  5.33754400    4.18860400    0.21828200
 C                  3.95008200    3.77105100    0.71878100
 N                  3.59428500    2.63009500   -0.13748700
 C                  4.48647600    2.52870700   -1.30468900
 C                  2.45428500    1.91301700    0.05545700
 C                  1.52790800    2.28092000    1.04765000
 C                  0.29611700    1.62082500    1.17008900
 N                 -0.07835100    0.58599400    0.44235400
 C                  0.82029100    0.11531300   -0.48217500
```

C	2.09863500	0.76071600	-0.74104300
H	6.26818400	3.73811200	-1.73625300
H	4.73565200	4.62023100	-1.82394600
H	6.11190000	3.59428800	0.71784800
H	5.54646500	5.24452100	0.41124500
H	3.95157300	3.47451400	1.77366500
H	3.21874200	4.58477900	0.60039100
H	5.15357600	1.66087600	-1.20823500
H	3.90724700	2.41390600	-2.22438100
H	1.70748000	3.13740500	1.68393600
H	-0.42860100	1.97959800	1.89678500
C	1.85262600	-1.03393000	-2.18722000
H	2.05200800	-1.76624300	-2.95853300
C	2.73800900	0.00965000	-1.79655100
H	3.70406700	0.19382600	-2.23862400
C	0.70190800	-1.01109700	-1.35066800
H	-0.13706200	-1.68982500	-1.41102200
C	3.06072300	-4.30555200	-1.08240100
H	3.49715600	-5.24081600	-0.70434400
H	2.02185800	-4.51724500	-1.35525800
H	3.59750900	-4.04435600	-2.00027800
C	5.47803900	-2.23281600	-0.74375000
H	6.24382100	-2.92654300	-0.36957200
H	5.26934800	-2.50771000	-1.78290300
H	5.92165800	-1.23169800	-0.74996100
C	4.82618500	-0.37656400	1.80119500
H	5.43789700	-0.76859500	2.62594200
H	5.51419800	0.02898500	1.05206700
H	4.23964200	0.45800500	2.19937000
C	2.02695900	-1.27929900	2.99413100
H	2.40989700	-1.75204400	3.90963800
H	2.21799900	-0.20449300	3.07492200
H	0.94202300	-1.41231900	2.98372900
C	0.93185800	-3.74028600	1.21903600
H	0.44082100	-4.01434700	0.28012100
H	1.13953600	-4.66923800	1.76848300
H	0.21116400	-3.16803700	1.81001400
S	-2.46943700	1.66928200	-1.67159900
O	-1.06246900	1.58517400	-2.05194800
O	-3.53698000	1.58510800	-2.66110400
C	-2.65063500	3.37718700	-0.91754700
F	-3.90051900	3.60930400	-0.53753600
F	-2.28573200	4.28075500	-1.83167100
F	-1.84206400	3.49498300	0.15087800
N	-2.85188900	0.59947800	-0.45634000
C	-2.08732200	-0.09789000	0.52147500
H	-1.69440200	-1.06006400	0.22634200
C	-2.37435100	0.11668600	1.93725700
C	-1.98418900	-0.87315700	2.86429500
C	-3.01482200	1.27867200	2.41444100
C	-2.22655100	-0.70985800	4.22257900
H	-1.50870900	-1.78022400	2.50053400
C	-3.25685000	1.43234300	3.77962600
H	-3.34505100	2.03622200	1.71626200

```
 C                 -2.86248500    0.44820900    4.68756500
 H                 -1.92679200   -1.48561100    4.92209700
 H                 -3.76092700    2.32761200    4.13299000
 H                 -3.05167000    0.57685000    5.74973700
 C                 -4.28321700    0.18126300   -0.20386800
 O                 -5.09275600    1.03328800    0.14083500
 C                 -4.37606100   -1.23708400   -0.11849600
 C                 -3.62235200   -2.19218200   -0.93630300
 C                 -3.39560400   -3.50829600   -0.46616000
 C                 -3.13406400   -1.87939100   -2.22702800
 C                 -2.68532300   -4.43977500   -1.22046300
 H                 -3.77271900   -3.79838600    0.50987200
 C                 -2.42127900   -2.81377000   -2.97689300
 H                 -3.35790100   -0.91094200   -2.65916600
 C                 -2.18300700   -4.09891900   -2.48020300
 H                 -2.52884000   -5.44053900   -0.82418200
 H                 -2.07222500   -2.54007800   -3.96968000
 H                 -1.64167200   -4.83001200   -3.07493000
 C                 -5.50165900   -1.76131500    0.74124200
 H                 -5.11562200   -2.34583500    1.59049900
 H                 -6.08186700   -0.92874600    1.14243300
 H                 -6.17676800   -2.42191600    0.18103500

H C O N F S 0
6-31G*
****
Fe 0
LANL2DZ
****

Fe 0
LANL2DZ
```

Product H + catalyst

```
%nprocshared=16
%mem=800MB
# opt freq=noraman b3lyp/gen pseudo=read

Title Card Required

0 1
 C                  4.90182200   -2.83606400   -0.68275500
 C                  3.95554300   -3.23812500    0.32183100
 C                  3.95589300   -2.24376000    1.35372200
 C                  5.49599200   -1.59560300   -0.26331100
 C                  4.90931200   -1.22854200    0.99113600
 Fe                 3.43786900   -1.39084300   -0.47183500
 C                  4.00071100    4.55263600   -0.93480000
 C                  4.07856500    4.74725100    0.58649800
 C                  3.03766500    3.75284800    1.11268300
 N                  3.07938600    2.65228900    0.14197800
 C                  3.78251800    3.03903100   -1.08972200
```

C	2.32724900	1.52326900	0.30988600
C	1.47445800	1.37496000	1.40996300
C	0.62575700	0.25177700	1.53190100
N	0.54968000	-0.76213600	0.68874400
C	1.41006600	-0.68127300	-0.37489600
C	2.32342700	0.42440100	-0.63125700
H	4.89363000	4.89819500	-1.46401800
H	3.13935100	5.09710700	-1.33960700
H	5.07576500	4.47771700	0.95484200
H	3.86924900	5.77306700	0.90291800
H	3.27263700	3.38957500	2.11956800
H	2.03137200	4.19838300	1.14518300
H	4.74086300	2.50706200	-1.17576700
H	3.18398900	2.79955200	-1.97313500
H	1.38614900	2.15700900	2.15285200
H	-0.06152000	0.22488400	2.37610200
C	2.55438800	-1.14065500	-2.33176300
H	2.90609700	-1.64086600	-3.22503300
C	3.04733000	0.10437000	-1.84042700
H	3.81067400	0.69511200	-2.32276500
C	1.59124900	-1.65001100	-1.41503400
H	1.06454600	-2.59236300	-1.48408300
C	5.26216600	-3.61331200	-1.91732800
H	6.09053000	-4.30860400	-1.72032500
H	4.41842300	-4.20880600	-2.28120500
H	5.57782300	-2.95490600	-2.73351900
C	6.58565900	-0.85525400	-0.98564800
H	7.57822300	-1.22370700	-0.69004600
H	6.50547300	-0.97388600	-2.07126000
H	6.56071800	0.21754300	-0.76762800
C	5.26052800	-0.02813600	1.82340200
H	6.03675200	-0.27311000	2.56206100
H	5.64125800	0.79419300	1.20912100
H	4.39076700	0.34668000	2.37273300
C	3.13906900	-2.26755300	2.61332800
H	3.70450900	-2.71501000	3.44295900
H	2.84408900	-1.25896400	2.91922800
H	2.21990200	-2.84622900	2.48570900
C	3.13541600	-4.49680100	0.30872900
H	2.89474200	-4.81165300	-0.71190900
H	3.67393600	-5.32578800	0.78922100
H	2.19012400	-4.36427300	0.84390400
C	-3.67646600	-0.40056900	-1.28588400
S	-2.81020700	2.64152100	0.77674800
O	-3.84195800	3.66552100	0.78488300
O	-2.11093100	2.19632000	1.97490700
N	-3.37808100	1.26758900	-0.01112900
C	-2.95924500	-0.16832100	0.12525600
C	-4.86701400	-1.33947800	-1.35342200
C	-4.70978400	-2.69113400	-1.01043300
C	-6.11597800	-0.90179800	-1.81380300
C	-5.77759400	-3.58062800	-1.11539400
H	-3.75107800	-3.04771000	-0.64457500
C	-7.18524100	-1.79502600	-1.91965300

```
 H                  -6.25195400    0.13546500   -2.10281500
 C                  -7.02103000   -3.13505700   -1.57059300
 H                  -5.63863900   -4.62209400   -0.83782000
 H                  -8.14693700   -1.43782400   -2.27830600
 H                  -7.85366300   -3.82857300   -1.65237200
 H                  -1.87609000   -0.27494600    0.03392200
 C                  -3.43651000   -0.86696500    1.36890400
 C                  -2.57435900   -1.78039600    1.99135000
 C                  -4.71091900   -0.64822100    1.90945400
 C                  -2.98864900   -2.47492300    3.13007200
 H                  -1.57359500   -1.92600800    1.58906200
 C                  -5.11816600   -1.33698900    3.05060600
 H                  -5.38327900    0.06528000    1.44205800
 C                  -4.26008300   -2.25554900    3.66158100
 H                  -2.31110600   -3.17786600    3.60776600
 H                  -6.10682700   -1.15629200    3.46379300
 H                  -4.58007700   -2.79058000    4.55188200
 C                  -3.99377700    1.10768200   -1.27330500
 O                  -4.48143800    1.90404200   -2.02958300
 C                  -2.65791700   -0.70438600   -2.40306900
 H                  -2.28982800   -1.73092200   -2.30964500
 H                  -3.13640300   -0.59502400   -3.38113600
 H                  -1.79979800   -0.02499300   -2.35511400
 C                  -1.48742500    3.23402000   -0.42331100
 F                  -0.77532300    2.18855200   -0.86340200
 F                  -0.67808200    4.08231600    0.21572300
 F                  -2.05016500    3.84626600   -1.45996900

H C O N F S 0
6-31G*
****
Fe 0
LANL2DZ
****

Fe 0
LANL2DZ
```

Product H′ + catalyst

```
%nprocshared=16
%mem=800MB
# opt freq=noraman b3lyp/gen pseudo=read

Title Card Required

0 1
 C                   4.23384300   -2.77213600   -0.15311800
 C                   3.22804000   -2.87073500    0.86869300
 C                   3.37896400   -1.75033100    1.74904600
 C                   5.01559200   -1.59329800    0.10537500
 C                   4.48643400   -0.96110000    1.27756800
 Fe                  3.01925500   -1.09727100   -0.19056700
 C                   4.47657600    4.60272800   -1.17958000
```

C	4.54488400	4.90681800	0.32398400
C	3.33201300	4.15187700	0.88013900
N	3.20945200	2.98534500	-0.00115300
C	4.01244200	3.13860500	-1.22276800
C	2.28147300	2.00647900	0.22622500
C	1.35669500	2.10659700	1.27119000
C	0.36156200	1.12132500	1.46677100
N	0.20011400	0.02560300	0.74893000
C	1.10394600	-0.12357600	-0.27094600
C	2.16542700	0.81987900	-0.59266900
H	5.42869200	4.75067400	-1.69771800
H	3.72932400	5.24576600	-1.65958900
H	5.46947300	4.50210500	0.75310900
H	4.50844900	5.97599600	0.55189200
H	3.46986700	3.83547500	1.92059900
H	2.42187100	4.77071400	0.83960300
H	4.87211000	2.45279700	-1.21654600
H	3.41467000	2.92190000	-2.11270500
H	1.34305700	2.97472800	1.91837100
H	-0.36045900	1.27878900	2.26703100
C	2.24484600	-0.93633800	-2.10911500
H	2.55608600	-1.57070900	-2.92915800
C	2.88478600	0.27854500	-1.72282600
H	3.73892600	0.70865200	-2.22231600
C	1.18894400	-1.21427900	-1.19578300
H	0.53459400	-2.07523600	-1.20006300
C	4.47591900	-3.76496400	-1.25472400
H	5.17944900	-4.54728700	-0.93607800
H	3.55133300	-4.26495200	-1.56150900
H	4.90187600	-3.28748100	-2.14336400
C	6.21034600	-1.14101700	-0.68536200
H	7.13596100	-1.59329900	-0.30187200
H	6.12515300	-1.42048700	-1.74066300
H	6.33766400	-0.05421100	-0.64171600
C	5.02495300	0.27410900	1.94190800
H	5.75067700	0.01706500	2.72646500
H	5.53426800	0.92906500	1.22763700
H	4.22668700	0.85783100	2.41132500
C	2.54771300	-1.46867800	2.96716300
H	2.94950800	-1.98409700	3.85104300
H	2.51636800	-0.39868600	3.19400700
H	1.51338000	-1.79657400	2.82733100
C	2.21724500	-3.97257100	1.01314700
H	1.96240600	-4.41525800	0.04486900
H	2.59915000	-4.78033500	1.65360200
H	1.28899400	-3.60640000	1.46290400
C	-4.11267300	-1.32521600	-0.31450600
S	-3.50592100	2.41738100	-0.46406700
O	-4.68525700	3.12646200	-0.93490300
O	-2.75563600	2.80806600	0.72179100
N	-3.87260800	0.78008300	-0.31003700
C	-3.25855600	-0.28440200	0.54705400
H	-2.18750700	-0.37997100	0.34738800
C	-3.49704100	-0.16819000	2.02988500

```
 C                  -2.56960300   -0.76270000    2.89744800
 C                  -4.61312300    0.48251500    2.57209300
 C                  -2.76919400   -0.73101200    4.27876300
 H                  -1.67486900   -1.22054400    2.48288000
 C                  -4.80744000    0.52085900    3.95286400
 H                  -5.32373300    0.97588700    1.91509200
 C                  -3.89080100   -0.09214100    4.81020800
 H                  -2.04050100   -1.19401500    4.93895900
 H                  -5.67412100    1.03536000    4.35903500
 H                  -4.04375400   -0.06036900    5.88560600
 C                  -4.56100200   -0.11567800   -1.15989600
 O                  -5.21686400    0.06256800   -2.15043500
 C                  -2.28955900    2.39702200   -1.89012200
 F                  -1.27709500    1.57887500   -1.59926900
 F                  -1.82749300    3.63404900   -2.07412000
 F                  -2.89800800    1.97438700   -2.99660700
 C                  -3.28712000   -2.36196500   -1.06307400
 C                  -3.46192400   -2.58839600   -2.43477700
 C                  -2.37569100   -3.16050700   -0.35622200
 C                  -2.73231000   -3.58624700   -3.08565000
 H                  -4.16909500   -1.98364600   -2.99318500
 C                  -1.65140200   -4.15991500   -1.00647400
 H                  -2.23263900   -3.00165800    0.70939900
 C                  -1.82603700   -4.37475900   -2.37622000
 H                  -2.87673900   -3.74565600   -4.15088800
 H                  -0.95241900   -4.77133000   -0.44164300
 H                  -1.26277500   -5.15345500   -2.88355600
 C                  -5.29717600   -1.99027300    0.40492000
 H                  -4.93524500   -2.72416900    1.13196500
 H                  -5.91912000   -1.26283400    0.93360600
 H                  -5.91815100   -2.51057400   -0.33088400

H C O N F S 0
6-31G*
****
Fe 0
LANL2DZ
****

Fe 0
LANL2DZ
```

Optimized molecular structure of reactants, intermediates, and products for the imine-first mechanism of nucleophile-catalyzed Staudinger reaction involving N-Tf imine.

$TS_{E\text{-}J}$

```
%nprocshared=16
%mem=800MB
# opt=(modredundant,ts,calcfc,noeigen) freq=noraman b3lyp/gen pseudo=read
```

Title Card Required

0 1

C	2.08444800	-2.49728800	1.71042900
C	1.42519400	-1.43971800	2.42607600
C	2.36838000	-0.37661000	2.61385000
C	3.44261700	-2.09024400	1.46883200
C	3.61624900	-0.78071300	2.02302900
Fe	2.10954500	-0.76268000	0.58493200
C	6.53337500	0.51398500	-3.13544400
C	7.19290500	1.45344700	-2.11540800
C	5.99920900	2.21841500	-1.53189200
N	4.89743600	1.24731600	-1.58685800
C	5.23114500	0.09375800	-2.43816200
C	3.67793000	1.50646300	-1.03945500
C	3.39257500	2.76031400	-0.47070700
C	2.11120000	3.06334100	0.01761800
N	1.09143800	2.22129800	0.06330600
C	1.32923100	0.96435900	-0.43411300
C	2.59408800	0.55094000	-1.02389600
H	7.15545500	-0.34566200	-3.40066400
H	6.30395800	1.06007500	-4.05807800
H	7.68785700	0.87277400	-1.32782500
H	7.93663500	2.12137900	-2.55889200
H	6.16978900	2.54743100	-0.50085200
H	5.76108900	3.10962300	-2.13252100
H	5.38083900	-0.80789600	-1.82847500
H	4.42645600	-0.10723400	-3.15026500
H	4.13515900	3.54754800	-0.46694500
H	1.90735300	4.07228600	0.36970800
C	1.08310600	-1.20668500	-1.17346800
H	0.64594000	-2.17256600	-1.38521000
C	2.42386000	-0.81850300	-1.45447400
H	3.15730300	-1.45040600	-1.92981600
C	0.42562900	-0.13887800	-0.49988700
H	-0.58768700	-0.13756400	-0.12670200
C	1.49204200	-3.83011400	1.35057400
H	1.69381800	-4.57195800	2.13645300
H	0.40824700	-3.77715800	1.22231300
H	1.91113400	-4.22186300	0.41816500
C	4.49904800	-2.92256100	0.79919900
H	5.03026000	-3.55154600	1.52740300
H	4.06726900	-3.58995400	0.04648100
H	5.24938300	-2.30054900	0.29966000
C	4.88589800	0.02174200	2.04153100
H	5.43875000	-0.13556200	2.97836000
H	5.55278900	-0.25482000	1.21845000
H	4.68259800	1.09446400	1.95645600
C	2.12828900	0.91189100	3.34662000
H	2.50278400	0.85606900	4.37854600
H	2.63170500	1.75400300	2.85981400
H	1.06288700	1.15365600	3.39770100
C	0.01049400	-1.46717700	2.93221500
H	-0.63094100	-2.09690300	2.30924900

```
 H                 -0.03145000   -1.86897400    3.95438700
 H                 -0.43120300   -0.46591700    2.95659800
 C                 -0.82564700    3.00550600   -0.29690200
 O                 -1.10979600    2.48750300   -1.33543900
 C                 -1.11908300    3.87731600    0.69520600
 C                 -2.24426600    4.80565500    0.43542400
 C                 -2.71126200    5.65186500    1.46523200
 C                 -2.88169600    4.92322700   -0.82261100
 C                 -3.75788000    6.55010500    1.25473900
 H                 -2.25206100    5.61125300    2.44672000
 C                 -3.92584500    5.82232600   -1.02640100
 H                 -2.54303000    4.31320300   -1.65331500
 C                 -4.37892000    6.64408900    0.00983200
 H                 -4.08650100    7.18167300    2.07706400
 H                 -4.38322900    5.88678200   -2.01130600
 H                 -5.19284100    7.34509700   -0.15373700
 C                 -0.43074600    3.91143500    2.03593500
 H                  0.01783200    4.89260200    2.24899600
 H                 -1.14280300    3.70824900    2.84813300
 H                  0.34914700    3.15382600    2.09014000
 S                 -2.70388300   -4.01556800   -1.29828900
 O                 -3.22542300   -5.01191800   -2.22740600
 O                 -1.39072800   -3.39552100   -1.51923500
 C                 -2.60172100   -4.83064800    0.38122000
 F                 -2.30893200   -3.91279000    1.31398400
 F                 -1.62429500   -5.73829700    0.34314500
 F                 -3.75073100   -5.42261700    0.69152300
 N                 -3.92168900   -2.90368600   -0.94654200
 C                 -3.61870400   -1.65536100   -1.11265500
 H                 -2.61781400   -1.35705100   -1.44373700
 C                 -4.56952800   -0.58698500   -0.85670200
 C                 -4.13085100    0.74655100   -0.95962200
 C                 -5.90325600   -0.86198700   -0.49203800
 C                 -5.01207100    1.79205300   -0.68872700
 H                 -3.10345700    0.96964500   -1.24000700
 C                 -6.77725700    0.18464400   -0.23246100
 H                 -6.22758700   -1.89516300   -0.42182900
 C                 -6.33129400    1.51046800   -0.32780800
 H                 -4.66644100    2.81933500   -0.75281100
 H                 -7.80627500   -0.02432200    0.04592000
 H                 -7.01755200    2.32667200   -0.11825300

H C O N F S 0
6-31G*
****
Fe 0
LANL2DZ
****

Fe 0
LANL2DZ
```

Intermediate J

```
%nprocshared=16
%mem=800MB
# opt=(modredundant) freq=noraman b3lyp/gen pseudo=read

Title Card Required

0 1
 C                 -2.29511400    2.70816900    1.38767100
 C                 -1.99614700    1.57111300    2.21459400
 C                 -3.14248400    0.71256600    2.21047100
 C                 -3.63435600    2.55386500    0.88434500
 C                 -4.15395300    1.31502500    1.38453400
 Fe                -2.43648600    0.98269700    0.26067100
 C                 -6.49545600   -1.48775800   -3.19316000
 C                 -6.89776600   -2.50248400   -2.11357100
 C                 -5.54696500   -2.97767800   -1.56633300
 N                 -4.67387600   -1.80009300   -1.70799200
 C                 -5.28087800   -0.77706800   -2.57783400
 C                 -3.43767900   -1.74477800   -1.15504900
 C                 -2.88533400   -2.88469300   -0.52658700
 C                 -1.59915100   -2.87970800    0.00054100
 N                 -0.80902400   -1.80435900    0.03787300
 C                 -1.28074700   -0.64650500   -0.56209700
 C                 -2.58352400   -0.58244400   -1.20316400
 H                 -7.29483500   -0.78701600   -3.44946000
 H                 -6.19854000   -2.00921600   -4.11057100
 H                 -7.47092800   -2.00862700   -1.32013200
 H                 -7.50041200   -3.32916700   -2.49958600
 H                 -5.60062700   -3.28970800   -0.51809800
 H                 -5.15153600   -3.82204600   -2.14996600
 H                 -5.58540300    0.09513100   -1.98525300
 H                 -4.56777500   -0.44630400   -3.33693600
 H                 -3.42613700   -3.82098300   -0.50765700
 H                 -1.16501200   -3.78727500    0.40150400
 C                 -1.46274500    1.42299500   -1.52390700
 H                 -1.22095900    2.43060700   -1.83288500
 C                 -2.68693500    0.74319700   -1.77476700
 H                 -3.51751100    1.15224100   -2.32672200
 C                 -0.61699200    0.59650200   -0.73465000
 H                  0.36355800    0.84597200   -0.37066100
 C                 -1.38100100    3.87307000    1.13388500
 H                 -1.41794100    4.58741000    1.96804800
 H                 -0.34086800    3.55881100    1.01268700
 H                 -1.66317200    4.41664300    0.22719100
 C                 -4.35854700    3.53574300    0.00795500
 H                 -4.84184700    4.31933500    0.60749200
 H                 -3.67604200    4.03215500   -0.68914300
 H                 -5.14021800    3.05002400   -0.58502500
 C                 -5.53141900    0.75879400    1.16220300
 H                 -6.19721900    0.99405000    2.00451100
 H                 -5.99443700    1.17088000    0.25949900
 H                 -5.50948100   -0.33155700    1.05832300
```

C	-3.31408900	-0.55388400	2.99974800
H	-3.96174200	-0.38151900	3.87082700
H	-3.77352300	-1.35256100	2.40617500
H	-2.35754700	-0.92549200	3.37332100
C	-0.71746700	1.34914400	2.97360800
H	0.12084100	1.87971600	2.51387900
H	-0.80928500	1.71351800	4.00643000
H	-0.44357100	0.29075500	3.01839300
C	0.69045200	-1.89645000	0.44821100
O	1.43656700	-1.42131200	-0.43673900
C	0.93651200	-2.48933900	1.67785400
C	2.32289300	-2.75992600	2.10465900
C	2.57359500	-3.59346500	3.22214700
C	3.46337700	-2.22088800	1.45952300
C	3.86735800	-3.87562100	3.65849200
H	1.74510400	-4.04488900	3.75766200
C	4.75303100	-2.50272800	1.90203900
H	3.31463400	-1.56785300	0.61198700
C	4.97340600	-3.33406300	3.00340600
H	4.00729300	-4.52711600	4.51877700
H	5.59835800	-2.06116000	1.37796500
H	5.98267500	-3.55192600	3.34348400
C	-0.15177700	-2.87274500	2.66099900
H	-0.30451300	-3.96116100	2.75296300
H	0.10282300	-2.51898700	3.66953100
H	-1.11788800	-2.43487600	2.40868400
S	2.99727700	3.05942000	-0.89222300
O	3.54157700	4.39284500	-1.11286700
O	1.59849600	2.75196900	-1.22732000
C	3.12986700	2.69048200	0.94148500
F	2.76886200	1.42303400	1.16855200
F	2.28995300	3.50472300	1.59585800
F	4.36854000	2.88961900	1.38158400
N	4.09562900	1.94226400	-1.51541300
C	3.62648200	0.76004500	-1.77755900
H	2.58768200	0.48112900	-1.57742400
C	4.47162200	-0.27937000	-2.34660200
C	3.95239900	-1.58443900	-2.44391500
C	5.77731800	-0.00213000	-2.79854400
C	4.73874600	-2.59931800	-2.98771500
H	2.95846500	-1.79175200	-2.05401300
C	6.54760400	-1.01873900	-3.34723300
H	6.15957400	1.01012400	-2.71508900
C	6.02848000	-2.31760000	-3.44220900
H	4.34667900	-3.61014900	-3.05136600
H	7.55249500	-0.80795500	-3.70212000
H	6.63687600	-3.11046500	-3.86977000

H C O N F S 0
6-31G*
****
Fe 0
LANL2DZ
****

```
Fe 0
LANL2DZ
```

TS$_{J-K}$

```
%nprocshared=16
%mem=1GB
# freq=noraman b3lyp/gen pseudo=read

Title Card Required

0 1
 C                  -3.85433500   -2.88160300   -1.03830500
 C                  -2.70710400   -2.39716800   -1.75519600
 C                  -2.93917900   -1.02136300   -2.07813400
 C                  -4.80108100   -1.80495300   -0.93119400
 C                  -4.23185400   -0.65334500   -1.56661200
 Fe                 -3.01761000   -1.29792800   -0.00251400
 C                  -4.78147000    3.92878800    2.16124900
 C                  -4.25043200    4.69006300    0.93854000
 C                  -2.90164700    4.01368000    0.66930800
 N                  -3.11089600    2.61768500    1.10610500
 C                  -4.35376900    2.47966700    1.88911200
 C                  -2.18075500    1.65879400    0.91396200
 C                  -0.91943500    1.98636200    0.36344000
 C                   0.09932900    1.05806500    0.25282900
 N                  -0.03224800   -0.24080100    0.57569400
 C                  -1.26454000   -0.66120800    1.07620800
 C                  -2.35856500    0.27299300    1.28437200
 H                  -5.86312400    4.01931100    2.29321600
 H                  -4.29875200    4.29507400    3.07456700
 H                  -4.91940800    4.55067200    0.08132100
 H                  -4.14314700    5.76419200    1.11068000
 H                  -2.61240200    4.04397700   -0.38559200
 H                  -2.09447100    4.47471000    1.25493500
 H                  -5.11300100    1.94311200    1.30561200
 H                  -4.16463900    1.92153200    2.80883700
 H                  -0.67300400    3.00135900    0.08433300
 H                   1.09457400    1.38794700   -0.04258800
 C                  -3.00473300   -1.83225000    2.00663300
 H                  -3.59517000   -2.64452700    2.40903800
 C                  -3.44933200   -0.49218500    1.84570400
 H                  -4.42715000   -0.12995700    2.11767600
 C                  -1.68100800   -1.95450300    1.49690600
 H                  -1.07652600   -2.84644100    1.48073600
 C                  -4.05505800   -4.28532900   -0.54343300
 H                  -4.49622700   -4.91829800   -1.32564000
 H                  -3.10933100   -4.74833600   -0.24462200
 H                  -4.72781700   -4.31805100    0.31946300
 C                  -6.16240100   -1.89105000   -0.30244600
 H                  -6.91068000   -2.23191800   -1.03104200
 H                  -6.17941100   -2.59638600    0.53449800
```

H	-6.49663300	-0.91996000	0.07665300
C	-4.89118500	0.68441600	-1.74693300
H	-5.37386800	0.75657600	-2.73144600
H	-5.66686900	0.85881600	-0.99395200
H	-4.16839700	1.50422500	-1.67979900
C	-2.03649100	-0.12896100	-2.88011500
H	-2.36200300	-0.09168500	-3.92886000
H	-2.03347500	0.89863800	-2.50269500
H	-1.00400800	-0.48489500	-2.87223700
C	-1.50572900	-3.21200300	-2.14173600
H	-1.31014700	-4.01141000	-1.42070500
H	-1.65544700	-3.68516700	-3.12193200
H	-0.60110200	-2.60135900	-2.20902400
C	1.16642000	-1.17710500	0.68911400
O	1.14597700	-1.85907500	1.69730000
C	2.20179300	-1.10114800	-0.33627600
C	3.14206200	-2.28919000	-0.42566400
C	4.15869700	-2.24810800	-1.40655000
C	3.07120200	-3.44812700	0.37084100
C	5.04980900	-3.30490200	-1.58277000
H	4.26910000	-1.37085000	-2.03593500
C	3.96077600	-4.50925900	0.18497900
H	2.32435700	-3.51739000	1.14810400
C	4.95606700	-4.44929800	-0.78907700
H	5.82132800	-3.22729600	-2.34465700
H	3.87043800	-5.38920500	0.81777900
H	5.64946200	-5.27520500	-0.92476900
C	1.78195500	-0.63506900	-1.73541900
H	2.57776100	-0.07746600	-2.23059800
H	1.55440700	-1.50822800	-2.36053100
H	0.90451300	0.00738100	-1.73670900
S	3.78739700	2.37264300	-1.24251400
O	4.63255500	3.49036700	-0.80366700
O	4.32232700	1.47089100	-2.28636300
C	2.32217600	3.23773600	-2.01937900
F	1.47150600	2.36560900	-2.59532600
F	2.74560600	4.08832400	-2.95686800
F	1.62371200	3.93135100	-1.09709400
N	3.03548300	1.64008100	-0.01824900
C	3.45628900	0.39106900	0.37124400
H	4.28986800	-0.04627600	-0.17774100
C	3.53595500	0.16687700	1.84554700
C	4.32043000	-0.87652100	2.36210900
C	2.87241900	1.01737700	2.74464800
C	4.42452600	-1.07348400	3.73889800
H	4.86055800	-1.52905200	1.68397600
C	2.97235900	0.81527300	4.11976000
H	2.31381700	1.86024600	2.35112200
C	3.74645500	-0.23306100	4.62328600
H	5.04325700	-1.88182800	4.11986800
H	2.45845000	1.48836200	4.80180100
H	3.83148900	-0.38469700	5.69633800

H C O N F S 0

```
6-31G*
****
Fe 0
LANL2DZ
****

Fe 0
LANL2DZ
```

TS$_{J\text{-}K}$′

```
%nprocshared=16
%mem=1GB
# freq=noraman b3lyp/gen pseudo=read

Title Card Required

0 1
 C                  4.40300000   -2.79413800   -0.07027900
 C                  3.21834300   -2.85942800    0.74038000
 C                  3.25377000   -1.76317100    1.66215900
 C                  5.17781000   -1.66351600    0.36239700
 C                  4.46469400   -1.02280900    1.42743700
 Fe                 3.31069700   -1.05203200   -0.30522300
 C                  4.63191800    4.74286200   -0.19290300
 C                  4.15249200    4.90091700    1.25708500
 C                  2.87826700    4.04999300    1.29336800
 N                  3.14414200    2.97000900    0.32219800
 C                  4.32550200    3.27214000   -0.50769800
 C                  2.28428200    1.94651500    0.13366600
 C                  1.04973700    1.91077200    0.81969900
 C                  0.06838400    0.98251100    0.51973100
 N                  0.24363900   -0.03468000   -0.34297900
 C                  1.48028300   -0.13392100   -0.97966900
 C                  2.51693300    0.86322200   -0.79490200
 H                  5.69075200    4.98004100   -0.32876600
 H                  4.05215000    5.39440500   -0.85655200
 H                  4.89728200    4.49548300    1.95218000
 H                  3.95974500    5.93969000    1.53793500
 H                  2.67544900    3.63030400    2.28400400
 H                  1.99743400    4.62731900    0.98114600
 H                  5.16506200    2.62225300   -0.22633900
 H                  4.10062100    3.11086000   -1.56419200
 H                  0.76589000    2.71338000    1.48528800
 H                 -0.93700800    1.11029100    0.90844200
 C                  3.22906100   -0.69941600   -2.35090200
 H                  3.83950500   -1.23117300   -3.06843200
 C                  3.61523800    0.47601600   -1.65074800
 H                  4.56229400    0.97770400   -1.76305900
 C                  1.93771700   -1.10585100   -1.91068400
 H                  1.37564700   -1.95616400   -2.26260500
 C                  4.78896800   -3.77828700   -1.13749300
```

H	5.31810600	-4.63907500	-0.70583100
H	3.91198500	-4.16595500	-1.66554800
H	5.45293900	-3.32984800	-1.88322800
C	6.52040500	-1.25813200	-0.17606400
H	7.33047300	-1.76552700	0.36574900
H	6.62812000	-1.51506300	-1.23484600
H	6.68939400	-0.18070500	-0.07764200
C	4.93902000	0.15726700	2.22738700
H	5.52279400	-0.17004900	3.09906100
H	5.58332300	0.81726200	1.63717600
H	4.10347500	0.75555100	2.60441200
C	2.25197600	-1.47137200	2.74223300
H	2.57172500	-1.90866000	3.69834100
H	2.12554400	-0.39550200	2.90008500
H	1.26895000	-1.88499900	2.50560100
C	2.16752500	-3.92993900	0.66466200
H	2.04551400	-4.30772000	-0.35516100
H	2.43522500	-4.78493400	1.30086800
H	1.19153800	-3.56747600	0.99878500
C	-0.92853300	-0.80874800	-0.94043800
O	-0.89953700	-0.85972600	-2.15313600
C	-1.92051400	-1.38530700	-0.03570300
C	-2.68668000	-2.51052700	-0.68096800
C	-2.89561400	-3.72836200	-0.00523100
C	-3.26547100	-2.38135900	-1.96381100
C	-3.63748400	-4.76506500	-0.57569900
H	-2.47740200	-3.88361500	0.98266000
C	-4.00987700	-3.41551500	-2.52815400
H	-3.13031300	-1.46523200	-2.52456200
C	-4.20234500	-4.61666800	-1.84101200
H	-3.77254100	-5.69056400	-0.02089000
H	-4.44388800	-3.27675800	-3.51527600
H	-4.78376000	-5.42054100	-2.28495100
C	-1.47429700	-1.66085900	1.39611000
H	-2.32554200	-1.97365000	2.00394600
H	-0.72577900	-2.46475100	1.44482200
H	-1.04923200	-0.78630200	1.88888200
S	-2.76351600	2.30502400	-1.08642000
O	-1.48497200	3.03623600	-1.08809700
O	-3.23445200	1.66542700	-2.32726800
C	-4.03237300	3.62442200	-0.72446400
F	-5.25047400	3.08009400	-0.60535400
F	-4.05565000	4.51302700	-1.72722000
F	-3.73774200	4.27178600	0.41136600
N	-2.89242200	1.39175300	0.24617200
C	-3.41717000	0.13966200	0.10804300
H	-3.82079400	-0.13664900	-0.86513200
C	-4.20689100	-0.35490000	1.26870000
C	-5.14999400	-1.38286800	1.09960400
C	-4.06996900	0.23015300	2.53945500
C	-5.92110600	-1.82375200	2.17581600
H	-5.28797300	-1.82745200	0.11978200
C	-4.83640500	-0.21572000	3.61384800
H	-3.37597000	1.05514300	2.66038100

```
 C                  -5.76323500   -1.24751400    3.43759500
 H                  -6.65124500   -2.61434300    2.02392900
 H                  -4.72053000    0.25062600    4.58913300
 H                  -6.36605800   -1.59020700    4.27474300

H C O N F S 0
6-31G*
****
Fe 0
LANL2DZ
****

Fe 0
LANL2DZ
```

Intermediate K

```
%nprocshared=16
%mem=1GB
# opt freq=noraman b3lyp/gen pseudo=read

Title Card Required

0 1
 C                  -4.04744600   -2.78889200   -0.80537800
 C                  -2.88795400   -2.41743200   -1.56856200
 C                  -3.05782700   -1.06077600   -1.99636800
 C                  -4.94159600   -1.66396900   -0.77605700
 C                  -4.32686100   -0.59465600   -1.50436500
 Fe                 -3.12029900   -1.17152700    0.09454400
 C                  -4.97359700    4.07867600    2.13053100
 C                  -4.54810100    4.79840900    0.84309600
 C                  -3.19619000    4.15431400    0.51658900
 N                  -3.32484000    2.77406100    1.03415000
 C                  -4.51656400    2.63289700    1.89381800
 C                  -2.37553600    1.84044200    0.83460800
 C                  -1.15693100    2.18287300    0.19607600
 C                  -0.09476400    1.30742100    0.10755800
 N                  -0.15697500    0.02106300    0.52582200
 C                  -1.36060500   -0.42725800    1.08066000
 C                  -2.48271700    0.47079300    1.28603200
 H                  -6.04684400    4.14196500    2.32947600
 H                  -4.44484900    4.50004100    2.99307700
 H                  -5.26555000    4.59899000    0.03856500
 H                  -4.46653500    5.88200900    0.96048100
 H                  -2.97794500    4.13908200   -0.55542200
 H                  -2.36973900    4.66890200    1.02446000
 H                  -5.29002100    2.05049600    1.37634500
 H                  -4.25712900    2.11905300    2.82201600
 H                  -0.97060000    3.19523300   -0.13499300
 H                   0.90976300    1.63936800   -0.18672900
 C                  -3.02004400   -1.62351400    2.12039800
 H                  -3.56363800   -2.44062400    2.57525000
 C                  -3.52028400   -0.30855200    1.92311900
```

H	-4.50191800	0.02760900	2.21483400
C	-1.71049600	-1.71597200	1.56695700
H	-1.08052100	-2.59141600	1.56066600
C	-4.30899200	-4.14184400	-0.20733800
H	-4.79458100	-4.80478800	-0.93654100
H	-3.38210000	-4.63063800	0.10888300
H	-4.96739600	-4.08064000	0.66499100
C	-6.30096800	-1.63917900	-0.13712400
H	-7.06629600	-2.01114600	-0.83192500
H	-6.34238000	-2.26834600	0.75792800
H	-6.59464100	-0.62612000	0.15596000
C	-4.92848000	0.75215600	-1.78854600
H	-5.44206200	0.75441800	-2.75981000
H	-5.66733100	1.03602100	-1.03204200
H	-4.16611700	1.53708900	-1.82428500
C	-2.13110500	-0.27420000	-2.87821900
H	-2.44901800	-0.33872700	-3.92789200
H	-2.11059900	0.78627100	-2.60807900
H	-1.10490900	-0.64327200	-2.82465500
C	-1.73476500	-3.32256500	-1.89512100
H	-1.52006900	-4.01666400	-1.07666100
H	-1.95246800	-3.92507300	-2.78750500
H	-0.81867000	-2.76172100	-2.09743300
C	1.09331100	-0.77664300	0.78062600
O	1.14524000	-1.30025900	1.86293200
C	2.18030300	-0.88880500	-0.29598400
C	2.75101600	-2.33447300	-0.36353100
C	3.76678000	-2.58337100	-1.30719600
C	2.29472000	-3.42958700	0.38392500
C	4.30411300	-3.85595200	-1.48970800
H	4.15991300	-1.77146400	-1.91023500
C	2.82708100	-4.70919500	0.19727600
H	1.54084800	-3.29693100	1.14719800
C	3.83367400	-4.93355600	-0.73793400
H	5.09302600	-4.00070300	-2.22311900
H	2.45038700	-5.53073000	0.80154900
H	4.24924600	-5.92791600	-0.87694200
C	1.64197400	-0.56516500	-1.71011000
H	2.45137800	-0.63231500	-2.43712100
H	0.87803400	-1.29541000	-1.99752300
H	1.23445500	0.43822400	-1.79969000
S	3.63489100	2.67840500	-0.57702700
O	3.09388500	3.96571500	-0.11318300
O	5.09158600	2.45067800	-0.60773200
C	3.19961000	2.72524500	-2.39884700
F	3.62270000	1.60251200	-3.02524100
F	3.74038100	3.77459500	-3.02895500
F	1.85357700	2.79110800	-2.56964200
N	2.77243100	1.48953900	0.00639100
C	3.35066400	0.16538100	0.09575400
H	4.12658200	-0.00552200	-0.66148900
C	3.98264400	-0.11865200	1.46260900
C	5.01164200	-1.06090300	1.59883200
C	3.57688700	0.59083700	2.60043200

```
 C                   5.60217700   -1.30461100    2.83948800
 H                   5.36682800   -1.60097500    0.72649300
 C                   4.16234200    0.34674400    3.84284900
 H                   2.82252300    1.36291000    2.49095000
 C                   5.17486200   -0.60589100    3.96960900
 H                   6.40541100   -2.03321600    2.91871300
 H                   3.83508800    0.91277300    4.71179700
 H                   5.63818200   -0.78998600    4.93587400

H C O N F S 0
6-31G*
****
Fe 0
LANL2DZ
****

Fe 0
LANL2DZ
```

Intermediate K′

```
%nprocshared=16
%mem=1GB
# opt freq=noraman b3lyp/gen pseudo=read

Title Card Required

0 1
 C                   4.34430400   -2.72015300    0.28604800
 C                   3.14301100   -2.66487400    1.07301600
 C                   3.19135400   -1.47419900    1.86811100
 C                   5.14235600   -1.56854200    0.60926700
 C                   4.42575300   -0.79491300    1.57901300
 Fe                  3.30352900   -0.99037800   -0.16738500
 C                   4.89719100    4.70765400   -0.70075700
 C                   4.42832700    5.03799200    0.72317000
 C                   3.11422300    4.25975900    0.84369700
 N                   3.32644300    3.07039100   -0.00838500
 C                   4.52038400    3.22809700   -0.86198900
 C                   2.42674400    2.07061600   -0.08272300
 C                   1.19207500    2.16281500    0.60560400
 C                   0.16394500    1.26457600    0.40061100
 N                   0.29054100    0.15523700   -0.36265900
 C                   1.52657800   -0.07661300   -0.97118600
 C                   2.60876300    0.88434100   -0.88920900
 H                   5.96593000    4.87771800   -0.85693300
 H                   4.34846100    5.31333100   -1.43079900
 H                   5.15347800    4.67076000    1.45898100
 H                   4.28721800    6.10835600    0.89410100
 H                   2.89125600    3.95663400    1.87138300
 H                   2.26224400    4.84148200    0.46738300
```

H	5.32700100	2.57163300	-0.50992600
H	4.28594900	2.96649700	-1.89619000
H	0.94907900	3.04247600	1.18459500
H	-0.85152100	1.46072100	0.74069600
C	3.25659000	-0.88213700	-2.24273300
H	3.84700500	-1.52399400	-2.88260700
C	3.69307500	0.34831300	-1.68065700
H	4.66498600	0.78766900	-1.83667000
C	1.94225600	-1.17142900	-1.77604600
H	1.34483300	-2.03341800	-2.03107300
C	4.72648700	-3.82538200	-0.65677600
H	5.24261500	-4.63583300	-0.12402400
H	3.84847600	-4.26196700	-1.14322900
H	5.40032300	-3.47221200	-1.44378400
C	6.50502000	-1.26057600	0.05698900
H	7.28910500	-1.74731900	0.65292800
H	6.61476700	-1.61298200	-0.97355100
H	6.71365700	-0.18575900	0.06261300
C	4.89500300	0.46747100	2.24320700
H	5.36911900	0.25118900	3.21049500
H	5.63206300	0.99858300	1.63271000
H	4.06253900	1.15224200	2.43419800
C	2.18954600	-1.04101900	2.89951700
H	2.51133900	-1.34861100	3.90414000
H	2.06149900	0.04611000	2.91616400
H	1.20672300	-1.48345900	2.72323900
C	2.06753700	-3.71267500	1.09924200
H	1.95074400	-4.19981500	0.12658600
H	2.30538900	-4.49633100	1.83183300
H	1.09522500	-3.29455800	1.37313700
C	-0.91827500	-0.54285600	-0.94386600
O	-0.90202400	-0.65632800	-2.13893100
C	-2.00622900	-1.16358300	-0.02862800
C	-2.29365600	-2.58319100	-0.57108100
C	-2.07652800	-3.72908000	0.20845100
C	-2.79416200	-2.76949700	-1.87384400
C	-2.35057600	-5.00857800	-0.28410900
H	-1.70125900	-3.64151200	1.22131500
C	-3.06768100	-4.04447100	-2.36667100
H	-2.96326200	-1.91340200	-2.51483200
C	-2.84815100	-5.17379200	-1.57447000
H	-2.17699400	-5.87337000	0.35137400
H	-3.45702900	-4.15250600	-3.37564600
H	-3.06570500	-6.16695500	-1.95851700
C	-1.58468700	-1.19271200	1.44802200
H	-2.33743100	-1.72240200	2.03523500
H	-0.62140800	-1.69230400	1.58739900
H	-1.51793900	-0.18336700	1.85292300
S	-3.01787800	2.33675800	-0.94337700
O	-1.93318300	3.33520500	-0.84486900
O	-3.50559400	1.93509800	-2.27846300
C	-4.47412700	3.25177400	-0.21523100
F	-5.56767500	2.47019700	-0.21147600
F	-4.75079600	4.36148100	-0.91655400

```
 F                  -4.21996600    3.61497700    1.05758200
 N                  -2.70693300    1.14894600    0.05639700
 C                  -3.25373200   -0.17267600   -0.20154000
 H                  -3.59021400   -0.26991900   -1.24043600
 C                  -4.41674900   -0.54691000    0.72081500
 C                  -5.28402800   -1.60142100    0.39759600
 C                  -4.65089000    0.16822200    1.90316100
 C                  -6.34176500   -1.94392500    1.24137700
 H                  -5.13969200   -2.15336100   -0.52610400
 C                  -5.70737900   -0.17349700    2.74900500
 H                  -4.01497800    1.01761700    2.12819900
 C                  -6.55439100   -1.23493200    2.42527100
 H                  -7.00594100   -2.75994000    0.96698400
 H                  -5.87532500    0.39984600    3.65765800
 H                  -7.38114600   -1.49756400    3.08069400

H C O N F S 0
6-31G*
****
Fe 0
LANL2DZ
****

Fe 0
LANL2DZ
```

$TS_{K\text{-}L}$

```
%nprocshared=16
%mem=1GB
# opt=(ts,calcfc,noeigen) freq=noraman b3lyp/gen pseudo=read

Title Card Required

0 1
 C                   3.82661900   -3.02464400    0.22880600
 C                   2.77801700   -2.77193100    1.17862100
 C                   3.07120700   -1.53484600    1.83825300
 C                   4.77543100   -1.94759800    0.31445700
 C                   4.30346000   -1.02407900    1.30183400
 Fe                  2.90419800   -1.22915900   -0.22821100
 C                   5.32121700    3.94622100   -1.74136700
 C                   5.20014900    4.47699800   -0.30584800
 C                   3.81314400    3.98825800    0.12654200
 N                   3.63899200    2.72160200   -0.60726900
 C                   4.65507200    2.56501200   -1.66357900
 C                   2.56710900    1.91940100   -0.41106500
 C                   1.52261500    2.31241000    0.45249300
 C                   0.33042300    1.60152800    0.52431600
 N                   0.10647700    0.43896800   -0.09293700
 C                   1.15428900   -0.08844500   -0.83643500
 C                   2.38178200    0.65079400   -1.07600000
 H                   6.35372000    3.88543500   -2.09665000
 H                   4.76442800    4.59040300   -2.43157600
 H                   5.97229100    4.03062500    0.33196300
```

H	5.29328400	5.56414100	-0.23673400
H	3.74130400	3.81772600	1.20587400
H	3.02705000	4.70240000	-0.15648700
H	5.38109000	1.78928400	-1.38474300
H	4.18577600	2.27436400	-2.60644900
H	1.55913600	3.26023800	0.97104600
H	-0.50523900	2.01454000	1.06582800
C	2.45502000	-1.34291200	-2.25279800
H	2.79716800	-2.15084100	-2.88595200
C	3.18822800	-0.15998200	-1.95990600
H	4.16705900	0.07264900	-2.34733200
C	1.23004300	-1.32940100	-1.52808000
H	0.46075800	-2.08497100	-1.54786300
C	3.94851700	-4.24137800	-0.64410200
H	4.47168700	-5.05177900	-0.11761300
H	2.96801600	-4.62635300	-0.94151400
H	4.51305500	-4.03025600	-1.55802000
C	6.06124100	-1.84215800	-0.45494300
H	6.87859800	-2.35182500	0.07383300
H	5.98165500	-2.29917200	-1.44658300
H	6.36612600	-0.79961900	-0.59402300
C	5.00380300	0.21756600	1.77580500
H	5.65450700	-0.00175600	2.63403900
H	5.63415000	0.65567900	0.99504400
H	4.29003900	0.98343300	2.09646900
C	2.30093300	-0.90253700	2.96254600
H	2.86708100	-0.96359300	3.90203800
H	2.09119400	0.15621400	2.77438300
H	1.34510300	-1.40466200	3.12766500
C	1.61968000	-3.68485500	1.46298300
H	1.32142600	-4.25083900	0.57576200
H	1.87899700	-4.41219400	2.24511400
H	0.73650400	-3.13754400	1.80149900
C	-1.40367400	-0.10223400	-0.48034000
O	-1.44618800	-0.38543500	-1.66220800
C	-2.17797500	-0.88866500	0.66293900
C	-2.41476700	-2.37104300	0.36167500
C	-2.30617200	-3.34379600	1.37213200
C	-2.76559200	-2.82205000	-0.92587600
C	-2.53255000	-4.69827700	1.11632500
H	-2.04541800	-3.05855600	2.38459000
C	-2.98686100	-4.17506900	-1.18349000
H	-2.85116200	-2.10947800	-1.73236600
C	-2.87309500	-5.12344300	-0.16625800
H	-2.44239500	-5.41696700	1.92717500
H	-3.25679200	-4.48472800	-2.18997400
H	-3.05126600	-6.17633100	-0.36964900
C	-1.53898800	-0.67129300	2.04322500
H	-2.18471200	-1.07040600	2.83117300
H	-0.56206600	-1.15451200	2.12049000
H	-1.41526500	0.39398600	2.24320900
S	-2.94293900	2.69738500	-0.32777000
O	-1.76316400	3.33354000	-0.92759200
O	-4.27016500	2.87053000	-0.92298500

```
 C                 -3.12597500    3.52643700    1.34441000
 F                 -4.09551700    2.92951600    2.05723100
 F                 -3.42540400    4.82031000    1.20753700
 F                 -1.97864400    3.43557800    2.05627200
 N                 -2.53379600    1.19125600    0.07483600
 C                 -3.40965100    0.11790300    0.58130000
 H                 -3.76292200    0.33575600    1.59659200
 C                 -4.61569900   -0.28678000   -0.24550400
 C                 -5.70087500   -0.87511700    0.41690200
 C                 -4.68772600   -0.11777900   -1.63419800
 C                 -6.82832500   -1.30060900   -0.28643800
 H                 -5.66443200   -0.99925400    1.49758400
 C                 -5.81570000   -0.53817000   -2.33865400
 H                 -3.85974200    0.34182300   -2.16068300
 C                 -6.88858700   -1.13238500   -1.67040400
 H                 -7.65980400   -1.75373500    0.24748400
 H                 -5.85834800   -0.39269400   -3.41502100
 H                 -7.76795800   -1.45348100   -2.22299200

H C O N F S 0
6-31G*
****
Fe 0
LANL2DZ
****

Fe 0
LANL2DZ
```

TS$_{K'-L'}$

```
%nprocshared=16
%mem=1GB
# opt=(ts,calcfc,noeigen) freq=noraman b3lyp/gen pseudo=read

Title Card Required

0 1
 C                  4.07001600   -2.78055000    0.53905900
 C                  2.84589600   -2.73378400    1.28966900
 C                  2.83657700   -1.51543500    2.04316300
 C                  4.82876900   -1.59936300    0.84784700
 C                  4.06182800   -0.81408600    1.76699900
 Fe                 3.00192900   -1.09401800   -0.00547000
 C                  5.18162800    4.31218200   -1.11658100
 C                  4.81164200    4.82680500    0.28172000
 C                  3.43290300    4.20193700    0.52510600
 N                  3.48594100    2.92585900   -0.21078900
 C                  4.64924300    2.87237400   -1.11449900
 C                  2.46363900    2.03817700   -0.18662300
 C                  1.26893700    2.32970100    0.50548100
 C                  0.13406400    1.53597100    0.36683500
 N                  0.10420000    0.38073200   -0.30090500
```

C	1.29407300	-0.05067500	-0.86452300
C	2.48776700	0.77483600	-0.88704700
H	6.25425200	4.35429400	-1.32556500
H	4.66500500	4.89908600	-1.88473000
H	5.52877700	4.46031900	1.02583500
H	4.78574700	5.91778600	0.34849300
H	3.22898600	4.02457500	1.58642800
H	2.62747900	4.83518800	0.12685500
H	5.40269100	2.17150300	-0.72949100
H	4.34608300	2.53958100	-2.10969000
H	1.15516100	3.26128600	1.04254400
H	-0.81920600	1.86639400	0.75689500
C	2.88636500	-1.17216500	-2.07943100
H	3.38083400	-1.93409600	-2.66712800
C	3.48040600	0.04505500	-1.64278200
H	4.49251700	0.34981500	-1.85523400
C	1.56247200	-1.25947100	-1.56462800
H	0.85669100	-2.05928800	-1.73159200
C	4.51270000	-3.91229400	-0.34425900
H	5.06933500	-4.66579000	0.23042800
H	3.65987400	-4.41993400	-0.80598100
H	5.16946700	-3.56745100	-1.14948700
C	6.20736800	-1.28255600	0.34159300
H	6.97464300	-1.71181100	1.00073200
H	6.37734700	-1.68982900	-0.66026100
H	6.38835200	-0.20338400	0.29614100
C	4.47778000	0.47977800	2.40616700
H	4.93504500	0.30379700	3.38983800
H	5.21151700	1.01788200	1.79793600
H	3.62048300	1.14365600	2.55806600
C	1.80169800	-1.06864500	3.03645700
H	2.14195500	-1.26210200	4.06336500
H	1.59705000	0.00453300	2.95768000
H	0.85314300	-1.59298100	2.90283700
C	1.81722700	-3.82755300	1.32791700
H	1.63946100	-4.25680400	0.33692000
H	2.14535800	-4.64403700	1.98618400
H	0.85694200	-3.47015800	1.70726800
C	-1.28225000	-0.19076600	-1.01944700
O	-1.13903300	-0.22911100	-2.22394200
C	-2.11630600	-1.24432600	-0.18960600
C	-2.30297900	-2.57495000	-0.91734600
C	-2.02919400	-3.80532800	-0.29894700
C	-2.78504400	-2.60563400	-2.24041700
C	-2.22867200	-5.01725600	-0.96628000
H	-1.66507200	-3.83590400	0.72175200
C	-2.98313200	-3.81332300	-2.90759300
H	-2.97983700	-1.67576000	-2.76046400
C	-2.70782400	-5.02771900	-2.27461100
H	-2.01346300	-5.95174600	-0.45386400
H	-3.35380300	-3.80259200	-3.92930400
H	-2.86714200	-5.96853400	-2.79518300
C	-1.58052700	-1.40535400	1.23308200
H	-2.25078700	-2.02356400	1.83705800

```
 H                  -0.59074800   -1.86810600    1.21963700
 H                  -1.49331700   -0.43689000    1.72833700
 S                  -3.06542200    2.18176200   -1.25329000
 O                  -1.87967000    2.90226200   -1.72863100
 O                  -4.17315200    1.84555600   -2.15580900
 C                  -3.81465300    3.37090800   -0.01273500
 F                  -4.96971300    2.90614600    0.47425900
 F                  -4.03733100    4.55042200   -0.60162000
 F                  -2.96536100    3.56579200    1.01803500
 N                  -2.55807700    0.93567100   -0.32119800
 C                  -3.37535700   -0.29441000   -0.21444100
 H                  -3.93109200   -0.47046600   -1.14058800
 C                  -4.33391600   -0.40285200    0.95162300
 C                  -5.34093800   -1.37860900    0.90364700
 C                  -4.23254300    0.40012200    2.09410700
 C                  -6.21741900   -1.55516000    1.97417400
 H                  -5.43707400   -2.00174200    0.01769800
 C                  -5.11314400    0.22934200    3.16398700
 H                  -3.47484000    1.17532000    2.13129900
 C                  -6.10552100   -0.75079400    3.11069200
 H                  -6.99283100   -2.31480700    1.91695200
 H                  -5.02576600    0.86853700    4.03909700
 H                  -6.79208500   -0.88103100    3.94321200

H C O N F S 0
6-31G*
****
Fe 0
LANL2DZ
****

Fe 0
LANL2DZ
```

Product L + catalyst

```
%nprocshared=16
%mem=800MB
# opt freq=noraman b3lyp/gen pseudo=read

Title Card Required

0 1
 C                   4.90182200   -2.83606400   -0.68275500
 C                   3.95554300   -3.23812500    0.32183100
 C                   3.95589300   -2.24376000    1.35372200
 C                   5.49599200   -1.59560300   -0.26331100
 C                   4.90931200   -1.22854200    0.99113600
 Fe                  3.43786900   -1.39084300   -0.47183500
 C                   4.00071100    4.55263600   -0.93480000
 C                   4.07856500    4.74725100    0.58649800
 C                   3.03766500    3.75284800    1.11268300
 N                   3.07938600    2.65228900    0.14197800
```

C	3.78251800	3.03903100	-1.08972200
C	2.32724900	1.52326900	0.30988600
C	1.47445800	1.37496000	1.40996300
C	0.62575700	0.25177700	1.53190100
N	0.54968000	-0.76213600	0.68874400
C	1.41006600	-0.68127300	-0.37489600
C	2.32342700	0.42440100	-0.63125700
H	4.89363000	4.89819500	-1.46401800
H	3.13935100	5.09710700	-1.33960700
H	5.07576500	4.47771700	0.95484200
H	3.86924900	5.77306700	0.90291800
H	3.27263700	3.38957500	2.11956800
H	2.03137200	4.19838300	1.14518300
H	4.74086300	2.50706200	-1.17576700
H	3.18398900	2.79955200	-1.97313500
H	1.38614900	2.15700900	2.15285200
H	-0.06152000	0.22488400	2.37610200
C	2.55438800	-1.14065500	-2.33176300
H	2.90609700	-1.64086600	-3.22503300
C	3.04733000	0.10437000	-1.84042700
H	3.81067400	0.69511200	-2.32276500
C	1.59124900	-1.65001100	-1.41503400
H	1.06454600	-2.59236300	-1.48408300
C	5.26216600	-3.61331200	-1.91732800
H	6.09053000	-4.30860400	-1.72032500
H	4.41842300	-4.20880600	-2.28120500
H	5.57782300	-2.95490600	-2.73351900
C	6.58565900	-0.85525400	-0.98564800
H	7.57822300	-1.22370700	-0.69004600
H	6.50547300	-0.97388600	-2.07126000
H	6.56071800	0.21754300	-0.76762800
C	5.26052800	-0.02813600	1.82340200
H	6.03675200	-0.27311000	2.56206100
H	5.64125800	0.79419300	1.20912100
H	4.39076700	0.34668000	2.37273300
C	3.13906900	-2.26755300	2.61332800
H	3.70450900	-2.71501000	3.44295900
H	2.84408900	-1.25896400	2.91922800
H	2.21990200	-2.84622900	2.48570900
C	3.13541600	-4.49680100	0.30872900
H	2.89474200	-4.81165300	-0.71190900
H	3.67393600	-5.32578800	0.78922100
H	2.19012400	-4.36427300	0.84390400
C	-3.67646600	-0.40056900	-1.28588400
S	-2.81020700	2.64152100	0.77674800
O	-3.84195800	3.66552100	0.78488300
O	-2.11093100	2.19632000	1.97490700
N	-3.37808100	1.26758900	-0.01112900
C	-2.95924500	-0.16832100	0.12525600
C	-4.86701400	-1.33947800	-1.35342200
C	-4.70978400	-2.69113400	-1.01043300
C	-6.11597800	-0.90179800	-1.81380300
C	-5.77759400	-3.58062800	-1.11539400
H	-3.75107800	-3.04771000	-0.64457500

```
 C                  -7.18524100   -1.79502600   -1.91965300
 H                  -6.25195400    0.13546500   -2.10281500
 C                  -7.02103000   -3.13505700   -1.57059300
 H                  -5.63863900   -4.62209400   -0.83782000
 H                  -8.14693700   -1.43782400   -2.27830600
 H                  -7.85366300   -3.82857300   -1.65237200
 H                  -1.87609000   -0.27494600    0.03392200
 C                  -3.43651000   -0.86696500    1.36890400
 C                  -2.57435900   -1.78039600    1.99135000
 C                  -4.71091900   -0.64822100    1.90945400
 C                  -2.98864900   -2.47492300    3.13007200
 H                  -1.57359500   -1.92600800    1.58906200
 C                  -5.11816600   -1.33698900    3.05060600
 H                  -5.38327900    0.06528000    1.44205800
 C                  -4.26008300   -2.25554900    3.66158100
 H                  -2.31110600   -3.17786600    3.60776600
 H                  -6.10682700   -1.15629200    3.46379300
 H                  -4.58007700   -2.79058000    4.55188200
 C                  -3.99377700    1.10768200   -1.27330500
 O                  -4.48143800    1.90404200   -2.02958300
 C                  -2.65791700   -0.70438600   -2.40306900
 H                  -2.28982800   -1.73092200   -2.30964500
 H                  -3.13640300   -0.59502400   -3.38113600
 H                  -1.79979800   -0.02499300   -2.35511400
 C                  -1.48742500    3.23402000   -0.42331100
 F                  -0.77532300    2.18855200   -0.86340200
 F                  -0.67808200    4.08231600    0.21572300
 F                  -2.05016500    3.84626600   -1.45996900

H C O N F S 0
6-31G*
****
Fe 0
LANL2DZ
****

Fe 0
LANL2DZ
```

Product L′ + catalyst

```
%nprocshared=16
%mem=800MB
# opt freq=noraman b3lyp/gen pseudo=read

Title Card Required

0 1
 C                   4.23384300   -2.77213600   -0.15311800
 C                   3.22804000   -2.87073500    0.86869300
 C                   3.37896400   -1.75033100    1.74904600
 C                   5.01559200   -1.59329800    0.10537500
```

C	4.48643400	-0.96110000	1.27756800
Fe	3.01925500	-1.09727100	-0.19056700
C	4.47657600	4.60272800	-1.17958000
C	4.54488400	4.90681800	0.32398400
C	3.33201300	4.15187700	0.88013900
N	3.20945200	2.98534500	-0.00115300
C	4.01244200	3.13860500	-1.22276800
C	2.28147300	2.00647900	0.22622500
C	1.35669500	2.10659700	1.27119000
C	0.36156200	1.12132500	1.46677100
N	0.20011400	0.02560300	0.74893000
C	1.10394600	-0.12357600	-0.27094600
C	2.16542700	0.81987900	-0.59266900
H	5.42869200	4.75067400	-1.69771800
H	3.72932400	5.24576600	-1.65958900
H	5.46947300	4.50210500	0.75310900
H	4.50844900	5.97599600	0.55189200
H	3.46986700	3.83547500	1.92059900
H	2.42187100	4.77071400	0.83960300
H	4.87211000	2.45279700	-1.21654600
H	3.41467000	2.92190000	-2.11270500
H	1.34305700	2.97472800	1.91837100
H	-0.36045900	1.27878900	2.26703100
C	2.24484600	-0.93633800	-2.10911500
H	2.55608600	-1.57070900	-2.92915800
C	2.88478600	0.27854500	-1.72282600
H	3.73892600	0.70865200	-2.22231600
C	1.18894400	-1.21427900	-1.19578300
H	0.53459400	-2.07523600	-1.20006300
C	4.47591900	-3.76496400	-1.25472400
H	5.17944900	-4.54728700	-0.93607800
H	3.55133300	-4.26495200	-1.56150900
H	4.90187600	-3.28748100	-2.14336400
C	6.21034600	-1.14101700	-0.68536200
H	7.13596100	-1.59329900	-0.30187200
H	6.12515300	-1.42048700	-1.74066300
H	6.33766400	-0.05421100	-0.64171600
C	5.02495300	0.27410900	1.94190800
H	5.75067700	0.01706500	2.72646500
H	5.53426800	0.92906500	1.22763700
H	4.22668700	0.85783100	2.41132500
C	2.54771300	-1.46867800	2.96716300
H	2.94950800	-1.98409700	3.85104300
H	2.51636800	-0.39868600	3.19400700
H	1.51338000	-1.79657400	2.82733100
C	2.21724500	-3.97257100	1.01314700
H	1.96240600	-4.41525800	0.04486900
H	2.59915000	-4.78033500	1.65360200
H	1.28899400	-3.60640000	1.46290400
C	-4.11267300	-1.32521600	-0.31450600
S	-3.50592100	2.41738100	-0.46406700
O	-4.68525700	3.12646200	-0.93490300
O	-2.75563600	2.80806600	0.72179100
N	-3.87260800	0.78008300	-0.31003700

```
 C                  -3.25855600   -0.28440200    0.54705400
 H                  -2.18750700   -0.37997100    0.34738800
 C                  -3.49704100   -0.16819000    2.02988500
 C                  -2.56960300   -0.76270000    2.89744800
 C                  -4.61312300    0.48251500    2.57209300
 C                  -2.76919400   -0.73101200    4.27876300
 H                  -1.67486900   -1.22054400    2.48288000
 C                  -4.80744000    0.52085900    3.95286400
 H                  -5.32373300    0.97588700    1.91509200
 C                  -3.89080100   -0.09214100    4.81020800
 H                  -2.04050100   -1.19401500    4.93895900
 H                  -5.67412100    1.03536000    4.35903500
 H                  -4.04375400   -0.06036900    5.88560600
 C                  -4.56100200   -0.11567800   -1.15989600
 O                  -5.21686400    0.06256800   -2.15043500
 C                  -2.28955900    2.39702200   -1.89012200
 F                  -1.27709500    1.57887500   -1.59926900
 F                  -1.82749300    3.63404900   -2.07412000
 F                  -2.89800800    1.97438700   -2.99660700
 C                  -3.28712000   -2.36196500   -1.06307400
 C                  -3.46192400   -2.58839600   -2.43477700
 C                  -2.37569100   -3.16050700   -0.35622200
 C                  -2.73231000   -3.58624700   -3.08565000
 H                  -4.16909500   -1.98364600   -2.99318500
 C                  -1.65140200   -4.15991500   -1.00647400
 H                  -2.23263900   -3.00165800    0.70939900
 C                  -1.82603700   -4.37475900   -2.37622000
 H                  -2.87673900   -3.74565600   -4.15088800
 H                  -0.95241900   -4.77133000   -0.44164300
 H                  -1.26277500   -5.15345500   -2.88355600
 C                  -5.29717600   -1.99027300    0.40492000
 H                  -4.93524500   -2.72416900    1.13196500
 H                  -5.91912000   -1.26283400    0.93360600
 H                  -5.91815100   -2.51057400   -0.33088400

 H C O N F S 0
6-31G*
****
Fe 0
LANL2DZ
****

Fe 0
LANL2DZ
```
